# Generation of Oxidoreductases with Dual Alcohol Dehydrogenase and Amine Dehydrogenase Activity

**DOI:** 10.1002/chem.202003140

**Published:** 2020-12-09

**Authors:** Vasilis Tseliou, Don Schilder, Marcelo F. Masman, Tanja Knaus, Francesco G. Mutti

**Affiliations:** ^1^ Van't Hoff Institute for Molecular Sciences, HIMS-Biocat University of Amsterdam Science Park 904 1098 XH Amsterdam The Netherlands

**Keywords:** alcohol amination, alcohol dehydrogenases, amine dehydrogenases, biocatalysis, enzyme promiscuity

## Abstract

The l‐lysine‐*ϵ*‐dehydrogenase (LysEDH) from *Geobacillus stearothermophilus* naturally catalyzes the oxidative deamination of the *ϵ*‐amino group of l‐lysine. We previously engineered this enzyme to create amine dehydrogenase (AmDH) variants that possess a new hydrophobic cavity in their active site such that aromatic ketones can bind and be converted into α‐chiral amines with excellent enantioselectivity. We also recently observed that LysEDH was capable of reducing aromatic aldehydes into primary alcohols. Herein, we harnessed the promiscuous alcohol dehydrogenase (ADH) activity of LysEDH to create new variants that exhibited enhanced catalytic activity for the reduction of substituted benzaldehydes and arylaliphatic aldehydes to primary alcohols. Notably, these novel engineered dehydrogenases also catalyzed the reductive amination of a variety of aldehydes and ketones with excellent enantioselectivity, thus exhibiting a dual AmDH/ADH activity. We envisioned that the catalytic bi‐functionality of these enzymes could be applied for the direct conversion of alcohols into amines. As a proof‐of‐principle, we performed an unprecedented one‐pot “hydrogen‐borrowing” cascade to convert benzyl alcohol to benzylamine using a single enzyme. Conducting the same biocatalytic cascade in the presence of cofactor recycling enzymes (i.e., NADH‐oxidase and formate dehydrogenase) increased the reaction yields. In summary, this work provides the first examples of enzymes showing “alcohol aminase” activity.

## Graphical abstract

**Lend me your hydrogen**: Starting from an *ϵ*‐deaminating l‐lysine dehydrogenase, we created new variants that perform the reduction of aldehydes to alcohols and the reductive amination of aldehydes and ketones to amines. The reductive amination of ketones proceeded with excellent stereoselectivity. This dual AmDH/ADH activity was applied for the one‐enzyme hydrogen‐borrowing amination of benzylic alcohol, thus providing the first example of an “alcohol aminase” activity.

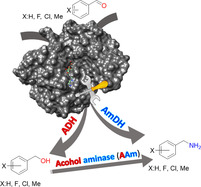

## Introduction

The concept of enzyme promiscuity refers to an enzyme's ability to catalyze mechanistically distinct reactions (i.e., catalytic promiscuity) or convert structurally diverse substrates by following the same mechanism (i.e., substrate promiscuity).^[1]^ The promiscuous catalytic behavior of enzymes has been harnessed for chemical synthesis and has served for the evolution of variants possessing novel catalytic activities and/or enhanced substrate scope.^[2]^ In some cases, the enzyme's catalytic or substrate promiscuity can be simply tuned by changing the reaction conditions (i.e., condition promiscuity), a classical example of which is the catalytic activity of hydrolases in non‐aqueous media wherein (*trans*)esterification and amidation reactions of carboxylic acids and esters are enabled.^[3]^

Alcohol dehydrogenases (ADHs, EC 1.1.1.X) from the nicotinamide adenine dinucleotide‐ or nicotinamide adenine dinucleotide phosphate‐dependent [NAD(P)] category catalyze reversible interconversion between alcohols and carbonyl compounds.^[4]^ These enzymes have mainly been studied for the asymmetric reduction of ketones to corresponding chiral alcohols, as they often exhibit excellent and complementary stereoselectivity along with a broad substrate scope.[^4a^, ^4f^, ^4g^] In this context, ADHs are currently the second most applied enzyme family after the hydrolases in industrial chemical manufacturing.^[4a]^ In contrast, ADHs have been less frequently applied for the oxidation of alcohols to ketones or aldehydes, although this is also a synthetically useful biocatalytic transformation.[^4b^, ^4d^, ^4e^] For instance, ADH‐catalyzed oxidation of secondary alcohols can be exploited for the kinetic resolution or even deracemization of racemic alcohol mixtures.^[5]^ Furthermore, ADHs can oxidize primary alcohols to aldehydes with exquisite chemoselectivity, whereas other oxidoreductases such as alcohol oxidases commonly catalyze over‐oxidation to carboxylic acids or other compounds.[^2g^, ^6^] Another synthetic application of ADHs in oxidation reactions—in particular those involving primary alcohols—entails their implementation into linear multi‐enzymatic cascade reactions for the production of lactones, lactams, amines and other high‐value compounds.[^4d^, ^7^] Notably, only a handful of NAD(P)‐dependent ADHs have been biochemically characterized and synthetically applied for the chemoselective conversion of primary alcohols to aldehydes and vice versa, namely from horse liver (UniProt P00327),^[8]^ yeast (P00330),[^8a^, ^9^] *Bacillus stearothermophilus* (UniProt P42328, PDB 1RJW; 3PII),^[10]^ *Sulfolobus solfataricus* (UniProt P39462, PDB 1R37),^[11]^ *Acinetobacter calcoaceticus* (UniProt Q59096, PDB 1F8F),^[12]^ and *Thermoanaerobacter brockii* (also named as *Thermoanaerobacter ethanolicus*, UniProt P14941, PDB 1YKF) and variants thereof.[^8c^, ^13^] Conversely, other oxidations of primary alcohols to aldehydes rely on whole cell systems.^[6c]^

Amine dehydrogenases (AmDHs, EC 1.4.1.X) catalyze the reversible reductive amination of carbonyl compounds at the sole expense of ammonia and NAD(P)H, the latter of which is applied in a catalytic amount and recycled with established methods.^[14]^ Since the pioneering work of Bommarius’ group, the toolbox of AmDHs for the synthesis of enantiopure α‐chiral amines has been significantly expanded either by the engineering of l‐amino acid dehydrogenases or the discovery of native AmDHs using (*meta*)genomic data (for the latter group UniProt entries: A0A0D6I8P6; K0UKT5; A0A101AWU7; C3UMY1; E3CZE3; S9Q235).[^14a^, ^15^] Our group has discovered the catalytic promiscuity of AmDHs for the synthesis of secondary or tertiary amines, and other groups have also recently investigated this property.^[16]^

In this context, we recently generated a new family of AmDHs by engineering a particular l‐lysine‐dehydrogenase (LysEDH, UniProt Q9AJC6), whose natural reaction is the *ϵ*‐amino oxidative deamination of l‐lysine.^[15k]^ The best variant possessed a F173A single mutation, which created a new hydrophobic cavity in the active site wherein aromatic ketones can be accommodated and converted into α‐chiral amines with excellent enantioselectivity. Notably, we recently observed that the wild type LysEDH is also capable of producing primary alcohols starting from aromatic aldehydes. The capability of an oxidoreductase to reduce both C=O and C=N bonds was rarely observed until to date. Müller's group reported the promiscuous imine reductase activity of a glucose dehydrogenase.^[2s]^ They later discovered that a short chain dehydrogenase/reductase (SDR)—namely the noroxomaritidine reductase from *Narcissus pseudonarcissus* (NR)—could reduce C=C (i.e., of enones), C=O and C=N bonds, whereas another SDR from *Zephyranthes treatie* also exhibited dual C=O and C=N activity.^[17]^ Furthermore, a SDR from *Methylobacterium* sp. 77 that only exhibits ketoreductase activity was recently engineered to gain imine reductase activity by introducing four mutations in its active site to resemble some of the structural features of the NR reductase.^[18]^ Notably, in all of these cases, the ketoreductase and the imine reductase activities were strictly substrate dependent; furthermore, the SDR enzymes were active toward pre‐formed (cyclic) imines but reductive amination between a carbonyl compound and an amine donor was not reported. Conversely, other groups have independently reported that few imine reductases (IReds) and reductive aminases (RedAms) possess promiscuous ketoreductase activity on very specific substrates such as tri‐, di‐ and mono‐fluorinated acetophenones at the terminal carbon position.^[19]^

In the present work, we studied the catalytic promiscuity and exploited the high evolvability of LysEDH to create new variants that possess enhanced ADH activity or even both AmDH activity (i.e., for the reductive amination of carbonyl compounds with free ammonia) and ADH activity. The best variant exhibiting dual ADH‐AmDH activity was harnessed to accomplish the first example of one‐enzyme hydrogen‐borrowing amination of benzylic alcohol.

## Results and Discussion

### Compound selection

**Figure 1:**
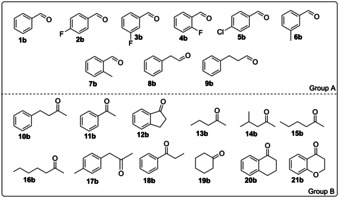
List of compounds used in this study. Groups A and B depict the aldehydes and ketones, respectively, that were tested for both reductive amination and reduction to alcohols.

**Scheme 1:**
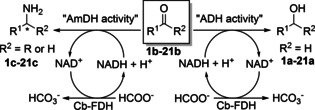
Dual alcohol dehydrogenase (ADH)/amine dehydrogenase (AmDH) activity of LysEDH variants.

We conducted this study with a panel of aldehydes and ketones as depicted in Figure 1. Group A comprises substituted benzaldehydes and arylaliphatic aldehydes, whereas Group B comprises aromatic, arylaliphatic and aliphatic ketones. We tested aldehydes (20 mm) or ketones (10 mm) for reduction to the corresponding alcohols and reductive amination to the corresponding primary amines, the latter in the presence of 2 m ammonium/ammonia species (**1 b**–**21 b**, Scheme 1).

However, biocatalytic transformations of aldehydes can sometimes be complicated due to their volatility and limited extractability from an aqueous medium. Therefore, we initially checked if the selected aldehydes (Figure 1 and Supporting Information Figure S1, **1 b**–**9 b** and **22 b**–**26 b**), the corresponding primary alcohols (**1 a**–**9 a** and **22 a**–**26 a**), and terminal amines (**1 c**–**9 c** and **22 c**–**26 c**) were not excessively volatile during incubation for 24 h and could then be efficiently extracted from the aqueous buffer (see Supporting Information section 3 for details). Aldehydes that could be recovered with analytical yields between 66 % and 90 % upon incubation in an aqueous buffer (see Supporting Information, Table S1 for details) were considered in this study (Figure 1, Group A). However, although they were recovered in 79 % and 88 % analytical yields, respectively (Supporting Information, Table S1), aldehydes **25 b** and **26 b** were not included in this study because the related alcohols (**25 a**, **26 a**) and amines (**25 c**, **26 c**) that are attainable from the reduction of aldehydes were recovered in only an 8 % yield or not recovered at all following incubation for 24 h. In contrast, all of the selected ketones (Figure 1, Group B) could be included in this study because we did not observe any significant volatility or extractability issues.

### Reductive amination of aldehydes

The reductive amination of aldehydes was performed using wild‐type LysEDH, LE‐AmDH‐v1^[15k]^—the latter of which exhibited high activity toward the reductive amination of benzaldehyde—and four additional LE‐AmDH variants that were originally designed to reductively aminate bulky‐bulky ketones. These four new variants were generated by mutating the amino residues of LysEDH (i.e., Y238A and/or T240A) that are involved in the interaction between the enzyme's active site and the *α*‐amino group of the natural substrate l‐lysine. We combined these new mutations with either the F173S or F173A mutation, the latter of which was already present in the LE‐AmDH first generation variants;^[15k]^ this resulted in LE‐AmDH‐v22 (LysEDH Y238A/F173A), LE‐AmDH‐v24 (Y238A/F173S), LE‐AmDH‐v25 (Y238A/T240A/F173A), and LE‐AmDH‐v27 (Y238A/T240A/F173S). Detailed information regarding primers for mutations and biocatalysts preparation is reported in Supporting Information section 4. Notably, LE‐AmDH‐v22 and v25 possess the F173A mutation as the first‐generation variant LE‐AmDH‐v1; therefore, these new variants could possibly retain the amine dehydrogenase activity of the parent LE‐AmDH‐v1 toward aromatic substrates. Our previous study also demonstrated that the LE‐AmDH enzyme family catalyzes a genuine reductive amination by sourcing the carbonyl compound and ammonia/ammonium from the reaction medium. For instance, LE‐AmDH‐v1 exhibited high activity for the reductive amination of benzaldehyde (**1 b**) with NH_3_/NH_4_^+^ at pH 7.8 (50 mm **1 b**, >99 % conversion).^[15k]^ In this context, Nestl's group showed that spontaneous imine formation from **1 b** in aqueous medium is negligible (or does not occur at all) at pH levels below 8.^[20]^ Furthermore, LE‐AmDH‐v1 exhibited high activity for the reductive amination of acetophenone (**11 b**) with NH_3_/NH_4_^+^ at pH levels ranging from 7 to 9.5.^[15k]^ Under such conditions, the spontaneous formation of the imine of **11 b** was never observed in an aqueous buffer.^[20]^ These observations confirm that LE‐AmDHs catalyze the reaction between a carbonyl compound and free NH_3_/NH_4_^+^ along with a possible reduction of any pre‐formed aldimine in solution at more basic pH values.

Finally, the F173S mutation was introduced into LE‐AmDH‐v24 and ‐v27 in order to investigate the effects of a hydrophilic, non‐bulky residue in this position. In all of the reactions, two samples were included as negative controls (NC1 and NC2, respectively). NC1 only contained the recombinant formate dehydrogenase from *Candida boidinii* (Cb‐FDH) and NAD^+^ to verify that neither amine nor alcohol product formation is obtained as the result of a possible promiscuous catalytic activity of the cofactor‐recycling enzyme. NC2 contained none of the enzymes, and only NAD^+^ and the aldehyde substrate were added to the buffer. Table 1 summarizes the results obtained for the reductive amination reactions of selected aldehydes **1 b**–**9 b** (20 mm; see Experimental Section and Supporting Information Section 5.1 for details on biocatalytic reactions and quantitative analytical determination). The reactions were run at 30 °C in an ammonium formate buffer (2 m, pH 8.2–9) in the presence of NAD^+^ (1 mm), Cb‐FDH (16 μm) and LE‐AmDH enzyme (45 μm). It is evident that all of the variants possessing the F173A mutation (v1, v22 and v25) performed better in the reductive amination of aldehydes than those possessing the F173S mutation (v24 and v27). In particular, LE‐AmDH‐v22 generally performed slightly better than LE‐AmDH‐v1 and ‐v25, as it yielded the highest amine formation for the reduction of substituted benzaldehydes **3 b**–**7 b** (11‐ >99 %). The reductive amination of benzaldehyde (**1 b**) and *para*‐fluorobenzaldehyde (**2 b**) essentially proceeded equally well with LE‐AmDH‐v1, ‐v22 and ‐v25. Compound **1 b** was the most converted substrate (>99 % yield), whereas **2 b** was aminated in 89–91 % yield by the three variants. However, none of the variants could aminate either phenylacetaldehyde (**8 b**) or 3‐phenylpropanal (**9 b**). As mentioned above, the LE‐AmDH variants containing the F173S mutation (v24 and v27) exhibited a lower capability of aminating aldehydes, although LE‐AmDH‐v24 generally yielded slightly higher amine formation for the reduction of **1 b**, **3 b** and **4 b** (11–80 %). Notably, we did not observe any amine formation in any of the negative control reactions (NC1 and NC2), thereby proving that only the LE‐AmDHs catalyze the reductive amination of aldehydes.

**Table 1:** Reductive amination of aromatic aldehydes catalyzed by LE‐AmDH variants.^[a]^

% Analytical yields of amine^[b]^ and *(alcohol)* ^[c]^						
Sub.	WT	v1	v22	v24	v25	v27
**1 b**	17 *(23)*	>99 *(n.d.)*	>99 *(n.d.)*	80 *(n.d.)*	>99 *(n.d.)*	52 *(22)*
**2 b**	23 *(28)*	90 *(n.d.)*	89 *(n.d.)*	34 *(n.d.)*	91 *(n.d.)*	36 *(28)*
**3 b**	n.d. *(12)*	74 *(n.d.)*	99 *(n.d.)*	11 *(6)*	74 *(n.d.)*	4 *(30)*
**4 b**	2 *(n.d.)*	98 *(n.d.)*	>99 *(n.d.)*	54 *(n.d.)*	>99 *(n.d.)*	44 *(n.d.)*
**5 b**	n.d. *(n.d.)*	3 *(n.d.)*	11 *(n.d.)*	1 *(4)*	5 *(n.d.)*	n.d. *(18)*
**6 b**	n.d. *(n.d.)*	6 *(n.d.)*	13 *(n.d.)*	n.d. *(n.d.)*	8 *(n.d.)*	n.d. *(n.d.)*
**7 b**	n.d. *(n.d.)*	91 *(n.d.)*	92 *(n.d.)*	6 *(n.d.)*	87 *(n.d.)*	6 *(n.d.)*
**8 b**	n.d. *(n.d.)*	n.d. *(n.d.)*	n.d. *(n.d.)*	n.d. *(n.d.)*	n.d. *(n.d.)*	n.d. *(n.d.)*
**9 b**	n.d. *(20)*	n.d. *(n.d.)*	n.d. *(9)*	n.d. *(9)*	n.d. *(n.d.)*	n.d. *(33)*

[a] Experimental conditions: 1 mL final volume in Eppendorf tubes; buffer: ammonium formate (2 m, pH 8.2–9.0); *T*: 30 °C; reaction time: 24 h; agitation orbital shaker (170 rpm); [substrate]: 20 mm; [NAD^+^]: 1 mm; [LysEDH or LE‐AmDH variant]: 45 μm; [Cb‐FDH]: 16 μm. NC 1: reaction without LysEDH or LE‐AmDH variant; NC 2: reaction without any enzyme addition (LysEDH or LE‐AmDH, and FDH). In all cases, NC1 and NC2 resulted in no detectable analytical yields for amines or alcohols. [b] The reported yields (%) are obtained from the average values obtained from two independent experiments. [c] Numbers in parentheses indicate the analytical yields (%) of the alcohols formed as by‐products. n.d.: not detected.

Surprisingly, we also observed that the wild‐type LysEDH and both variants containing the F173S mutation produced significant amounts of alcohol by‐products (4–33 %) in a number of biocatalytic reductions in ammonium buffers. In particular, the alcohol yield was greater than the amine formation for the reductions of benzaldehyde and derivatives **1 b**–**3 b** and 3‐phenylpropanal (**9 b**) catalyzed by LysEDH. The alcohols were also the main products of the reductions of **3 b** and **9 b** catalyzed by LE‐AmDH‐v27. Despite the presence of ammonium species in the reaction medium, 3‐phenylpropanol (**9 a**) was the only product of the reduction of **9 b** catalyzed by LysEDH, LE‐AmDH‐v22, ‐v24 and ‐v27, whereas LE‐AmDH‐v1 was inactive toward **9 b**. LE‐AmDH‐v27 produced the highest alcohol yield (18–33 %) in an ammonium buffer for all the substrates that exhibited this behavior (**1 b**–**3 b**, **5 b**, **9 b**). When incubated with **1 b** and **2 b**, wild‐type LysEDH produced equal amounts of alcohol as LE‐AmDH‐v27 (23 % and 28 % alcohol yields, respectively). Conversely, with the exception of LE‐AmDH‐v22 with **9 b** (9 % alcohol yield), none of the reactions catalyzed by LE‐AmDHs variant possessing the F173A mutation (v1, v22 and v25) resulted in any alcohol formation. Moreover, alcohol formation was never observed in the negative control reactions (NC1 and NC2), thereby proving that the introduction of F173S mutation somehow promoted promiscuous alcohol formation. This phenomenon was further confirmed in biocatalytic reactions in which the reductions were run in an ammonia‐free environment (described later), thus precluding any amine product formation.

### Reductive amination of ketones

We investigated stereoselective reductive amination catalyzed by LE‐AmDH‐v22, ‐v24, ‐v25 and ‐v27 with ketone substrates **10 b**–**21 b** (Scheme 1, Group B). LysEDH and LE‐AmDH‐v1 were tested for the reductive amination of these compounds in our previous study. LysEDH did not exhibit any amination activity toward these ketones, whereas the previous results obtained with LE‐AmDH‐v1 are reported again in Table 2 to enable a better comparison with the other variants.^[15k]^ The ketone amination reactions were performed at 50 °C for 48 h because LE‐AmDH‐v1 exhibited high thermal stability and accelerated kinetic behavior at higher temperatures in our previous study.^[15k]^ In contrast, the temperature was set at 30 °C for the previously described reductive amination with aldehydes to limit their evaporation during the reaction. Substrates, enzymes and coenzyme concentrations were the same as previously used in both the sample reactions and the negative controls (NC1 and 2). The reductive amination of ketones resulted in generally higher analytical yields than those for the conversion of aldehydes. However, this result must be at least partly attributed to the lower volatility and higher chemical stability of ketones compared with aldehydes in aqueous buffers at pH 8.2–9. Table 2 shows that LE‐AmDH‐v22 and ‐v1 were the most active variants for the reductive amination of ketones, which is in agreement with the results obtained for the reductive amination of aldehydes. Among the ketone substrates that were converted by the two variants, the highest yields ranged from 76 % to >99 %. An exception was the reductive amination of 2‐heptanone (**16 b**) with a maximum of 13 % analytical yield, whereas 4‐phenyl‐butan‐2‐one (**10 b**) and *para*‐methyl‐phenylacetone (**17 b**) were not converted at all. In particular, LE‐AmDH‐v1 afforded a significantly higher analytical yield for the reductive amination of 2‐pentanone (**13 b**, 86 % vs. 52 %), whereas LE‐AmDH‐v22 afforded higher analytical yield for the amination of 4‐methyl‐pentan‐2‐one (**14 b**, 83 % vs. 76 %), 2‐hexanone (**15 b**, 93 % vs. 87 %) and 4‐chromanone (**21 b**, 99 % vs. 82 %). The reductive aminations of all of the other ketones—acetophenone (**11 b**), 1‐indanone (**12 b**), **16 b**, propiophenone (**18 b**), cyclohexanone (**19 b**) and 1‐tetralone (**20 b**)—resulted in the same or very similar analytical yields when using either LE‐AmDH‐v1 or ‐v22. Table 2 also shows that LE‐AmDH‐v25 yielded lower amine formation than LE‐AmDH‐v22. Compared with LE‐AmDH‐v22 (i.e., wild‐type LysEDH Y238A/F173A), LE‐AmDH‐v25 has an additional T240A mutation, which was therefore detrimental for catalytic activity toward the ketone substrates. The only exception was observed for the reductive amination of the more sterically demanding ketone **17 b**, which bears a methyl substituent in *para* position at the phenyl ring. In this case, LE‐AmDH‐v25 converted **17 b** (5 % yield), whereas catalytic activity was not detected with LE‐AmDH v22 and ‐v1. As the catalytic behavior of LE‐AmDH‐v25 could have resulted from the larger volume of its active site due to the additional T240A mutation, we conducted Michaelis–Menten kinetic experiments on ketones **14 b** and **15 b** using LE‐AmDH‐v22 and ‐v25 (see Supporting Information section 8, Table S10). However, comparing the catalytic parameters for the reductive amination catalyzed by the two variants, LE‐AmDH‐v22 exhibited the highest affinity for **15 b** (*K*_M app_ 4.70±0.55 mm vs. 6.50±0.55 mm) whereas LE‐AmDH‐v25 exhibited the highest affinity for **14 b** (*K*_M app_ 3.38±0.33 mm vs. 9.00±0.80 mm). Therefore, LE‐AmDH‐v22 afforded higher conversions than v25 for the reduction amination of **14 b** and **15 b** essentially because of the higher *k*_app_ values (for **14 b**: *k*_app_ 0.29±0.01 min^−1^ vs. 0.24±0.01 min^−1^; for **15 b**: *k*_app_ 0.67±0.02 min^−1^ vs. 0.24±0.01 min^−1^).

**Table 2:** Reductive amination of aromatic ketones catalyzed by LE‐AmDH variants.^[a]^

% Analytical yields of amine and *(ee)* ^[b]^					
Sub.	v1^[c]^	v22	v24	v25	v27
**10 b**	n.d. *(n.m.)*	n.d. *(n.m.)*	n.d. *(n.m.)*	n.d. *(n.m.)*	n.d. *(n.m.)*
**11 b**	>99 *(>99)*	97 *(>99)*	50 *(>99)*	84 *(>99)*	34 *(>99)*
**12 b**	74 *(>99)*	76 *(>99)*	11 *(>99)*	62 *(>99)*	11 *(>99)*
**13 b**	86 *(89)*	52 *(89)*	3 *(n.m.)*	32 *(>99)*	1 *(n.m.)*
**14 b**	76 *(97)*	83 *(>99)*	n.d. *(n.m.)*	53 *(>99)*	n.d. *(n.m.)*
**15 b**	87 *(>99)*	93 *(>99)*	8 *(n.m.)*	53 *(>99)*	n.d. *(n.m.)*
**16 b**	10^[d]^ *(n.m.)*	13 *(n.m.)*	n.d. *(n.m.)*	10 *(n.m.)*	n.d. *(n.m.)*
**17 b**	n.d. *(n.m.)*	n.d. *(n.m.)*	n.d. *(n.m.)*	5 *(n.m.)*	1 *(n.m.)*
**18 b**	99 *(>99)*	97 *(>99)*	50 *(>99)*	84 *(>99)*	34 *(>99)*
**19 b**	86. *(n.a.)*	85 *(n.a.)*	5 *(n.a.)*	75 *(n.a.)*	7 *(n.a.)*
**20 b**	79 *(>99)*	80 *(>99)*	9 *(>99)*	64 *(>99)*	12 *(>99)*
**21 b**	82 *(>99)*	99 *(>99)*	41 *(>99)*	85 *(>99)*	27 *(>99)*

[a] Experimental conditions: 0.5 mL final volume in Eppendorf tubes; buffer: ammonium formate (2 m, pH 9.0); *T*: 50 °C; reaction time: 48 h; agitation orbital shaker (170 rpm); [substrate]: 10 mm; [NAD^+^]: 1 mm; [LE‐AmDH variant]: 90 μm; [Cb‐FDH]: 16 μm. NC 1: reaction without LysEDH or LE‐AmDH variant; NC 2: reaction without any enzyme addition (LysEDH or LE‐AmDH, and FDH). In all cases, NC1 and NC2 resulted in no detectable analytical yields for amines and alcohols. [b] Numbers in parentheses indicate the *ee* (%) of the amines formed. n.d.: not detected; n.m.: *ee* not measured due to too low conversion; n.a.: not applicable (non‐chiral product). [c] Data reported for reductive amination catalyzed by LE‐AmDH‐v1 are from Ref. [15k]. [d] The reaction was run at 30 °C.

Finally, the two variants possessing the F173S mutation (LE‐AmDH‐v24 and LE‐AmDH‐v27) exhibited dramatically lower conversion values, thereby indicating that introducing the hydrophilic serine in the enzyme's binding cavity hampers the accommodation of hydrophobic ketone substrates.

The stereoselective outcome of the reductive amination was measured for all cases in which sufficient conversion was achieved. Notably, there was always at least one LE‐AmDH variant that could produce the amine products with >99 % *ee* (*R*). In general, the stereoselectivity of the reaction was always perfect except for LE‐AmDH‐v1 with **13 b** and **14 b** and LE‐AmDH‐v22 with **13 b**.

### Investigation on the promiscuous reduction of aldehydes to alcohols

**Figure 2:**
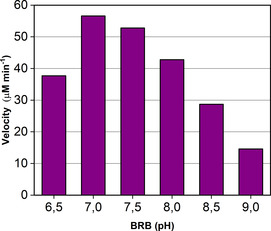
pH optimum study for the reduction of benzaldehyde (**1 b**, 20 mm) to benzyl alcohol (**1 a**) in Britton‐Robinson's universal buffer (varied pH) and catalyzed by LE‐AmDH‐v27 (45 μm). The NAD^+^ coenzyme (1 mm) was recycled using Cb‐FDH (14 μm) and HCOONa (100 mm). Reactions were run at 30 °C and shaken at 170 rpm on an orbital shaker.

As mentioned above, the reductive amination of aldehydes catalyzed by wild‐type LysEDH and variants LE‐AmDH‐v24 and ‐v27 led to alcohol product formation although the reaction medium contained ammonia/ammonium ions. In particular, LE‐AmDH‐v27 produced the highest amount of alcohol among all of the tested variants. Therefore, we envisioned that promiscuous alcohol formation catalyzed by LE‐AmDH‐27 could be enhanced in an ammonia‐free environment, in which the imine intermediate for the reductive amination cannot be generated. Therefore, we investigated the reduction of the test substrate benzaldehyde (**1 b**, 20 mm) to benzyl alcohol (**1 a**) catalyzed by LE‐AmDH‐v27 (45 μm). The optimum of pH for this biocatalytic transformation was initially investigated using the Britton‐Robinson universal buffer in pH levels ranging from 6.5–9.0. Results showed that the highest velocity (57 μm min^−1^) was obtained at pH 7 (Figure 2; see Supporting Information section 5.2, Table S3 for details). Conversely, we previously reported that the reductive amination reaction proceeds better at higher pH values, namely pH 9‐9.5.^[15k]^ However, Figure 2 shows that the apparent rate for the reduction of **1 b** to **1 a** decreased approximately 4‐fold at pH 9‐9.5 compared with the apparent rates obtained at pH 7.

In the next step, the progress of the reduction of **1 b** to **1 a** was monitored using five different types of buffers (HEPES, Tris, MOPS, KPi, NaPi) at pH 7 and 100 mm concentration (see Supporting Information section 5.3 and Figure S3). These results showed that LE‐AmDH‐v27 has no apparent preference for a certain type of buffer because the reaction progress curves essentially overlapped and the reactions always resulted in ≥98.6 % conversion after 24 h (corresponding to ≥18.8 mm of formed **1 a**). Therefore, we decided to continue our study using the KPi buffer.

Using the optimal reaction conditions for the reduction of **1 b** to **1 a** (KPi buffer 100 mm, pH 7; LE‐AmDH variant 45 μm, Cb‐FDH 16 μm, NAD^+^ 1 mm, HCOONa 100 mm), we investigated the promiscuous alcohol dehydrogenase activity of the LE‐AmDH variants. Table 3 reports the results for the reduction of aromatic and arylaliphatic aldehydes (Scheme 1, group A) to the corresponding primary alcohols using the six selected variants (see Experimental Section and Supporting Information section 5.4 for details). Benzaldehyde (**1 b**) and benzaldehydes possessing a substituent such as a chloro or a methyl group in *meta* or *para* position at the phenyl ring (**2 b**, **3 b**, **5 b**, **6 b**) were generally converted with moderate or excellent yields by all of the enzymes. LE‐AmDH‐v27 was the best performing enzyme for the reduction of **2 b**, **5 b** and **6 b** (80 %, >99 %, >99 % conversions, respectively), whereas LE‐AmDH‐v24 and wild‐type LysEDH performed best with *meta*‐fluorobenzaldehyde (**3 b**, 94 % conversion) and benzaldehyde (**1 b**, 82 % conversion). Conversely, only LE‐AmDH‐v27 could reduce *ortho*‐fluorobenzaldehyde (**4 b**) to the corresponding alcohol with a moderate yield (40 %); the other variants exhibited poor activity (max 11 % conversion). Furthermore, none of the enzymes could convert *ortho*‐methylbenzaldehyde (**7 b**). These results are different from the data obtained for the reductive amination reactions in which LE‐AmDH‐v1, ‐v22 and ‐v25 could aminate **4 b** with 98‐ >99 % conversions and **7 b** with 87–92 % conversions (Table 1). Therefore, the low or lack of catalytic activity for the reduction of **4 b** and **7 b** to the corresponding alcohols **4 a** and **7 a** by the LE‐AmDH variants cannot be attributed to particular steric (i.e., for **7 b**) or electronic (i.e., for **4 b**) effects. The different reactivity is likely due to the varying distances and orientations between the prochiral carbon atom of the ligand (i.e., ketone substrate for ADH reaction or iminium intermediate for AmDH reaction) and the departing hydride of the NADH coenzyme in the enzyme's active site. For instance, analysis of the X‐ray structures of a l‐phenylalanine amino acid dehydrogenase from *Rhodococcus* sp. demonstrated that this critical distance changes from more than 5 Å (i.e., unproductive binding) to ∼3–3.5 Å (i.e., productive binding) when the ketone ligand is converted into its imine/iminium intermediate during the catalytic cycle in the enzyme's active site.^[21]^ Our recent computational studies on the reactivity of AmDHs based on the analysis of this critical distance as well as substrate/intermediate orientation (i.e., through a defined dihedral angle) further support this interpretation.^[16a]^ In another study, Grogan's and Turner's groups crystallized a reductive aminase (AtRedAm) with a ketone substrate and NADPH coenzyme in the active site, and a 4.5 Å distance was observed between the ketone's prochiral carbon and the hydride of NADPH, thereby precluding any substrate reduction.^[22]^

**Table 3:** Reduction of aromatic aldehydes to alcohols by LE‐AmDH variants.^[a]^

% Analytical yield of alcohol^[b]^								
Sub.	WT	v1	v22	v24	v25	v27	NC1	NC2
**1 b**	82	76	54	66	56	77	7	n.d.
**2 b**	74	64	59	53	46	80	5	n.d.
**3 b**	83	85	80	94	79	86	18	n.d.
**4 b**	11	11	7	9	7	40	1	n.d.
**5 b**	84	65	24	59	21	>99	n.d.	n.d.
**6 b**	59	52	28	51	33	>99	1	n.d.
**7 b**	n.d.	n.d.	n.d.	n.d.	n.d.	n.d.	n.d.	n.d.
**8 b**	n.d.	n.d.	n.d.	n.d.	n.d.	4	n.d.	n.d.
**9 b**	67	68	68	67	68	67	61	n.d.

[a] Experimental conditions: 1 mL final volume in Eppendorf tubes; buffer: KPi (100 mm, pH 7.0); *T*: 30 °C; reaction time: 24 h; agitation orbital shaker (170 rpm); [substrate]: 20 mm; [NAD^+^]: 1 mm; [LysEDH or LE‐AmDH variant]: 45 μm; [Cb FDH]: 16 μm. NC 1: reaction without LysEDH or LE‐AmDH variant; NC 2: reaction without any enzyme addition (LysEDH or LE‐AmDH, and FDH). [b] The analytical yields reported here are the average values obtained from four independent experiments. n.d.: not detected.

Other authors have also attempted to explain the different carbonyl reductase and imine reductase activities of IReds and RedAms based on the determination of Gibbs energy barriers for the hydride transfer.^[19]^ Although these calculations correlated with the experimentally observed reduction of 2,2,2‐trifuoroacetophenone to the related alcohol, compared with other non‐converted ketones, an inspection of the X‐ray crystal structure revealed that the two fluorine atoms of the substrate directly interact with one hydroxy group of the ribose of NADP; this interaction pushed the ketone substrate toward NADP in such a manner that a closer distance between the prochiral ketones’ carbon atom and the departing hydride of NADPH was again attained.^[23]^ Our independent calculations on carbonyl reduction (pH 7, 100 mm KPi) and reductive amination (pH 9, 2 m NH_3_/NH_4_^+^) under the experimentally attained reaction conditions show negligible differences of the reaction's Gibbs energy (i.e., for benzaldehyde: reduction Δr*G*’=−45 kJ mol^−1^; reductive amination Δr*G*’=−49 kJ mol^−1^; for acetophenone: reduction Δr*G*’=−45 kJ mol^−1^; reductive amination Δr*G*’=−42 kJ mol^−1^; see Supporting Information section 6 for details). In general, the aforementioned distance between the carbonyl's carbon atom and NAD(P)H's hydride appears to be a critical factor, although other parameters can determine carbonyl vs. imine reductase activities, such as a suitable cofactor domain, proton donors adjusted at suitable p*K*_a_ for efficient imine protonation, flanking residues for p*K*_a_ adjustment, and a negative electrostatic potential in the substrate‐binding site.^[24]^

In the case of the reduction of phenylacetaldehyde (**8 b**), only LE‐AmDH‐v27 was capable of producing the corresponding alcohol **8 a**, albeit in 4 % analytical yield. A very similar result was obtained for the reductive amination of **8 b** (Table 1), for which none of variants was active. Finally, 3‐phenylpropanal (**9 b**)—which possesses one carbon more on its aliphatic chain compared with **8 b**—was apparently converted into the alcohol **9 a** in good analytical yields (67–68 %). However, a 61 % yield was also obtained in negative control reactions that were devoid of LE‐AmDH variant but included Cb‐FDH (NC1). This result indicates that Cb‐FDH, which is used for NADH‐recycling, is mainly or even solely responsible for this transformation. In fact, negative control reactions, which were also devoid of Cb‐FDH (NC2), gave no detectable conversion. Interestingly, Table 3 shows that Cb‐FDH also exhibited a low level of activity for the reduction of **1 b**–**4 b** and **6 b**. However, in these cases, the yields obtained with the LE‐AmDH variants were higher than one or two orders of magnitude, thus confirming promiscuous activity. In summary, Table 3 shows that LE‐AmDH‐v27 (possessing the F173S mutation) gave the highest conversion for the reduction of aldehydes **2 b** (80 %), **4 b** (40 %), **5 b** (>99 %) and **6 b** (>99 %) and was the only enzyme capable of converting **8 b**. Wild‐type LysEDH and LE‐AmDH‐v24 (also possessing the F173S mutation) were, respectively the best performing enzymes for reduction of **1 b** (82 %) and **3 b** (94 %) to the related alcohols. Conversely, this trend was reversed in the reductive amination reaction, for which LE‐AmDH‐v1, ‐v22 and ‐v25 gave the highest analytical yields of amine products **1 c**–**7 c** (Table 1). Therefore, it appears that the F173A mutation favors AmDH catalytic activity, whereas the F173S mutation favors ADH catalytic activity. Finally, comparing the outcomes of the reactions catalyzed by LE‐AmDH‐v24 and ‐v27, it is also evident that an additional alanine mutation in position 240 (T240A) further enhances the alcohol dehydrogenase reaction.

Notably, none of the LE‐AmDH variants could reduce any of the ketones **10 b**–**12 b**, **14 b** and **16 b**–**21 b** (Group B, Figure 1) to the corresponding secondary alcohols. Only ketones **13 b** and **15 b** were reduced albeit with 1 % conversion (see Supporting Information section 7 for details on chromatographic separations). Therefore, the stereoselective outcome of the reaction could not be determined due to the excessively low conversion. The lack of ADH activity might be due to the impossibility of binding the ketone substrates in the enzyme's active site with the correct distance and position for NADH hydride delivery. In contrast, such a positioning is more probable for aldehydes than ketones because the former possess only one carbon chain, thereby increasing the probability of generating one or more productive binding modes.

**Figure 3:**
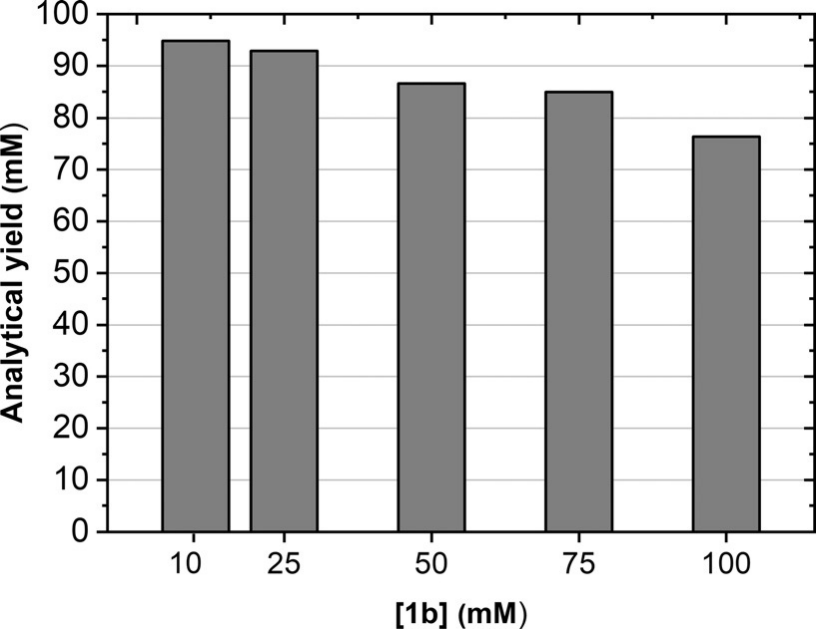
Influence of benzaldehyde (**1 b**) concentration on the analytical yield of the alcohol product (**1 a**). A single set of experiments was performed. Experimental conditions: buffer KPi (100 mm, pH 7.0); *T*: 30 °C; reaction time: 24 h; agitation orbital shaker (170 rpm); [substrate]: 10–100 mm; [NAD^+^]: 1 mm; [LysEDH]: 45 μm; [Cb‐FDH]: 16 μm; HCOONa (100 mm).

All of the alcohol yields reported in Table 3 were obtained using 20 mm of aldehyde substrate. Therefore, we determined the influence of substrate concentration on the reaction conversions. Benzaldehyde (**1 b**) was selected as the test substrate at concentrations ranging from 10 to 100 mm, whereas wild‐type LysEDH (45 μm) was selected as the promiscuous ADH enzyme because it gave the highest conversion in the reduction of **1 b** to **1 a**. The reactions were run again at 30 °C for 24 h in a KPi buffer (pH 7, 100 mm) supplemented with HCOONa (100 mm), NAD^+^ (1 mm) and Cb‐FDH (16 μm). The conversion of **1 b** to **1 a** was 90 % at 100 mm substrate concentration, which resulted in a 76 % analytical yield (Figure 3). As mentioned, the apparent loss of mass balance is due to the volatility of **1 b**. Notably, a 10‐fold increase in **1 b** concentration did not significantly affect the reaction yield, which ranged from 95 % (at 10 mm of **1 b**) to 76 % (at 100 mm of **1 b**).

Michaelis–Menten kinetic parameters were determined in our previous study for the reductive amination of **1 b** to **1 c** catalyzed by LE‐AmDH‐v1 (*k*_app_=43.6±1.7 min^−1^; *K*_M app_=4.9±0.5 mm, measured at 60 °C).^[15k]^ However, Michaelis–Menten kinetics for the reduction of **1 b** to **1 a** could not be performed in this study because the alcohol **1 a** possesses high extinction coefficient at the same range of wavelengths that is needed for the quantitative monitoring of NADH depletion. However, *para*‐fluorobenzaldehyde (**2 b**) turned out to be the suitable substrate for our kinetic study. Because LE‐AmDH‐v1 was the enzyme that afforded high conversions for both the reduction of **2 b** to **2 a** (64 %, Table 3) and the reductive amination of **2 b** to **2 c** (90 %, Table 1), it was an excellent case for comparing carbonyl reduction with reductive amination activities. Herein, Michaelis–Menten kinetics conducted at 60 °C show that LE‐AmDH‐v1 acts preferentially as AmDH toward **1 b** (for reductive amination: *k*_app_/*K*_M app_ 6082 m ^−1^ min^−1^; for reduction to alcohol: *k*_app_/*K*_M app_ 272 m ^−1^ min^−1^; see Supporting Information section 8 and Table S10 for details). In fact, *k*_app_ value is greatly in favor of the reductive amination over the reduction to alcohol (22.0±0.9 min^−1^ vs. 0.15±0.01 min^−1^). In contrast, *K*_M app_ value is ∼4‐fold better for the reduction to alcohol than for the reductive amination (0.55±0.01 mm vs. 3.62±0.46 mm). These data shows that the relative levels of AmDH vs. ADH activity cannot be assessed based on only specific activity data (as reported in Supporting Information, Table S12), but *K*_M app_ has also an important contribution.

### One‐enzyme, dual‐activity (ADH‐AmDH) for alcohol amination

**Figure 4:**
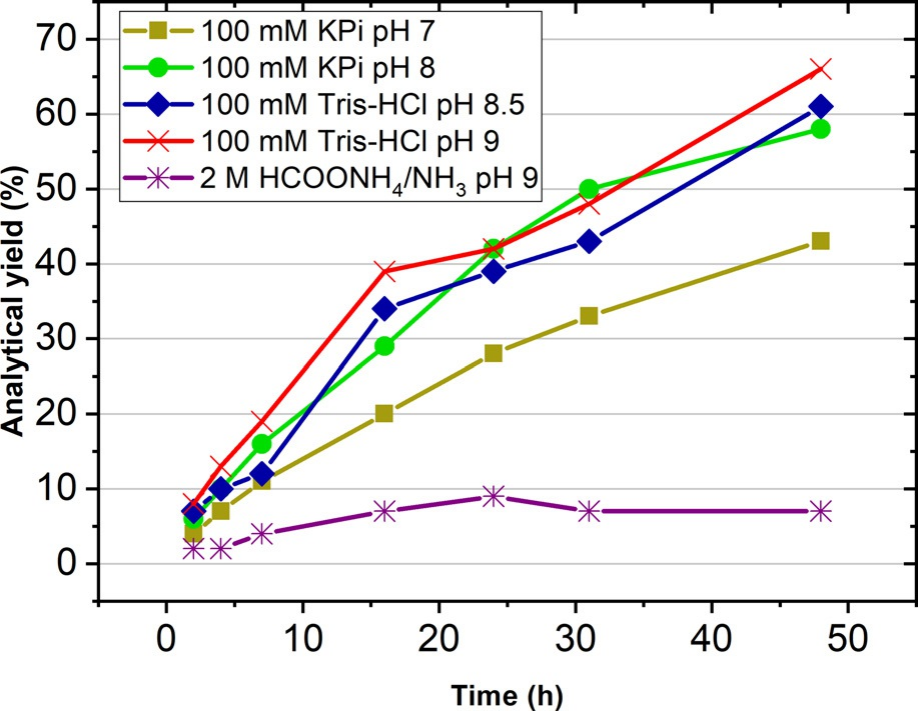
Time study for the oxidation of benzylic alcohol (**1 a**) to benzaldehyde (**1 b**) in different buffers at varied pH (7–9) catalyzed by LE‐AmDH‐v27. The progress of the analytical yield (%) was monitored over 2 days. Experimental conditions: 0.5 mL final volume in Eppendorf tubes; KPi and Tris‐HCl buffer are 100 mm; ammonium formate buffer is 2 m; *T*: 30 °C; agitation orbital shaker (170 rpm); [substrate]: 10 mm; [NAD^+^]: 1 mm; [LE‐AmDH‐v27]: 90 μm; [NOx]: 10 μm.

As the LE‐AmDH variants exhibited both native AmDH activity and promiscuous ADH activity, we envisioned that this dual‐activity could be harnessed for the amination of primary alcohols via a one‐enzyme oxidative‐reductive cascade. Such a redox self‐sufficient process is often referred as “hydrogen‐borrowing” or, more properly, “hydride‐borrowing” because the hydride abstracted from the first oxidation of the alcohol substrate to the ketone intermediate is delivered back in the second reductive amination step. This process was first reported by our group through the combination of an ADH with an AmDH.^[25]^ Therefore, herein, we studied whether a single dehydrogenase enzyme could enable the same two‐step redox process. However, the optimal reaction conditions for the oxidation of an alcohol to a carbonyl compound are different from the optimal conditions for reverse reduction. In particular, basic pH values appear to favor the oxidation reaction, whereas pH close to or at neutrality favors the reduction reaction.^[26]^ Therefore, we performed a study on the oxidation of benzylic alcohol (**1 a**) to benzaldehyde (**1 b**) in different buffers, at different pH levels, and using LE‐AmDH‐v27 due to its high catalytic activity. Figure 4 depicts the progress of the analytical yield over time for the oxidation reaction (see Supporting Information section 5.5. and Table S5 for details) in which a water‐forming nicotinamide adenine dinucleotide oxidase (NOx) was used as NAD^+^‐recycling enzyme.^[27]^

Figure 4 shows that higher pH values favor the alcohol oxidation reactions, which is in agreement with the literature. In fact, the highest analytical yield of 66 % after 24 h reaction time was obtained in Tris‐HCl (pH 9, 100 mm). However, the analytical yield was only 7 % for the oxidation performed for 24 h, at pH 9 and in a 2 m ammonium formate buffer. We infer that the lower aldehyde formation observed in the ammonium formate buffer compared with the other buffers might be due to a negative effect on the NOx cofactor‐recycling enzyme.

**Figure 5:**
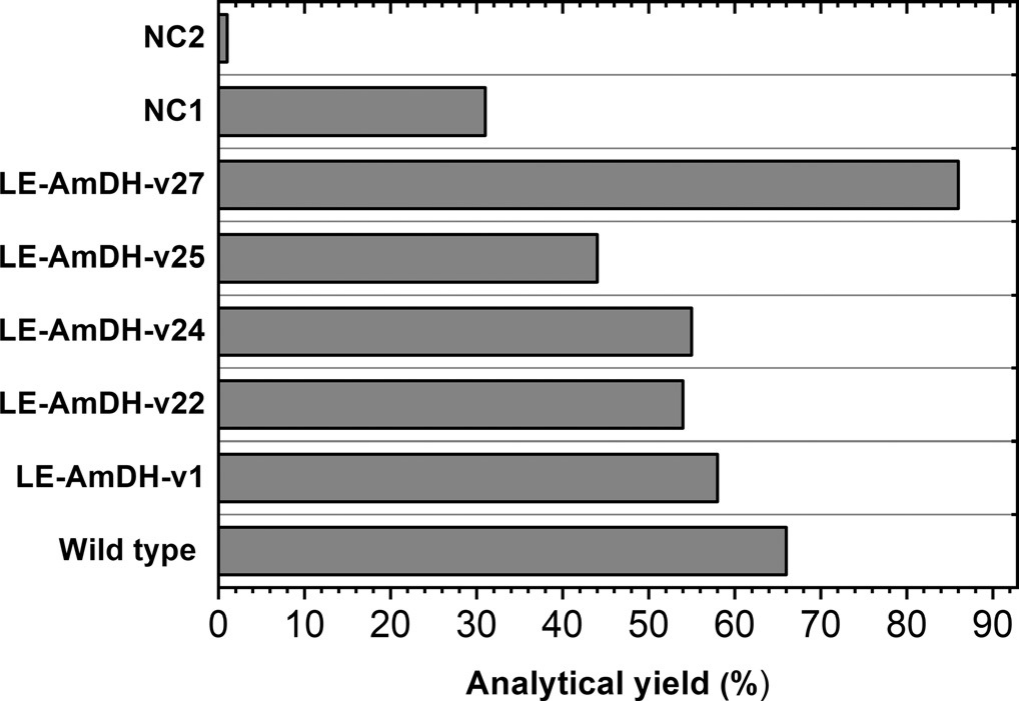
Oxidation of benzylic alcohol (**1 a**) to benzaldehyde (**1 b**) catalyzed by wild‐type LysEDH and the five LE‐AmDH variants. The analytical yields (%) depicted here are the average values obtained from two independent experiments. NC 1: reaction without LysEDH or LE‐AmDH variant, NC 2: reaction without any enzyme addition (LysEDH or LE‐AmDH, and NOx). Experimental conditions: 0.5 mL final volume in Eppendorf tubes; buffer: Tris‐HCl (100 mm, pH 9); *T*: 30 °C; agitation on orbital shaker (170 rpm); reaction time: 48 h; [substrate **1 a**]: 10 mm; [NAD^+^]: 1 mm; [LE‐AmDHs]: 90 μm; [NOx]: 10 μm.

The oxidation of **1 a** to 1**b** was tested with all of the variants in the best performing buffer, namely Tris‐HCl buffer (pH 9, 100 mm). Figure 5 shows that all of the LE‐AmDH variants were capable of producing **1 b** in moderate to high yields (44–86 %; see Supporting Information section 5.5 and Table S6 for details). However, the negative control reactions in which only NOx was present as the enzyme (NC 1) afforded **1 b** in a 31 % yield; therefore, the oxidation of **1 a** to **1 b** (Figure 5) is partly due to an alcohol oxidase activity of NOx. Nevertheless, the highest yield for the oxidation reaction was achieved using LE‐AmDH‐v27 as the biocatalyst (86 %), which possesses the beneficial F173S substitution for ADH activity along with the additional T240A and Y238A mutations. However, the 66 % yield given by wild‐type LysEDH is significantly higher than that obtained from the negative control reaction (NC1). Considering both the oxidation (Figure 5) and the reduction (Table 3) experiments, LE‐AmDH‐v27 was the variant that exhibited the overall highest alcohol dehydrogenase activity. In fact, LE‐AmDH‐v27 was also the variant possessing the highest specific activity for carbonyl reduction to alcohol when assayed for **2 b** as substrate (see Supporting Information section 9 and Table S12 for details).

**Scheme 2:**
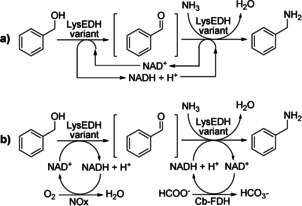
Exploiting the dual ADH/AmDH activity of LysEDH variants for the one‐pot conversion of benzylic alcohol (**1 a**) to benzylamine (**1 c**): a) one‐enzyme hydride‐borrowing alcohol amination; b) biocatalytic network using a single LysEDH variant combined with NOx and Cb‐FDH.

In the following step, we attempted the direct conversion of benzylic alcohol (**1 a**) to benzylamine (**1 c**) using only one enzyme in an oxidative‐reductive amination cascade (Scheme 2 a).

**Figure 6:**
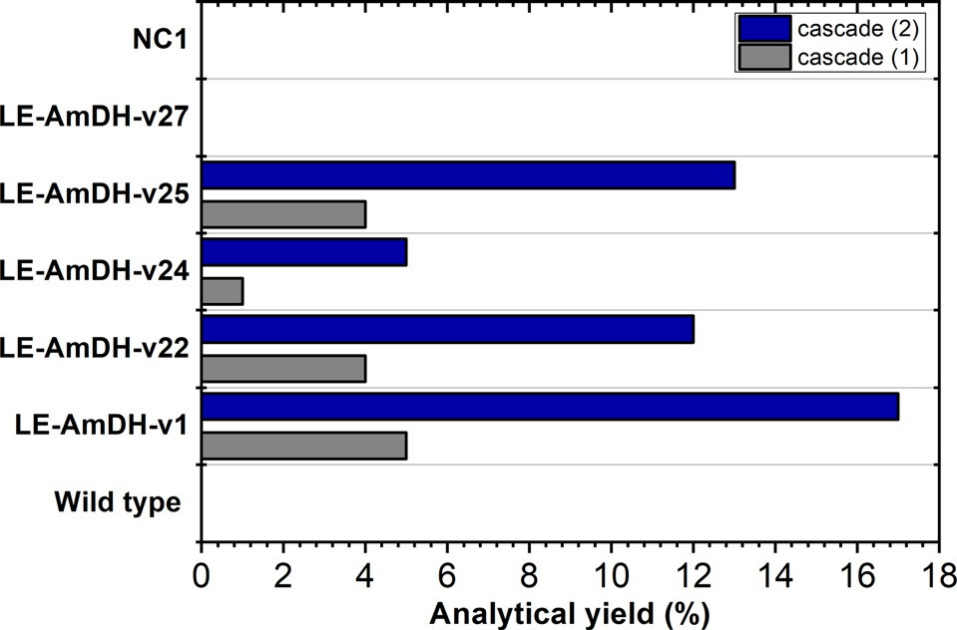
Amination of benzyl alcohol (**1 a**) to benzylamine (**1 c**) using a single dual‐activity ADH/AmDH variant for both oxidation and reductive amination steps. wild‐type LysEDH and all of the LE‐AmDH variants were tested for this transformation. Cascade 1 (one‐enzyme cascade) buffer: Tris‐HCl (100 mm) supplemented with NH_4_OH (1 m) final pH 9; *T*: 30 °C; reaction time: 48 h; agitation orbital shaker (170 rpm); [**1 a**]: 10 mm; [NAD^+^]: 1 mm; [LysEDH or LE‐AmDH variant]: 90 μm. Cascade 2 (one LE‐AmDH variant plus NOx and FDH for orthogonal NAD cofactor recycling) buffer: Tris‐HCl (100 mm) supplemented with NH_4_OH (1 m), final pH 9; *T*: 30 °C; reaction time: 48 h; agitation orbital shaker (170 rpm); [**1 a**]: 10 mm; [NAD^+^]: 1 mm; [NOx]: 10 μm; [LysEDH or LE‐AmDH variant]: 90 μm; added after 24 hours: [HCOONa]: 100 mm and [FDH]: 16 μm.

In this context, the LE‐AmDH variant assumes a bi‐functional role, simultaneously acting as ADH and AmDH. The catalytic amount of NAD^+^/NADH cofactor was internally regenerated due to the inherent redox‐neutrality of the process. However, the previous results demonstrated that the most active LE‐AmDH variants for the oxidation of **1 a** to **1 b** have a modest activity for the reductive amination of **1 b** to **1 c** (and vice versa). Additionally, the formed benzaldehyde (**1 b**) intermediate can undergo both reductive amination to **1 c** as well as reduction back to **1 a**. With the aim of finding a compromise between the alcohol oxidation and ketone reductive amination steps, we performed a number of experiments (see Supporting Information section 5.6). Under optimized conditions, the hydride‐borrowing alcohol amination of **1 a** (10 mm) was conducted with all of the LE‐AmDH variants (90 μm) in Tris‐HCl (100 mm, pH 9) at 1 m NH_4_OH and for 48 h. Figure 6 (cascade 1) shows that a 4–5 % maximum yield of **1 c** was obtained using LE‐AmDH‐v1, ‐v22 and ‐v25, whereas conversion with LE‐AmDH‐v1 was just above the detection limit. Varying the NAD^+^ concentration (1, 5 and 10 mm) as well as the substrate concentration (10, 50 and 100 mm, respectively) while keeping a constant NAD^+^ vs. substrate molar ratio (1: 10) resulted in increased production of **1 c** (0.4, 2.1 and 2.5 mm, respectively).

Finally, the bi‐functional dehydrogenases were tested in a two‐step amination of **1 a** to **1 c** in a similar manner as described in our previous publication (Scheme 2 b).^[13]^ However, in this case, although the oxidative and the reductive steps were still performed in one‐pot, they were separated in time because both steps require NAD as hydride transfer agent (Figure 6, cascade 2). In practice, the reaction was initiated by adding NAD^+^ (1 mm), NOx (10 μm), LE‐AmDH variant (90 μm) and **1 a** (20 mm) in Tris‐HCl (0.5 mL, pH 9, 100 mm, NH_4_OH 1 m) at 30 °C for 24 h. During this time, **1 a** was converted into **1 b** by the LE‐AmDH variant while NAD^+^ was recycled by NOx at the expense of dioxygen. After the oxidative step, other 0.5 mL of the reaction buffer containing HCOONa (200 mm) and Cb‐FDH (16 μm) were added so that the same LE‐AmDH variant could catalyze the reductive amination of **1 b** to **1 c**; NADH was recycled by Cb‐FDH at the expense of HCOONa. Therefore, the final concentrations in this second step were LE‐AmDH variant (45 μm), Cb‐FDH (8 μm) and HCOONa (100 mm). Under these reaction conditions, LE‐AmDH‐v1 produced the highest analytical yield of **1 c** (17 %), followed by LE‐AmDH‐v25, ‐v22 and ‐v24 with 13 %, 12 % and 5 %, respectively. Furthermore, the analytical yield of **1 c** could be increased up to 34 % and 32 % using LE‐AmDH‐v1 and ‐v25, respectively, by applying a slight modification of the procedure. In practice, the oxidative step of the cascade was performed for 24 h in Tris‐HCl buffer (0.5 mL, pH 9, 100 mm) as previously (**1 a** 20 mm, LE‐AmDH variant 90 μm) but in the absence of ammonia. Then, the ammonia solution (0.5 mL, 1 m, pH 9) containing HCOONa (200 mm) and Cb‐FDH (16 μm) was added to initiate the reductive amination step, which was run for additional 24 h. Therefore, the final concentrations in this second step were again LE‐AmDH variant (45 μm), Cb‐FDH (8 μm) and HCOONa (100 mm). This increase of the yield for **1 c** is in agreement with the data reported in Figure 4 (purple line) for the oxidation of **1 a** to **1 b**, which was indeed impeded by the presence of ammonia in solution. As previously described, we attribute this negative behavior to the poor stability of NOx at high concentration of ammonia/ammonium species.

Figure 6 also shows that neither wild‐type LysEDH nor LE‐AmDH‐v27 could produce any detectable amount of amine **1 c**; however, both enzymes could oxidize **1 a** to **1 b** in an efficient manner, and LE‐AmDH‐v27 was in fact the best variant for this transformation (Figure 5). Therefore, wild‐type LysEDH and LE‐AmDH‐v27 must be incapable of converting **1 a** into **1 c** due to a limitation in the reductive amination step. Notably, LysEDH and LE‐AmDH‐v27 were the only two enzymes that produced alcohol **1 a** as the by‐product (23 % and 22 %, respectively) along with the amine **1 c** (17 % and 52 %, respectively) in the reductive amination experiments in an ammonium formate buffer (Table 1). Therefore, we conclude that LysEDH and LE‐AmDH‐v27 cannot convert **1 a** into **1 c** in the alcohol amination cascades because the generated intermediate **1 b** is preferentially reduced back to **1 a** rather than aminated to **1 c**. In contrast, the other LE‐AmDH variants (v1, v22, v24 and v25) fully behaved as amine dehydrogenases when the aldehyde **1 b** was reduced in the ammonium buffer, thereby yielding **1 c** as the sole product (Table 1, 80‐>99 %). In summary, this chemoselective dual‐activity of LE‐AmDH‐v1, ‐v22, ‐v24 and ‐v25 in an ammonium buffer (i.e., ADH activity for the oxidation of **1 a** to **1 b** and AmDH activity for the reduction of **1 b** to **1 c**) enables this unprecedented one‐dehydrogenase alcohol amination.

## Conclusions

In this study, we have explored and harnessed the catalytic promiscuity of l‐lysine‐(*ϵ*‐deaminating)‐dehydrogenase from *G. stearothermophilus* (LysEDH). Surprisingly, this wild‐type enzyme exhibited both amine dehydrogenase (AmDH) and alcohol dehydrogenase (ADH) activities toward benzaldehyde and *para*‐fluorobenzaldehyde. Starting from a first‐generation variant (LE‐AmDH‐v1) obtained from this scaffold, we created new variants possessing enhanced dual ADH/AmDH activity. Notably, the ADH and AmDH activities toward aldehydes could be tuned by altering the reaction conditions such that these dehydrogenases could behave either as an AmDH or as a primary ADH. LE‐AmDH‐v1 and ‐v25 exclusively behaved as AmDHs in an ammonium buffer and as ADHs in Tris‐buffer without ammonium species. More generally, the LE‐AmDH variants possessing the F173A mutation (v1, v22 and v25) favored AmDH activity toward aldehydes, whereas those possessing the F173S mutation (v24 and v27) and LysEDH exhibited preferential ADH activity. Therefore, LE‐AmDHs v1, v22 and v25 aminated substituted benzaldehydes **1 b–4 b** and **7 b** with maximum yields of 91‐ >99 % yields as well as substituted benzaldehydes **5 b** and **6 b** with 11 % and 13 % yields, respectively. On the other hand, LE‐AmDHs v24 and v27 reduced the same aldehydes **1 b–6 b** to the related alcohols **1 c–6 c** with maximum yields of 40‐ >99 %. Interestingly, wild‐type LysEDH gave the highest yield of 82 % for the reduction of benzaldehyde (**1 b**) to benzyl alcohol (**1 c**).

Furthermore, all of the LE‐AmDH variants (but not the wild‐type enzyme) could perform the reductive amination of some ketone substrates and yield the amine product with excellent enantiomeric excess (>99 %) in the large majority of cases. LE‐AmDHs v1, v22 and v25 were again the best aminating variants, which could convert structurally diverse ketones such as acetophenone, 1‐indanone, propiophenone, 1‐tetralone, 4‐chromanone and other aliphatic ketones (**11 b–15 b,18 b–21 b**) into enantiopure amines with maximum yields of 76–99 %. However, none of the enzymes could practically reduce the same ketone substrates to any of the related secondary alcohols. This observation suggests that in contrast to the possible productive binding of ketimines as “AmDH‐type intermediates”, ketones as “ADH‐type substrates” cannot bind in any reactive conformation in the enzyme's active site. Nonetheless, both aldehydes as “ADH‐type substrates” and aldimines as “AmDH‐type intermediates” were converted equally well, which is probably because aldehydes possess a higher conformational flexibility than ketones when they are bound in the enzyme's active site. Therefore, at least a productive binding mode was attained for benzaldehydes reductions to alcohols. In principle, the promiscuous ADH activity of the LE‐AmDH variants could be extended to ketones by applying further enzyme engineering.

Finally, ADH activity was also tested with all of the LE‐AmDH variants for the oxidation of benzylic alcohol to benzaldehyde in the presence or absence of ammonia/ammonium species. LE‐AmDH‐v27 turned out to be the best variant (86 % yield). Therefore, this unprecedented dual ADH/AmDH activity was applied for the first example of one‐enzyme hydride‐borrowing alcohol amination, which yielded 5 % of benzylamine product using LE‐AmDH‐v1. Notably, LE‐AmDH‐v1 and ‐v25 could catalyze benzyl alcohol amination with 34 % and 32 % yields of benzylamine, respectively, by separating the oxidative and the reductive steps in time. To the best of our knowledge, LE‐AmDH‐v1, ‐v22 and ‐v25 represent the first examples of oxidoreductases that have been applied for the one‐enzyme conversion of an alcohol into an amine, thereby exhibiting an “alcohol aminase” activity.

## Experimental Section

For general information, material, enzymes preparation, details on biocatalytic reactions, analytics and chromatograms, see the Supporting Information.

### General procedure for the reductive amination of aldehydes

The biocatalytic reaction was carried out in an ammonium formate buffer (1 mL, 2 m, pH 8.5) by adding NAD^+^ (1 mm), Cb‐FDH (16 μm), LE‐AmDH (45 μm) and aldehyde (20 mm) in consecutive order. The reaction was incubated at 30 °C in an orbital shaker (170 rpm) for 24 h. Next, the reaction was acidified with formic acid (20 μL, until pH<4) and the organic compounds were extracted with EtOAc (2×500 μL EtOAc, containing internal standard). The aqueous layer was basified with KOH (300 μL, until pH>12) and the organic compounds were extracted (2×500 μL EtOAc, containing internal standard). The acidic and basic extracts were dried with MgSO_4_ and analyzed separately with GC‐FID. For details, see Supporting Information, section 5.

### General procedure for the reductive amination of ketones

The biocatalytic reaction was carried out in an ammonium formate buffer (0.5 mL, 2 m, pH 8.5) by adding NAD^+^ (1 mm), Cb‐FDH (16 μm), LE‐AmDH (90 μm) and ketone (10 mm). The reaction was incubated at 50 °C in an orbital shaker (170 rpm) for 48 h. Then the reaction was basified with KOH (100 μL, 10 m) and extracted with EtOAc (1×600 μL). The organic phase was dried with MgSO_4_ and analyzed by GC‐FID. For details, see Supporting Information, section 5.

### General procedure for the reduction of ketones and aldehydes to alcohols

The biocatalytic reaction was carried out in a potassium phosphate buffer (1 mL, 100 mm, pH 7) supplemented with sodium formate (100 mm) by adding NAD^+^ (1 mm), Cb‐FDH (16 μm), LE‐AmDH (45 μm) and aldehyde or ketone (20 mm) in consecutive order. The reaction was incubated at 30 °C in an orbital shaker (170 rpm) for 24 h. Then the reaction was extracted with EtOAc (2×500 μL, containing internal standard). The combined organic phase was dried with MgSO_4_ and analyzed by GC‐FID. For details, see Supporting Information, section 5.

### One‐enzyme conversion of benzylic alcohol (1 a) to benzylamine (1 c)

The biocatalytic reaction was carried out in a Tris‐HCl buffer (1 mL, 100 mm) supplemented with NH_4_OH (1 m) at a final pH value of 9 and by adding NAD^+^ (1 mm), LE‐AmDH (90 μm) and **1 a** (10 mm). The reaction was incubated at 30 °C in an orbital shaker (170 rpm) for 48 h. Then the reaction was basified with KOH (200 μL, 10 m) and extracted with EtOAc (2×500 μL, containing internal standard). The combined organic phase was dried with MgSO_4_ and analyzed by GC‐FID. For details, see Supporting Information, section 5.

## Conflict of interest

The authors declare no conflict of interest.

## Supporting information

As a service to our authors and readers, this journal provides supporting information supplied by the authors. Such materials are peer reviewed and may be re‐organized for online delivery, but are not copy‐edited or typeset. Technical support issues arising from supporting information (other than missing files) should be addressed to the authors.

Supplementary
