# Supplementary material for: Generation of Oxidoreductases with Dual Alcohol Dehydrogenase and Amine Dehydrogenase Activity
Source: Chemistry. 2020 Dec 9;27(10):3315–25. doi: 10.1002/chem.202003140 (PMC7898336; doi:10.1002/chem.202003140)
Supplement: Supplementary file 1 — Supplementary [file CHEM-27-3315-s001.pdf]

Chemistry–A European Journal

Supporting Information

Generation of Oxidoreductases with Dual Alcohol Dehydrogenase and Amine Dehydrogenase Activity

Vasilis Tseliou+, Don Schilder+, Marcelo F. Masman, Tanja Knaus, and Francesco G. Mutti*[a]

Table of Contents

1. List of abbreviations **2**

2. General information **3**

3. Calculation of response factors between alcohols, aldehydes or ketones and amines **4**

4. Enzyme preparation **9**

5. Biocatalytic transformations **10**

5.1 General procedure for the reductive amination of aldehydes and ketones. *10*

5.2 Determination of the pH optimum for the reduction of 1b to 1a by LE-AmDH-v27. *10*

5.3 Progress for the reduction of 1b to 1a in different types of buffers. *11*

5.4 General procedure for the reduction of ketones and aldehydes to alcohols *12*

5.5 Investigation on the oxidation of 1a to 1b. *12*

5.6 Investigation of the direct conversion of 1a to 1c. *14*

6. Thermodynamic calculations **16**

Reaction 1 (carbonyl reduction of benzaldehyde 1b) *16*

Reaction 2 (reductive amination of benzaldehyde 1b) *17*

Reaction 3 (carbonyl reduction of acetophenone 11b) *17*

Reaction 4 (reductive amination of acetophenone 11b) *18*

7. Analytical methods **19**

8. Steady-state kinetics for LE-AmDH-v1, LE-AmDH-v22 and LE-AmDH-v25 **21**

Determination of the saturation concentration of NADH for Ch1-AmDH and LE-AmDH-v1 *21*

Steady-state kinetics *21*

9. Determination of enzymatic specific activities **25**

10. Chromatograms **26**

10.1 GC-FID chromatograms of reductive aminations *26*

10.2 GC-FID chromatograms for determination of the absolute configuration of the amines *47*

10.3 GC-FID chromatograms of reduction of aldehydes and ketones to alcohols *55*

10.4 GC-FID chromatograms of oxidation of 1b to benzyl-alcohol *76*

10.5 GC-FID chromatograms of cascade reactions *77*

11. References **78**

1. List of abbreviations

LE-AmDH-v	indicates a variant originated from the NADH-dependent ε-deaminating L-lysine dehydrogenase from *Geobacillus stearothermophilus*[1]
NOx	NAD- and flavin-dependent oxidase from *Streptococcus mutans*[2]
ee	enantiomeric excess
ADH	alcohol dehydrogenase
AF	ammonium formate
A_{IS}	GC peak area of internal standard
AmDH	amine dehydrogenase
A_x	GC peak area of compound **x**
Cb-FDH	formate dehydrogenase from *Candida boidinii*
Ch1-AmDH	chimeric amine dehydrogenase[3]
DMSO	dimethyl sulfoxide
DMAP	4-dimethylaminopyridine
enz	enzyme reference
EtOAc	ethyl acetate
ext	extraction reference
inc	incubation reference
inj	injection reference
IS	internal standard
KPi	potassium phosphate
LB	lysogenic broth
MW	molecular weight
NAD^+	nicotinamide adenine dinucleotide (oxidative form)
$NADP^+$	nicotinamide adenine dinucleotide 3'-phosphate (oxidative form)
n.d.	not determined
n.a.	not applicable
n.m.	not measured
Ni-NTA	nickel-nitrilotriacetic acid
ONC	overnight cultures
rac	racemic
RF	response factor
DCM	dichloromethane

2. General information

Unless otherwise stated, substrates were added as stock solutions in DMSO (1 M). Nicotinamide adenine dinucleotide (NAD^+) was purchased from Melford Biolaboratories (Chelsworth, Ipswich, UK). Column chromatography was carried out with Ni^{2+} affinity columns (HisTrap FF, 5 mL) from GE Healthcare Bio-Sciences (Munich, Germany).

In all of the biotransformations, toluene was used as the internal standard (IS).

3. Calculation of response factors between alcohols, aldehydes or ketones, and amines

Toluene (20 mM final concentration) served as internal standard (IS). Measurements were performed in triplicate. Alcohols and aldehydes or ketones were incubated in KPi buffer, whereas amines were incubated in ammonium formate buffer.

(i) Injection samples (***inj***): 20 mM of reference compounds (**Fig. S1**) were pipetted into EtOAc containing 20 mM IS (final volume 1 mL, in 2 mL Eppendorf tubes); the area of IS and compound were determined by GC-FID and served as the samples in which the RF was considered 100%.

(ii) Extraction samples (***ext***, extractability of organic compounds): 20 mM of the reference compounds were pipetted into the reaction buffer (final volume 1 mL, in 2 mL Eppendorf tubes) and mixed. After basifying with KOH (200 µL, 10 M), the compounds were extracted with EtOAc supplemented with IS (2 x 500 µL).

(iii) Incubation samples (***inc***, volatility of organic compounds): 20 mM of the reference compounds were pipetted into the reaction buffer (final volume 1 mL, in 2 mL Eppendorf tubes) and incubated at 30 °C in orbital shakers (170 rpm) for 24 h. After basifying with KOH (200 µL, 10 M), the compounds were extracted with EtOAc supplemented with IS (2 x 500 µL).

(iv) Enzyme samples (***enz***, to study possible interaction between organic compounds and the enzyme): 20 mM of the reference compounds were pipetted into the reaction buffer (final volume 1 mL, in 2 mL Eppendorf tubes) that contained 45 µM of Ch1-AmDH and incubated at 30 °C in orbital shakers (170 rpm) for 24 h. After basifying with KOH (200 µL, 10 M), the compounds were extracted with EtOAc supplemented with IS (2 x 500 µL).

The response factors were calculated from the ratio: peak area compound/peak area IS. Notably, only the compounds for which the response factor was >65% compared with the injection sample (***inj***) were selected for biocatalytic reactions.

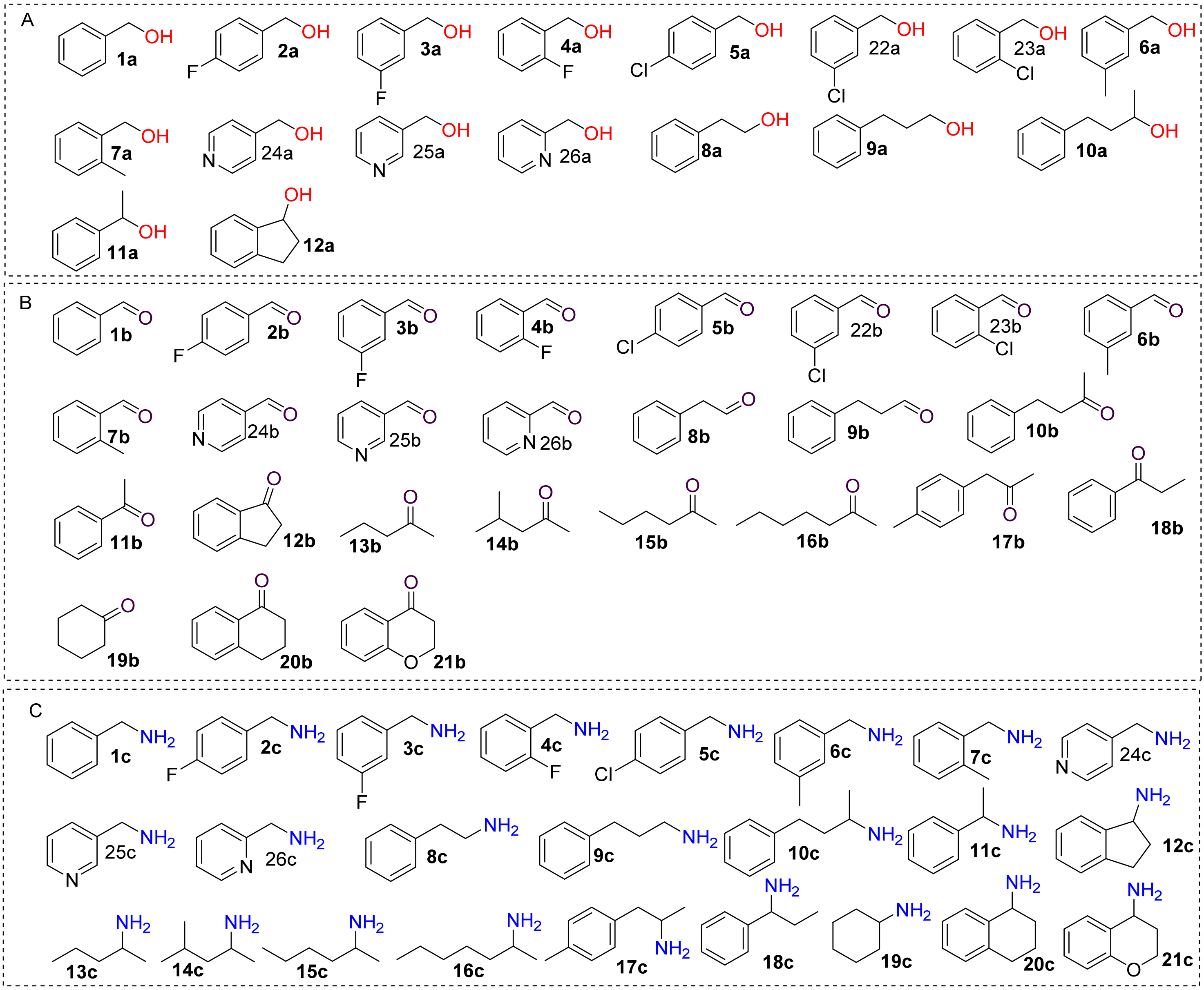

Fig. S1. Complete list of compounds used in this study. Compounds that were used in biocatalytic reactions have their identification number highlighted in bold

Table S1 : Response factors of compounds **1a-7a**, **1b-7b**, **1c-7c**, **12a**, **13a**, **12b**, **13b**, **12c**, **13c**, **15a-22a**, **15b-22b** and **15c-22c** measured with GC-FID. Four different samples were used: directly injected in EtOAc (***inj***), extracted from buffer (***ext***), extracted from buffer after 24 h incubation time (***inc***), and extracted from buffer after 24h incubation in the presence of Ch1-AmDH (***enz***).

**Compound**	**Response factor (RF)**			
	inj	***ext***	***inc***	***enz***
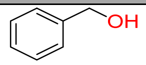	1.18 (100%)	1.12 (95%)	1.09 (92%)	1.11 (94%)
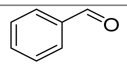	1.01 (100%)	0.97 (96%)	0.86 (86%)	0.88 (88%)
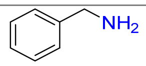	0.88 (100%)	0.75 (85%)	0.77 (88%)	0.79 (90%)
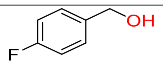	1.16 (100%)	1.12 (96%)	1.12 (96%)	1.12 (97%)
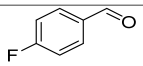	1.04 (100%)	0.98 (94%)	0.88 (85%)	0.91 (87%)
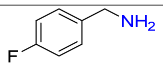	1.09 (100%)	1.14 (104%)	1.12 (102%)	1.14 (104%)
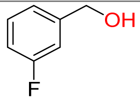	1.01 (100%)	1.03 (103%)	1.05 (104%)	1.05 (104%)
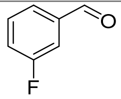	0.98 (100%)	0.95 (97%)	0.83 (85%)	0.83 (85%)
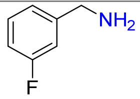	1.10 (100%)	1.10 (99%)	1.06 (96%)	1.08 (98%)
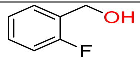	1.11 (100%)	1.09 (99%)	1.09 (98%)	1.14 (103%)
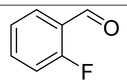	0.93 (100%)	0.89 (97%)	0.76 (82%)	0.75 (81%)
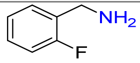	1.05 (100%)	1.02 (97%)	1.01 (96%)	1.01 (96%)
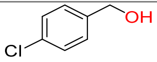	1.09 (100%)	1.12 (103%)	1.16 (107%)	1.15 (106%)
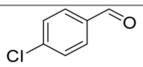	1.04 (100%)	1.04 (100%)	0.79 (76%)	0.76 (73%)
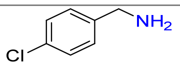	1.17 (100%)	1.18 (100%)	1.13 (97%)	1.16 (99%)

Compound	Response factor (RF)			
	inj	*ext*	*inc*	*enz*
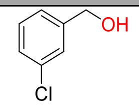	0.98 (100%)	0.97 (98%)	0.95 (96%)	0.98 (99%)
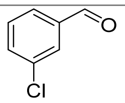	0.54 (100%)	0.52 (98%)	0.33 (62%)	0.34 (63%)
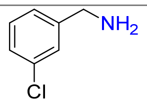	n.d.	n.d.	n.d.	n.d.
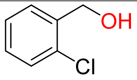	1.14 (100%)	1.19 (104%)	1.18 (103%)	1.18 (103%)
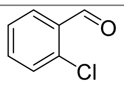	1.01 (100%)	0.97 (95%)	0.53 (53%)	0.58 (57%)
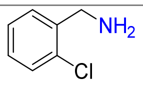	n.d.	n.d.	n.d.	n.d.
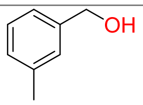	1.32 (100%)	1.34 (102%)	1.34 (102%)	1.37 (104%)
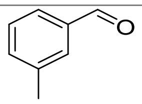	1.19 (100%)	1.15 (96%)	0.78 (66%)	0.81 (68%)
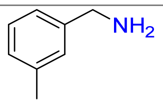	1.19 (100%)	1.24 (104%)	1.24 (104%)	1.27 (106%)
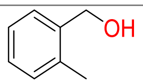	1.15 (100%)	1.18 (103%)	1.11 (97%)	1.11 (97%)
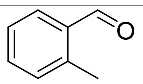	1.08 (100%)	1.04 (96%)	0.72 (66%)	0.76 (70%)
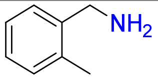	1.32 (100%)	1.29 (98%)	1.24 (94%)	1.26 (95%)
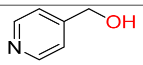	0.79 (100%)	0.48 (61%)	0.49 (62%)	0.48 (61%)
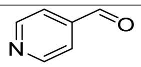	0.82 (100%)	0.53 (64%)	0.52 (63%)	0.52 (63%)
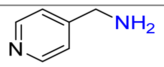	0.91 (100%)	0.07 (8%)	0.07 (8%)	0.07 (8%)

Compound	Response factor (RF)			
	inj	***ext***	***inc***	***enz***
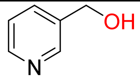	n.d.	n.d.	n.d.	n.d.
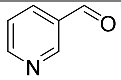	0.70 (100%)	0.54 (78%)	0.55 (79%)	0.56 (80%)
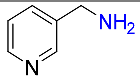	0.90 (100%)	0.07 (8%)	0.07 (8%)	0.05 (6%)
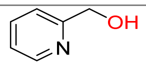	n.d.	n.d.	n.d.	n.d.
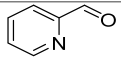	0.76 (100%)	0.65 (85%)	0.67 (88%)	0.67 (88%)
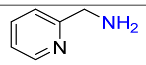	0.85 (100%)	0.09 (10%)	0.09 (10%)	0.09 (10%)
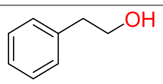	1.09 (100%)	0.91 (84%)	1.01 (93%)	1.01 (93%)
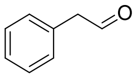	0.99 (100%)	0.85 (86%)	0.72 (73%)	0.46 (46%)
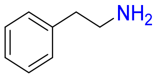	1.28 (100%)	1.06 (83%)	1.04 (81%)	1.09 (85%)
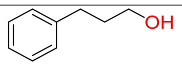	1.15 (100%)	1.04 (90%)	1.10 (95%)	1.11 (96%)
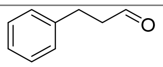	1.06 (100%)	0.96 (90%)	0.96 (90%)	0.73 (69%)
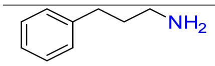	1.44 (100%)	1.47 (102%)	1.48 (103%)	1.47 (102%)

Experimental conditions: 1 mL final volume in Eppendorf tubes; buffer: 2 M ammonium formate pH 8.2 or 100 mM KPi pH 7; T = 30 ºC; incubation time = 24 h; agitation with orbital shaker (170 rpm); [compound] = 20 mM; [Ch1-AmDH] = 45 µM.
n.d: Not determined

4. Enzyme preparation

LE-AmDH-v1,[1] Ch1-AmDH,[3b] Cb-FDH[3c] and NOx[4] were expressed and purified as described previously.

The LysEDH variants (i.e., LE-AmDH-v) were obtained by site directed mutagenesis using the QuickChange II Site-Directed Mutagenesis Kit (Agilent Technologies) according to the protocol of the Supplier using LE-AmDH-v1 DNA as template. The primer can be found in **Table S2**. After verifying positive clones by sequencing, expression and purification was done as for the v1 variant.

Table S2: Primer used for site directed mutagenesis.

Enzyme	Mutations	Template DNA	mg_{enzyme} $L_{culture}^{-1}$		Primer
	Y238A	wild type		forw<br>rev	GAATCTGAAACGTCTGGAAGCCAAAACCATTCGTTATCGTGG<br>CCACGATAACGAATGGTTTTGGCTTCCAGACGTTTCAGATTC
	Y238A<br>T240A	wild type		forw<br>rev	CTGAAACGTCTGGAAGCCAAAGCCATTCGTTATCGTGGTCATG<br>CATGACCACGATAACGAATGGCTTTGGCTTCCAGACGTTTCAG
**LE-AmDH-v22**	F173A,<br>Y238A	Y238A	108	forw<br>rev	CTCTGGAATATAATCATGTTGCCAGCCTGGAAGGTCTGCTGG<br>CCAGCAGACCTTCCAGGCTGGCAACATGATTATATTCCAGAG
**LE-AmDH-v24**	F173S,<br>Y238A	Y238A	134	forw<br>rev	CTGGAATATAATCATGTTTCTAGCCTGGAAGGTCTGC<br>GCAGACCTTCCAGGCTAGAAACATGATTATATTCCAG
**LE-AmDH-v25**	F173A,<br>Y238A,<br>T240A	Y238A<br>T240A	82	forw<br>rev	CTCTGGAATATAATCATGTTGCCAGCCTGGAAGGTCTGCTGG<br>CCAGCAGACCTTCCAGGCTGGCAACATGATTATATTCCAGAG
**LE-AmDH-v27**	F173S,<br>Y238A,<br>T240A	Y238A<br>T240A	130	forw<br>rev	CTGGAATATAATCATGTTTCTAGCCTGGAAGGTCTGC<br>GCAGACCTTCCAGGCTAGAAACATGATTATATTCCAG

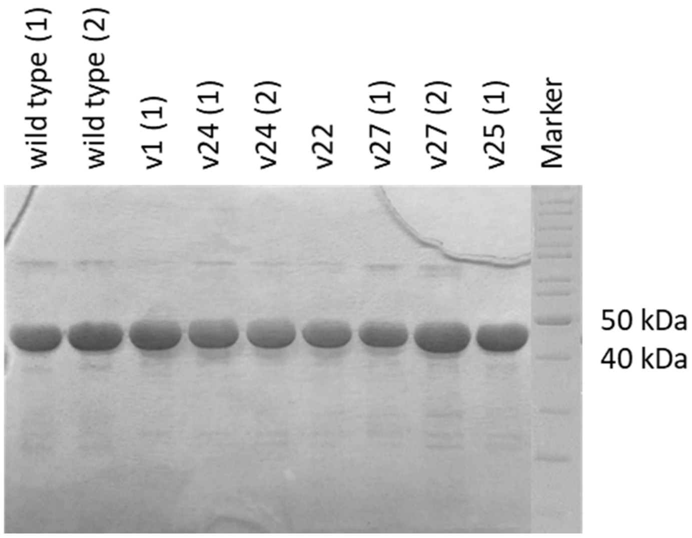

Fig. S2. SDS-PAGE of purified LE-AmDH variants and wild-type enzyme used in this study. Marker: Page Ruler Unstained Protein Ladder (Thermo Scientific)

5. Biocatalytic transformations

5.1 General procedure for the reductive amination of aldehydes and ketones.

Ammonium formate buffer (2 M; pH 8.5): $HCOONH_4$ (23.84 g, 0.378 mol) was dissolved in water (final volume = 200 mL). NH_4OH (2.72 mL of a concentrated 8.1 M solution) was added and the pH was adjusted to 8.5 with formic acid.
IS: toluene (20 mM final concentration).
Biocatalytic reactions of aldehydes (duplicate): total volume 1.0 mL (2 mL Eppendorf tubes) in ammonium formate buffer (2 M, pH 8.5). Reactions were started by adding NAD^+ (1 mM), Cb-FDH (16 µM), LE-AmDH (45 µM) and aldehyde (20 mM) in consecutive order.
Blank 1: no LE-AmDH
Blank 2: no LE-AmDH, no Cb-FDH
Reference samples: 20 mM compound in ammonium formate buffer (1 mL final volume).

Biocatalytic reactions were incubated at 30 °C in orbital shakers (170 rpm) for 24 hours. The reactions were acidified with formic acid (20 µL, pH < 4) and the organic compounds were extracted with EtOAc (2 x 500 µL EtOAc, containing the IS). Next, the water layer was basified with KOH (300 µL, 10M, pH >12) and the organic compounds were extracted (2 x 500 µL EtOAc, containing the IS). The acidic and basic extracts were dried with $MgSO_4$ and analyzed separately with GC-FID.
The reference samples were basified (100 µL KOH) and extracted (2 x 500 µL EtOAC, containing the IS).
The analytical yields of the obtained amines (or alcohols) were calculated by using their GC response factors.

Biocatalytic reactions of ketones: total volume 0.5 mL (1.5 mL Eppendorf tubes) in ammonium formate buffer (2 M, pH 8.5). NAD^+ (1 mM), Cb-FDH (16 µM), LysEDH (90 µM) and ketone (10 mM) were added and the reactions were run at 50 °C for 48 h at 170 rpm in orbital shakers. Then, the reaction mixture was basified (100 µL of 10 M KOH) and the extraction was performed with EtOAc (1 x 600 µL). The organic phases were dried with $MgSO_4$ and analyzed by GC-FID.

5.2 Determination of the pH optimum for the reduction of 1b to 1a catalyzed by LE-AmDH-v27.

Britton-Robinson universal buffer (BRB) was prepared using acetic acid (17.4 M), H_3PO_4 (14.7 M) and boric acid (solid MW: 61.83 g/mol) in the final concentration of 80 mM. 10 mL of this stock solution was titrated with 200 mM NaOH to give the desirable pH value. Then, dH_2O was added until the final volume of 20 mL resulted in 40 mM final concentration.

The biocatalytic reactions (final volume 500 µL) were performed using 45 µM of enzyme, 14 µM of FDH, 100 mM HCOONa, 1 mM of NAD^+ and 20 mM of **1b** and the reactions were incubated for different time points (10–80 min) at 30 °C using an orbital shaker (170 rpm). Extraction was performed using EtOAc (2x500 µL, supplemented with 10 mM toluene). The organic phases were combined, dried with $MgSO_4$ and analyzed by GC-FID. From the slopes that were obtained by plotting the mM product produced vs. time, the velocity of the reaction was calculated for every pH value (**Table S3**).

Table S3. pH optimum for the reduction of **1b** to **1a** using LE-AmDH-v27.

pH	Velocity ($\mu M\ min^{-1}$)
**6**	37.69
**7**	56.56
**7.5**	52.79
**8**	42.75
**8.5**	28.73
**9**	14.59

5.3 Progress for the reduction of 1b to 1a in different types of buffers.

For the biocatalytic reduction of **1b** to **1a**, several types of buffer were tested: (i) HEPES pH 7, 100 mM; (ii) Tris pH 7, 100 mM; (iii) MOPS pH 7, 100 mM; (iv) KPi pH 7, 100 mM and (v) NaPi pH 7, 100 mM.

Reactions (1 mL) were performed using 90 µM of the LE-AmDH variant, 16 µM of FDH, 20 mM of **1b**, 100 mM of HCOONa, and 1 mM of NAD^+. The biocatalytic reactions were run at 30 °C using an orbital shaker (170 rpm) and stopped at different time points (1–24 h). The organic compounds were extracted with EtOAc (2x500 µL, containing 10 mM IS), dried over $MgSO_4$ and analyzed by GC; the analytical yields (%) are reported in **Table S4** and **Fig. S3**. Both **Table S4** and **Fig. S3** report the progress of the analytical yield of the produced alcohol (expressed in mM) over the time. These results show that LE-AmDH-v27 has no apparent preference for a certain type of buffer because the reaction progress curves essentially overlapped and the reactions always resulted in ≥98.6% conversion after 24 h (corresponding to ≥18.8 mM of formed **1a**). Therefore, we decided to continue our study using the KPi buffer.

Table S4. Progress for the reduction (analytical yields %) of **1b** to **1a** using different types of buffers

Time (h)	100 mM HEPES pH 7	100 mM TRIS pH 7	100 mM MOPS pH 7	100 mM KPi pH7	100 mM NaH_2PO_4 pH 7
1	31	32	30	33	32
2	47	46	46	51	49
3	60	64	68	61	63
4	70	71	69	74	69
5	78	75	84	83	79
6	79	80	83	90	82
16	91	99	92	94	87

24	94	>99	96	94	95

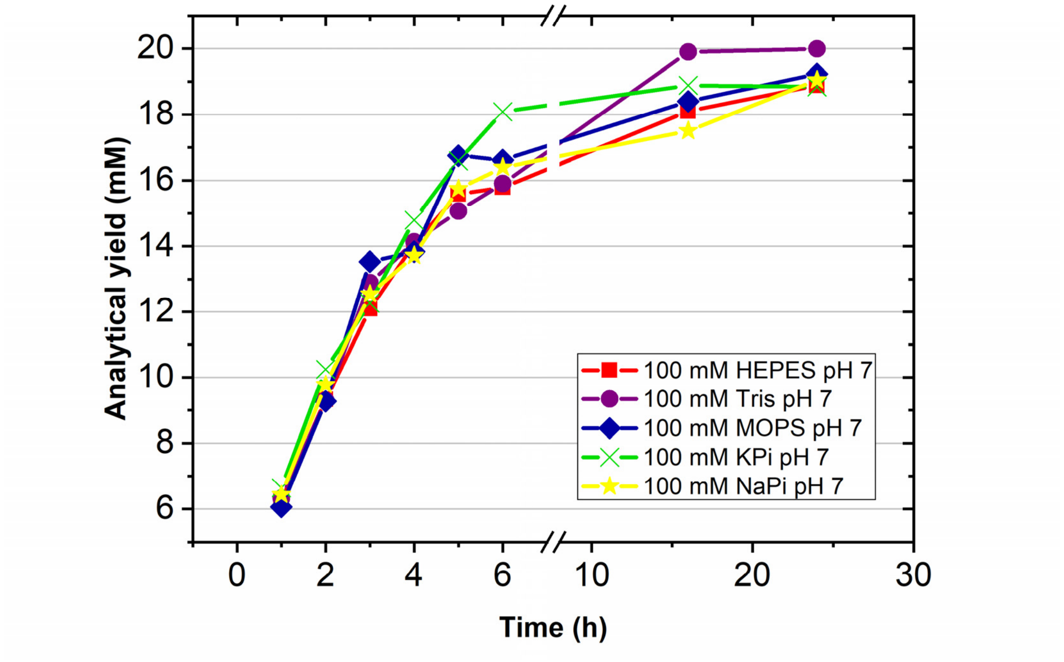

Fig. S3 . Progress of the reduction of **1b** to **1a** in different types of buffers at pH 7. Reaction conditions: **1b** (20 mM), NAD^+ (1 mM), HCOONa (100 mM), LE-AmDH-v27 (90 µM), Cb-FDH (16 µM), at 30 °C for 24 h and shaken at 170 rpm on an orbital shaker.

5.4 General procedure for the reduction of ketones and aldehydes to alcohols

KPi buffer (100 mM, pH 7.0) was prepared by adding K_2HPO_4 (3.74 g, 21.5 mmol) and KH_2PO_4 (2.52 g, 18.5 mmol) to distilled water (final volume = 0.4 L). The pH was adjusted to 7.0 by the addition of 10 M KOH. Then, a 1 M sodium formate (HCOONa) solution was made by dissolving 2.04 g HCOONa salt (30 mmol) in 30 mL KPi buffer. NAD^+ (27.3 mg, 40 mmol) was dissolved in 400 µL KPi buffer, thus giving a 100 mM NAD^+ stock solution. Reactions were conducted in a final volume of 1.0 mL (in 2 mL Eppendorf tubes). The experiment was performed in four replicates (substrate **1b**-**4b**) or in duplicate (substrate **5b**, **12b**, **13b**, **18b**-**22b**). At first, HCOONa (100 mM final concentration) was added to the buffer solution. Then, the biocatalytic transformations were initiated by the addition of NAD^+ (1 mM), Cb-FDH (16 µM), LE-AmDH (45 µM) and aldehyde (20 mM) in consecutive order. Two blank reactions were included. Blank 1 was devoid of LE-AmDH, while in blank 2 neither LE-AmDH nor Cb-FDH were present. All of the mixtures were incubated at 30 °C in orbital shakers (170 rpm). After 24 h incubation, the samples were extracted with EtOAc (2x500 µL, containing 20 mM IS). Analysis was carried out by GC-FID using a split ratio 1:20.

5.5 Investigation on the oxidation of 1a to 1b.

Five different buffers were tested: (i) KPi pH 7, 100 mM; (ii) KPi pH 8, 100 mM; (iii) Tris-HCl pH 8.5, 100 mM; (iv) Tris-HCl pH 9, 100 mM; (v) ammonium formate pH 9, 2 M. The biotransformations (single replicates) were performed in 0.5 mL buffer solution (in 1.5 mL Eppendorf tubes) in the presence of NAD^+ (1 mM),

NOx (10 µM), LE-AmDH-v27 (90 µM) and **1a** (10 mM). A blank sample was included, which did not contain LE-AmDH-v27. The reactions were incubated at 30 °C in orbital shakers (170 rpm) and stopped after different time points (2, 4, 7, 16, 24, 31 and 48 h). The organic compounds were extracted with EtOAc (2 x 500µL, containing 10 mM IS). Results are summarized in **Table S5**.

Table S5. Progress of the analytical yield (%) of **1b** over the time obtained in different buffers using LE-AmDH-v27

Time (h)	100 mM KPi pH 7	100 mM KPi pH 8	100 mM Tris-HCl pH 8.5	100 mM Tris-HCl pH 9	2 M $HCOONH_4/NH_3$ pH 9
2	4	6	7	8	2
4	7	10	10	13	2
7	11	16	12	19	4
16	20	29	34	39	7
24	28	42	39	42	9
31	33	50	43	48	7
48	43	58	61	66	7

Using the optimized reaction conditions (100 mM Tris-HCl pH 9, 48 h), all of the LE-AmDH variants were applied for the oxidation of **1a** (10 mM). Reactions were performed in 0.5 mL final volume containing LE-AmDH variant (90 µM), NAD^+ (1 mM) and NOx (10 µM). Reactions were run for 48 h at 30 °C in an orbital shaker (170 rpm). Two blank reactions were also prepared. Blank 1 was devoid of NOx and blank 2 was devoid of LE-AmDH and NOx. Results are summarized in **Table S6**.

Table S6. Oxidation of alcohol **1a** to aldehyde **1b** using six LE-AmDH variants. Conversions and yields depicted here are the average values obtained from two independent experiments.

Enzyme	Anal. Yield (%)
**WT**	66
**LE-AmDH-v1**	58
**LE-AmDH-v22**	54
**LE-AmDH-v24**	55
**LE-AmDH-v25**	44
**LE-AmDH-v27**	86
**Blank 1**	31
**Blank 2**	1

5.6 Investigation of the direct conversion of 1a to 1c.

Seven different buffers were tested (pH 9) for the synthesis of **1c** starting from **1a**: (i) Tris-HCl (100 mM) supplemented with NH_4OH (1 M); (ii) ammonium formate (1 M); (iii) ammonium acetate (1 M); (iv) ammonium phosphate (1 M); (v) ammonium chloride 1M (1 M); (vi) ammonium citrate (1 M); (vii) ammonium sulphate (1 M) .

The first buffer was prepared by adding NH_4OH to a Tris-HCl solution and adjusting the pH to 9 by the addition of concentrated HCl. For buffers ii-vii, a 2 M NH_4OH solution was made and the pH of this solution was adjusted to 9 by slowly adding the corresponding acid (e.g., formic acid). Then, the buffer was diluted with dH_2O to obtain the desirable concentration and pH readjusted if required. Biotransformations were carried out in a final reaction volume of 1.0 mL in 2.0 mL Eppendorf tubes. Reactions were initiated by the addition of NAD^+ (1 mM), LE-AmDH-v1 or LE-AmDH-v27 (90 µM) and **1a** (10 mM), and were incubated at 30 °C in orbital shakers (170 rpm) for 24 h. Then, KOH (200 µL, 10 M) was added and extraction of the organic compounds was done using EtOAc (2 x 500µL, containing 10 mM IS).

Table S7. Screening of different ammonia containing buffers for synthesis of **1c** from **1a** using two LE-AmDH variants.

Analytical yield (%)							
Buffer	1	2	**3**	**4**	**5**	**6**	**7**
LE-AmDH-v1	2	0	0	0	1	0	0
LE-AmDH-v27	0	0	1	0	0	0	0

Direct conversion of **1a** (10 mM) to **1c** was observed with 2% analytical yield after 24 h using LE-AmDH-v1 (90 µM) in Tris-HCl (100 mM, pH 9, 0.5 M NH_4OH, NAD^+ 1 mM). Therefore, this buffer was used for further experiments.

For the cascade 1 (one-pot, one-step), Tris-HCl (100 mM) supplemented with NH_4OH (1 M) at pH 9 was used. Biocatalytic reactions (single replicates; 1.0 mL final reaction volume; 2.0 mL Eppendorf tubes) were performed in the presence of NAD^+ (1 mM), LE-AmDH (90 µM) and **1a** (10 mM). All of the six LE-AmDH variants were used in these reactions and a blank reaction (without LE-AmDH) was also included. Moreover, new incubation references were prepared by adding 10 mM of compounds (**1a**, **1b** and **1c**) to the reaction buffer (final volume of 1.0 mL). All of the samples were incubated at 30 °C in orbital shakers (170 rpm) for 48 h. Prior to extraction, 200 µL of 10 M KOH was added to each reaction. Extraction was carried out with EtOAc (containing 10 mM toluene and the samples were analyzed by GC-FID. The reported analytical yields are based on response factors (RF) of incubated references and reaction samples as explained previously.

For the cascade 2 (one-pot, two-step), the same buffer solutions and incubation references were used. Biocatalytic transformations (single replicates) were carried out in 1.0 mL reaction buffer (in 2.0 mL Eppendorf tubes). Reactions were started by adding NAD^+ (1 mM), NOx (10 µM), LE-AmDH (90 µM) and **1a** (20 mM) in consecutive order in 500 µL of Tris-HCl (100 mM) supplemented with NH_4OH (1 M) at pH 9. All six LE-AmDHs were tested. Moreover, a blank reaction was included (without LE-AmDHs). Incubation was

conducted at 30 °C in orbital shakers (170 rpm). After 24 h, another aliquot of 500 µL of the reaction buffer containing HCOONa (200 mM) and Cb-FDH (16 µM) was added. Therefore, the final concentrations in this second step were LE-AmDH variant (45 µM), Cb-FDH (8 µM) and HCOONa (100 mM). Then, the mixtures were incubated for another 24 h. After adding 200 µL of 10 M KOH, the samples were extracted with EtOAc (2 x 500 µL, containing 10 mM toluene). The organic phases were analyzed with GC-FID (split ratio 1:10). Calculations of conversion and yield are based on response factors of incubated references as described above.

6. Thermodynamic calculations

$\Delta_rG'^\circ$ represents the change of Gibbs free energy due to a chemical reaction in standard conditions (T 298.15 K, P 1 bar) in an aqueous buffer at a particular pH and ionic strength, and considering a standard 1 M concentration of reagents and products.

$\Delta_rG'^m$ represents the change of Gibbs free energy due to a chemical reaction in standard conditions (T 298.15 K, P 1 bar) in an aqueous buffer at a particular pH and ionic strength, and considering the physiological 1 mM concentration of reagents and products.

Δ_rG' represents the change of Gibbs free energy due to a chemical reaction in standard conditions (T 298.15 K, P 1 bar) in an aqueous buffer at a particular pH and ionic strength, and considering concentration values of reagents and products that correlated with our experimental determinations. Therefore, these values must be taken in greater consideration for the analysis.

For the sake of completeness, all of the three values are reported for each investigated reaction.

The calculations were performed with the aid of eQuilibrator, a tool made available by Milo Lab at the Weizmann Institute in Rehovot, Israel.[5] eQuilibrator uses a well-studied approximation of Δ_fG (the ΔG related to the formation of a given molecule from its components) called group contribution, thus enabling thermodynamic analysis of many biochemical reactions and pathways.[6] eQuilibrator also allows for manipulation of the conditions of a reaction—pH, ionic strength, and reactant and product concentrations—to help exploring the thermodynamic landscape of a biochemical reaction.

Reaction 1 (carbonyl reduction of benzaldehyde 1b)

Benzaldehyde (aq.) + NADH (aq.) + H^+ → Benzylic alcohol (aq.) + NAD^+ (aq.)

**General conditions (aqueous buffer)**	
pH	7
Ionic strength	100 mM

**Considered specific conditions (for Δ_rG' calculation)**	
[Benzaldehyde]	20 mM
[NADH]	1 mM
[Benzylic alcohol]	0.1 mM
[NAD^+]	0.01 mM

**Results**	
$\Delta_rG'^\circ$	-20.8 + 2.4 KJ mol^{-1}
$\Delta_rG'^m$	-20.8 + 2.4 KJ mol^{-1}
**Δ_rG'**	**-45.4 ± 2.4 KJ mol^{-1}**

Reaction 2 (reductive amination of benzaldehyde 1b)

Benzaldehyde (aq.) + NADH (aq.) + H^+ + Ammonia (aq.)→ Benzylic alcohol (aq.) + NAD^+ (aq.) + Water (l)

**General conditions (aqueous buffer)**	
pH	9
Ionic strength	2 M

**Considered specific conditions (for Δ_rG' calculation)**	
[Benzaldehyde]	20 mM
[NADH]	1 mM
[Ammonia]	2 M
[Benzylamine]	0.1 mM
[NAD^+]	0.01 mM
[Water]	55 M

**Results**	
$\Delta_rG'^{\circ}$	-22.4 + 5.9 KJ mol^{-1}
$\Delta_rG'^{m}$	-5.3 + 5.9 KJ mol^{-1}
**Δ_rG'**	**-48.6 ± 5.9 KJ mol^{-1}**

Reaction 3 (carbonyl reduction of acetophenone 11b)

Acetophenone (aq.) + NADH (aq.) + H^+ → 1-Phenylethanol (aq.) + NAD^+ (aq.)

**General conditions (aqueous buffer)**	
pH	7
Ionic strength	100 mM

**Considered specific conditions (for Δ_rG' calculation)**	
[Acetophenone]	20 mM
[NADH]	1 mM
[1-Phenylethanol]	0.1 mM
[NAD^+]	0.01 mM

**Results**	
$\Delta_rG'^{\circ}$	-20.6 + 1.9 KJ mol^{-1}
$\Delta_rG'^{m}$	-20.6 + 1.9 KJ mol^{-1}
**Δ_rG'**	**-45.2 ± 1.9 KJ mol^{-1}**

Reaction 4 (reductive amination of acetophenone 11b)

Acetophenone (aq.) + NADH (aq.) + H^+ + Ammonia (aq.)→ 1-Phenylethylamine (aq.) + NAD^+ (aq.) + Water (l)

**General conditions (aqueous buffer)**	
pH	9
Ionic strength	2 M

**Considered specific conditions (for $\Delta_r G'$ calculation)**	
[Acetophenone]	20 mM
[NADH]	1 mM
[Ammonia]	2 M
[1-Phenylethylamine]	0.1 mM
[NAD^+]	0.01 mM
[Water]	55 M

**Results**	
$\Delta_r G'^\circ$	-16.0 + 3.6 KJ mol^{-1}
$\Delta_r G'^m$	1.1 + 3.6 KJ mol^{-1}
**$\Delta_r G'$**	**-42.2 ± 3.6 KJ mol^{-1}**

7. Analytical methods

Conversions and yields were determined by gas chromatography using an Agilent 7890B chromatograph, equipped with a FID detector. An Agilent J&W DB1701 (30 m, 250 µm, 0.25 µm) column was used and H_2 was applied as carrier gas. Unless stated otherwise, the injection volume was 1 µL with following method:

- Constant pressure = 6.9 psi; Flow = 1.05 mL min^{-1}; T injector = 250 °C.
- Temperature program: T initial = 60 °C; hold 6.5 min; gradient 20 °C min^{-1} up to 100 °C; hold 1 min; gradient 20 °C min^{-1} up to 280 °C; hold 1 min.

Enantiomeric excess of the derivatized amines was measured using a CP-Chirasil Dex-CB column (25 m, 320 µm, 0.25 µm) with following method:

- Constant pressure = 3.8 psi; Flow = 1.4 mL min^{-1}; T injector = 200 °C. Split ratio 1:20.
- Temperature program: T initial = 100 °C; hold 2 min; gradient 1 °C min^{-1} up to 130 °C; hold 5 min; gradient 10 °C min^{-1} up to 170 °C; hold 10 min; gradient 10 °C min^{-1} up to 180 °C; hold 1 min.

Table S8. Retention times of the compounds used in this study

Compound	Retention time (min)	Compound	Retention time (min)	Compound	Retention time (min)
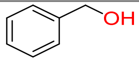	12.6	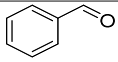	10.7	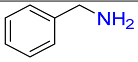	11.3
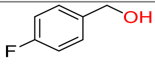	12.9	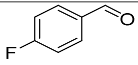	10.7	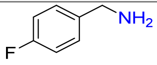	11.7
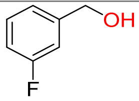	13.1	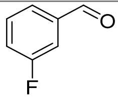	10.4	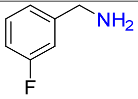	11.8
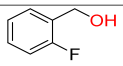	12.7	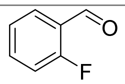	10.2	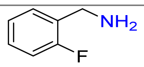	11.4
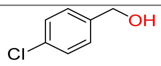	15.0	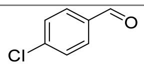	13.3	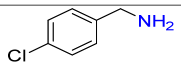	14.2
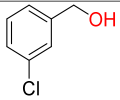	14.4	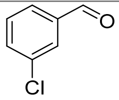	12.5		
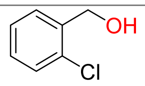	14.0	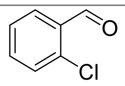	12.3		
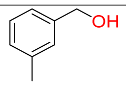	13.7	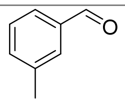	12.4	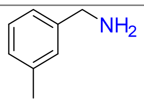	12.8
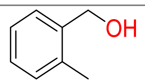	13.8	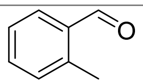	12.4	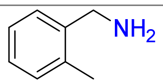	13.0
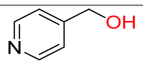	14.0	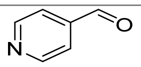	10.5	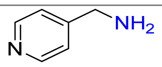	13.5

Compound	Retention time (min)	Compound	Retention time (min)	Compound	Retention time (min)
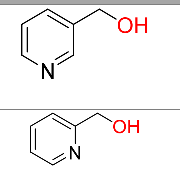	13.8	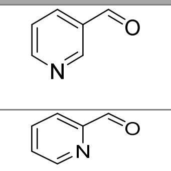	11.0	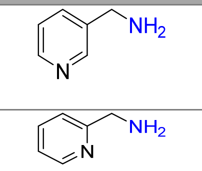	13.3
	12.0		9.6		12.0
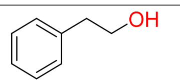	13.4	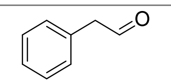	12.3	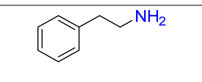	12.6
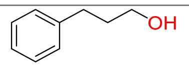	14.6	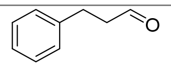	13.8	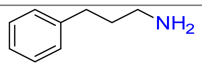	13.9
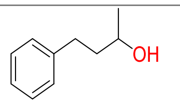	14.7	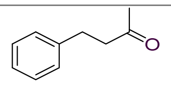	15.5	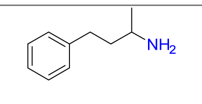	15.1
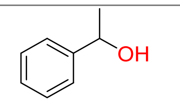	12.6	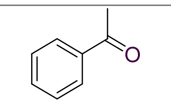	13.4	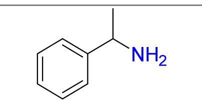	12.6
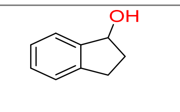	14.6	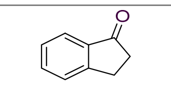	16.1	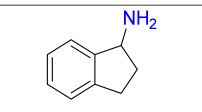	15.0
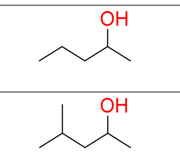	4.9	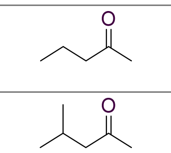	2.9	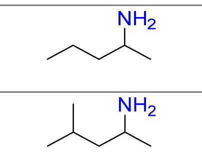	2.7
	6.4		3.9		3.5
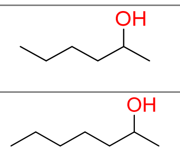	7.6	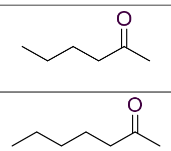	5.1	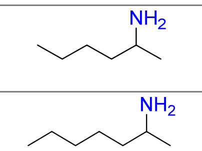	4.0
	9.9		8.1		6.8
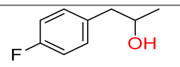	-	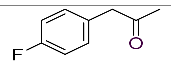	13.5	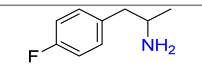	12.9
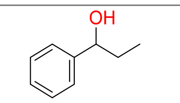	-	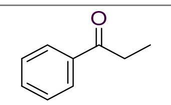	13.4	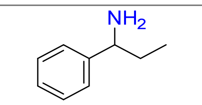	12.7
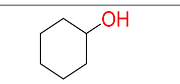	-	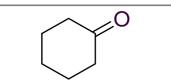	8.8	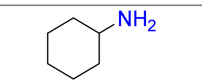	6.4
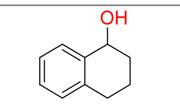	16.4	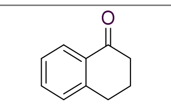	15.7	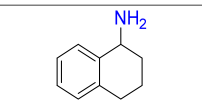	15.1
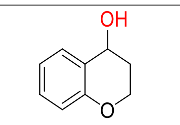	16.6	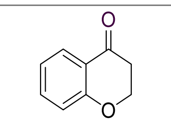	15.4	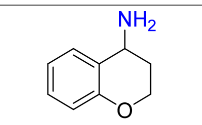	15.3

8. Steady-state kinetics for LE-AmDH-v1, LE-AmDH-v22 and LE-AmDH-v25

Determination of the saturation concentrations of NADH for LE-AmDH-v22 and v25

Note: saturation concentration of NADH for LE-AmDH-v1 (250 µM) was determined in our previous study.[1] Saturation concentrations of NADH for LE-AmDH-v22 and v25 were determined in this work (**Table S9**).

Table S9. Determination of the saturation concentration of NADH for LE-AmDH-v22 and LE-AmDH-v25 at 60 °C. [LE-AmDH-v22]: 23.4 µM, [LE-AmDH-v25]: 26.1 µM [**14b**]: 25 mM, [NADH]:0-150 µM. Method: reduction of the absorbance of NADH at 340 nm

Enzyme	$K_{M\ app}$(µM)	k_{app} (min^{-1})	conc. NADH employed (µM)
LE-AmDH-v22	4.79 ± 0.87	0.28 ± 0.00	150
LE-AmDH-v25	14.25 ± 4.05	0.34 ± 0.02	150

Steady-state kinetics

Two sets of independent measurements were performed. Varied substrate concentrations (from a 500 mM main stock in DMSO in the case of substrate **2b**, or from a 500 mM stock in DMSO which was further diluted to 350 mM in the reaction buffer in the case of substrates **14b** and **15b**) were added to a pre-incubated buffer (70 °C), and further incubated at the desired measuring temperature (60 °C) in the thermostatic controlled cuvette holder of the UV-vis spectrophotometer for 2 min. The enzyme was added after which the reaction was initiated by the addition of NADH.

The initial velocities were calculated from the linear range of the fitted trend line of the progress curve and were then plotted against the substrate concentration to obtain the kinetic parameter (**Table S10**, **Fig. S4-S9**)

Table S10. Kinetic assay for the determination of the steady-state kinetics for LE-AmDH-v1, -v2 and –v25.

Enzyme	Reaction	substrate (mM)	NADH (µM)	enzyme (µM)	$K_{M\ app}$ (mM)	k_{app} (min^{-1})	$k_{app}/K_{M\ app}$ ($M^{-1}\ min^{-1}$)
LE-AmDH-v1	**2b** to **2c**	0-16[a]	250	1.3	3.62 ± 0.46	22.02 ± 0.90	6082
	**2b** to **2a**	0-16[a]	250	26.9	0.55 ± 0.10	0.15 ± 0.00	272
LE-AmDH-v22	**14b** to **14c**	0-30[b]	150	23.4	9.00 ± 0.80	0.29 ± 0.01	32
	**15b** to **15c**	0-30[b]	150	23.4	4.70 ± 0.55	0.67 ± 0.02	143
LE-AmDH-v25	**14b** to **14c**	0-30[b]	150	26.1	3.38 ± 0.33	0.24 ± 0.01	71
	**15b** to **15c**	0-30[b]	150	26.1	6.50 ± 0.55	0.24 ± 0.01	37

[a] Directly added as a 500 mM stock solution in DMSO; solubility problems were observed at substrate concentration above 16 mM.
[b] Added as a 500 mM main stock solution in DMSO, which was further diluted to 350 mM stock with the reaction buffer.

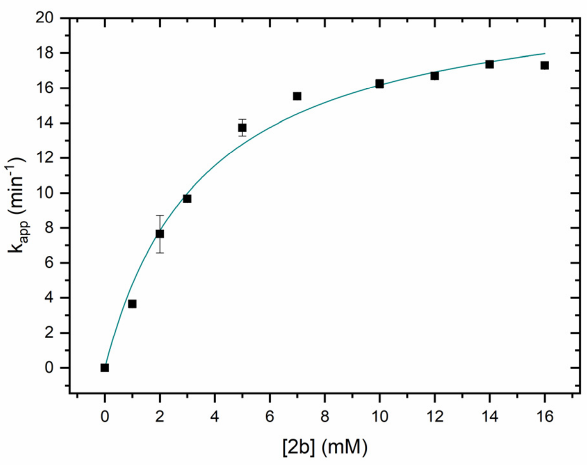

Fig. S4. Hyperbolic fit of the reaction rates obtained for the reductive amination of **2b** to **2c** catalyzed by LE-AmDH-v1 at 60 °C. Kinetic study could be performed until 16 mM substrate concentration due to solubility issues above this value.

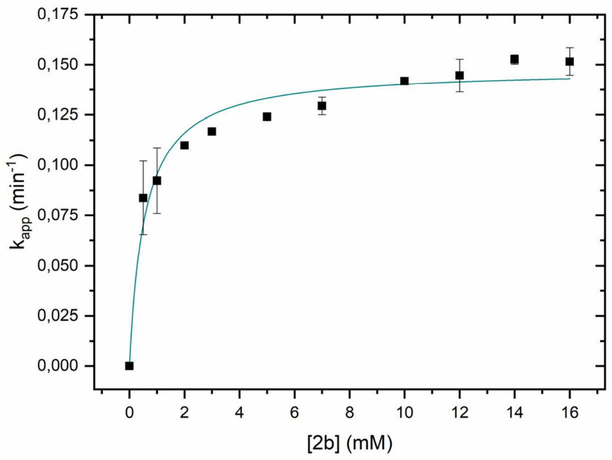

Fig. S5. Hyperbolic fit of the reaction rates obtained for the reduction of **2b** to **2a** catalyzed by LE-AmDH-v1 at 60 °C. Kinetic study could be performed until 16 mM substrate concentration due to solubility issues above this value.

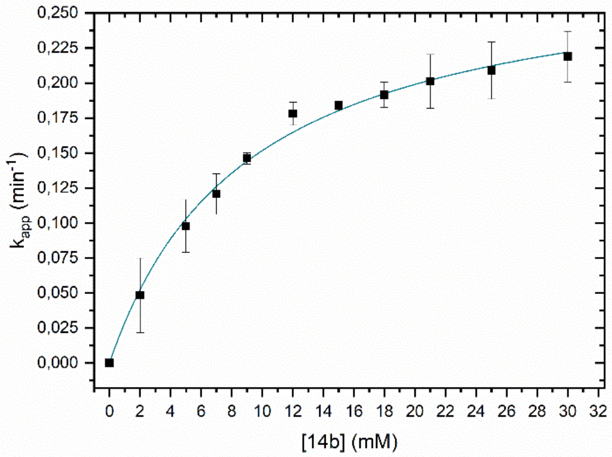

Fig. S6. Hyperbolic fit of the reaction rates obtained for the reductive amination of **14b** to **14c** catalyzed by LE-AmDH-v22

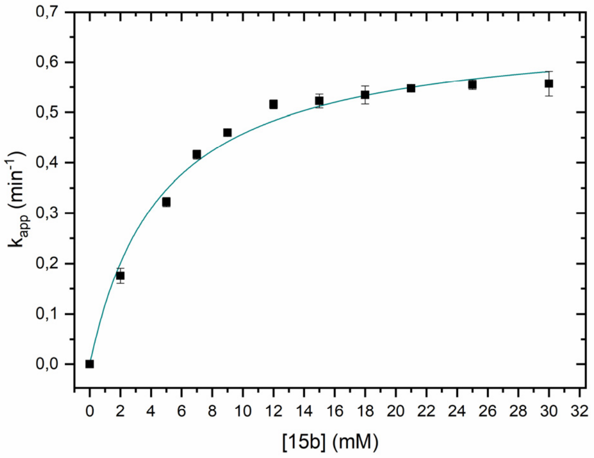

Fig. S7. Hyperbolic fit of the reaction rates obtained for the reductive amination of **15b** to **15c** catalyzed by LE-AmDH-v22

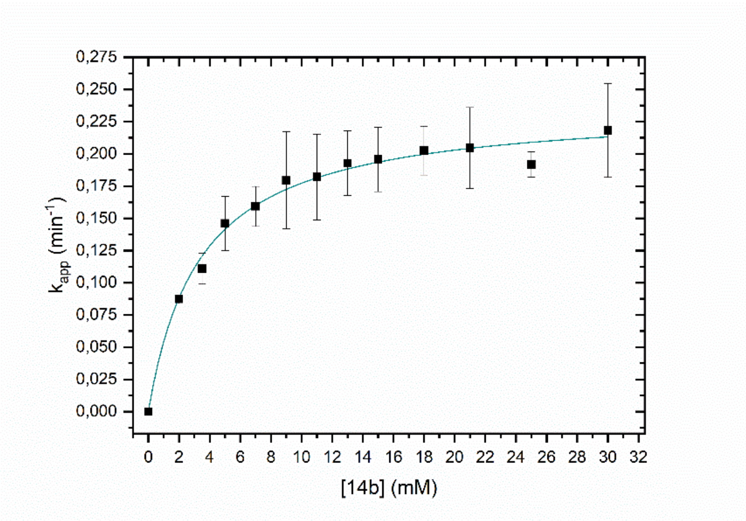

Fig. S8. Hyperbolic fit of the reaction rates obtained for the reductive amination of **14b** to **15c** catalyzed by LE-AmDH-v25

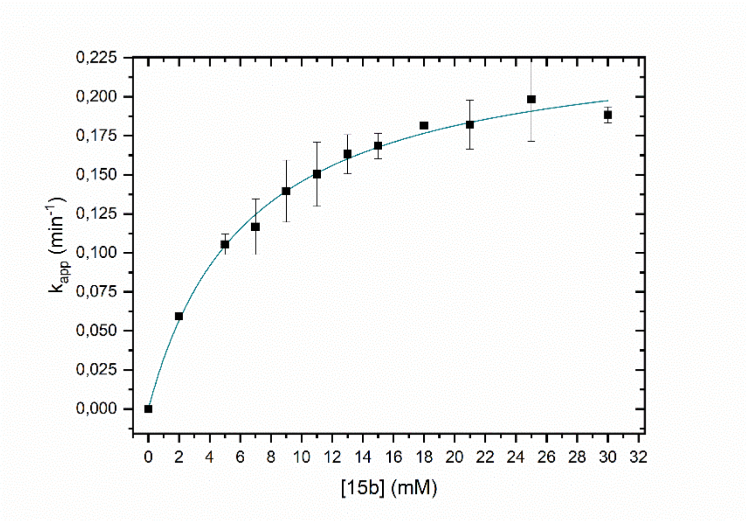

Fig. S9. Hyperbolic fit of the reaction rates obtained for the reductive amination of **15b** to **15**c catalyzed by LE-AmDH-v25

9. Determination of enzymatic specific activities

Two sets of independent measurements were performed. Fixed substrate concentrations (from a 500 mM main stock solution in DMSO in the case of substrate **2b**, or from a 500 mM stock which was further diluted to 350 mM with the reaction buffer in the case of substrates **11b**) were added to a pre-incubated buffer (70 °C) and further incubated at the desired measuring temperature (60 °C) in the thermostatic controlled cuvette holder of the UV-vis spectrophotometer for 2 min. The enzyme was added after which the reaction was initiated by the addition of NADH at fixed final concentration of 250 µM.

The initial velocities were calculated from the linear range of the fitted trend line of the progress curve and were then divided by the enzyme concentration to obtain the specific activities in U per µmol of enzyme (**Table S11** and **Table S12**).

Table S11. Apparent specific activities of the LE-AmDH variants for the reductive amination of **11b**.

Enzyme	Reaction	11b (mM)	Enzyme (µM)	Activity (U µmol^{-1})
LE-AmDH-v1	**11b** to **11c**	20	6	4.78 ± 0,39
LE-AmDH-v22		20	6	9.22 ± 0.56
LE-AmDH-v24		20	6	1.24 ± 0.03
LE-AmDH-v25		20	6	7.04 ± 0.07
LE-AmDH-v27		20	6	0.52 ± 0.04
Wild type		20	6	n.d.[a]

[a] n.d.: not detected due to too low activity

Table S12. Apparent specific activities of the LE-AmDH variants for the reductive amination and reduction of **2b**.

Enzyme	Reaction	2b (mM)	Enzyme (µM)	Activity (U µmol^{-1})	Reaction	2b (mM)	Enzyme (µM)	Activity (U µmol^{-1})
LE-AmDH-v1	**2b** to **2c**	15	6	8.90 ± 0.03	**2b** to **2a**	15	30	0.12 ± 0.00
LE-AmDH-v22		15	6	30.16 ± 1.32		15	30	0.19 ± 0.01
LE-AmDH-v24		15	6	2.34 ± 0.06		15	30	0.12 ± 0.01
LE-AmDH-v25		15	6	20.02 ± 1.55		15	30	0.16 ± 0.01
LE-AmDH-v27		15	6	0.95 ± 0.01		15	30	0.39 ± 0.01
Wild type		15	6	0.24 ± 0.03		15	30	0.36 ± 0.00

10. Chromatograms

10.1 GC-FID chromatograms of the biocatalytic reductive aminations

Reference compounds **1a**, **1b** and **1c**

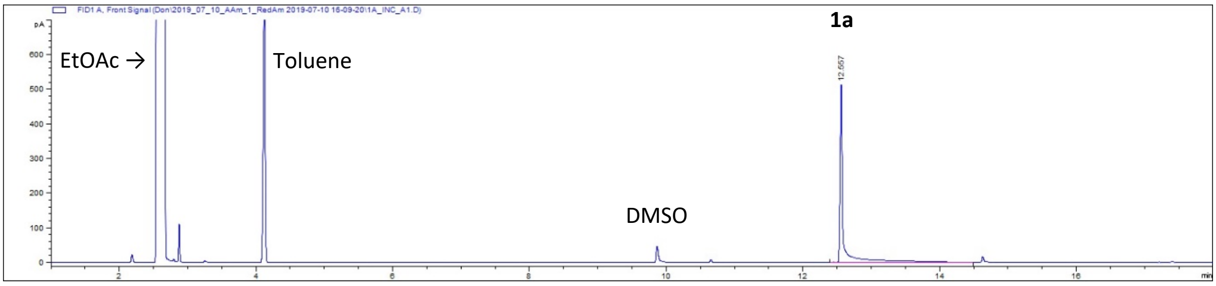

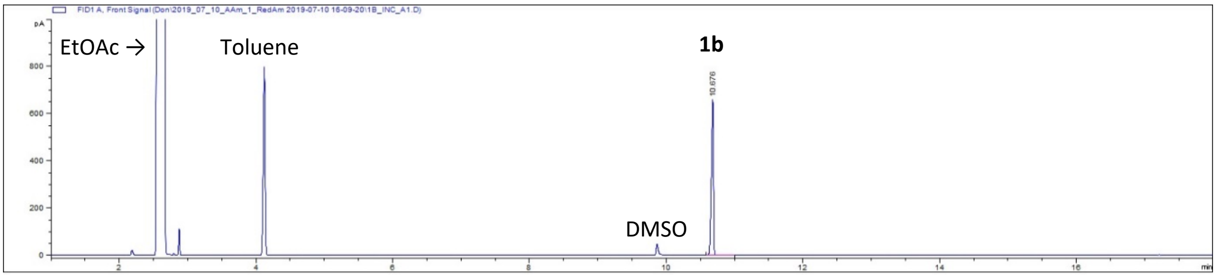

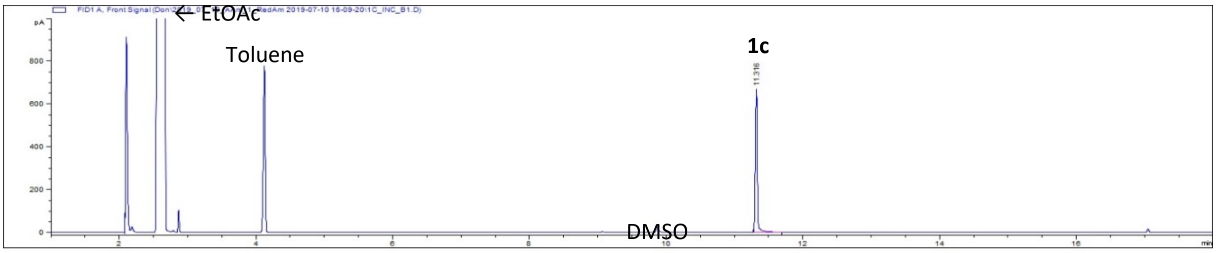

Biocatalytic reductive amination of **1b** catalyzed by LE-AmDH-v22 (acidic extract)

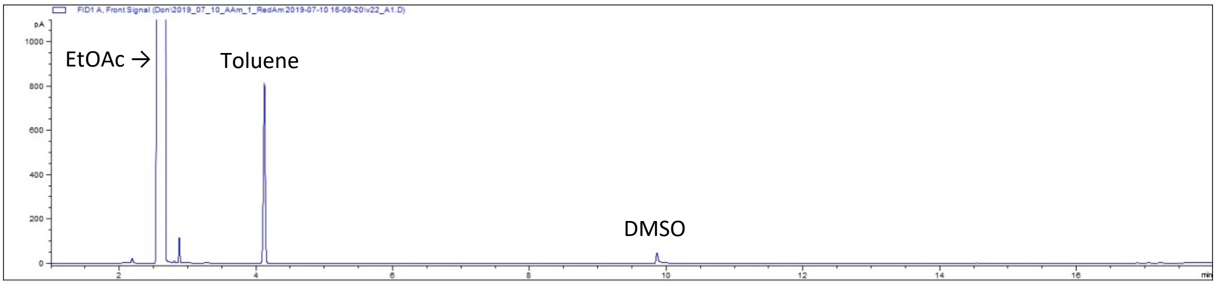

Biocatalytic reductive amination of **1b** catalyzed by LE-AmDH-v22 (basic extract)

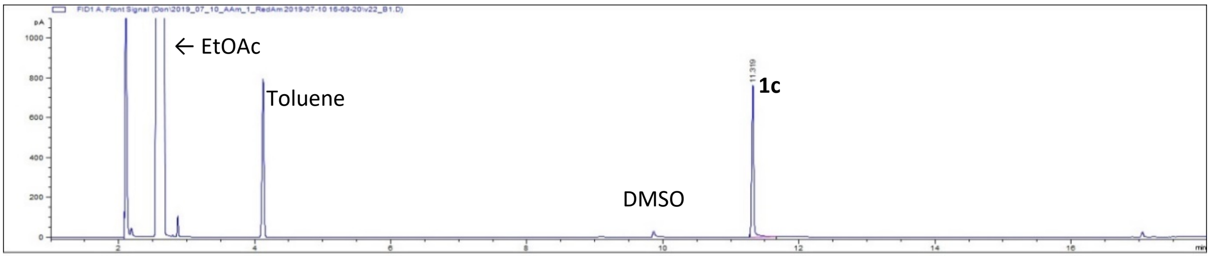

Reference compounds **2a**, **2b** and **2c**

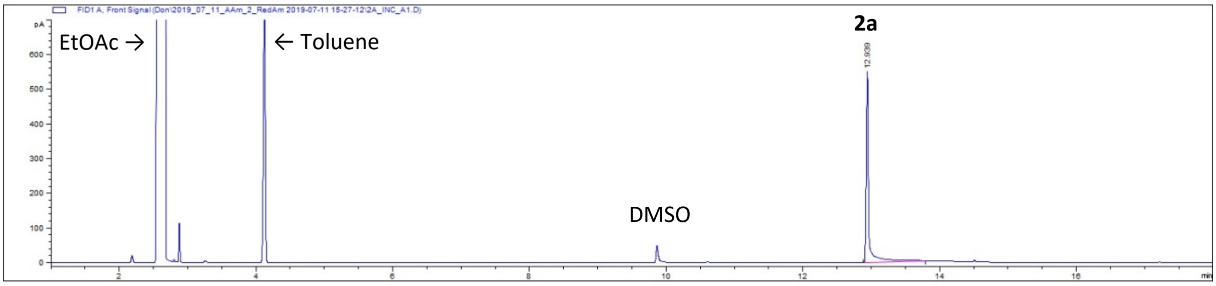

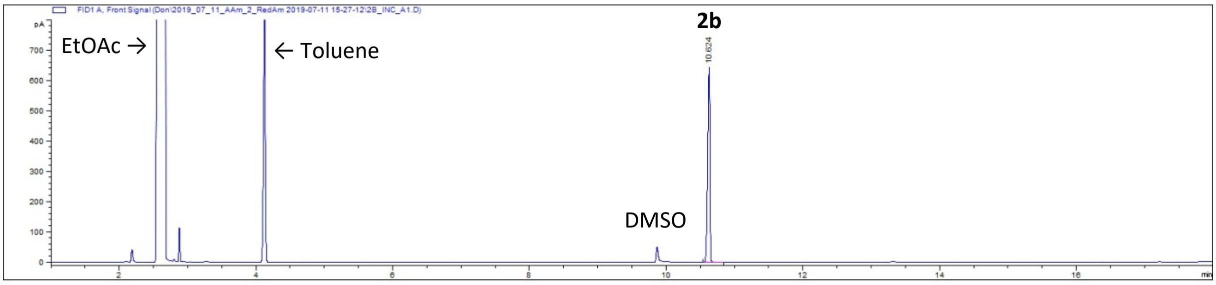

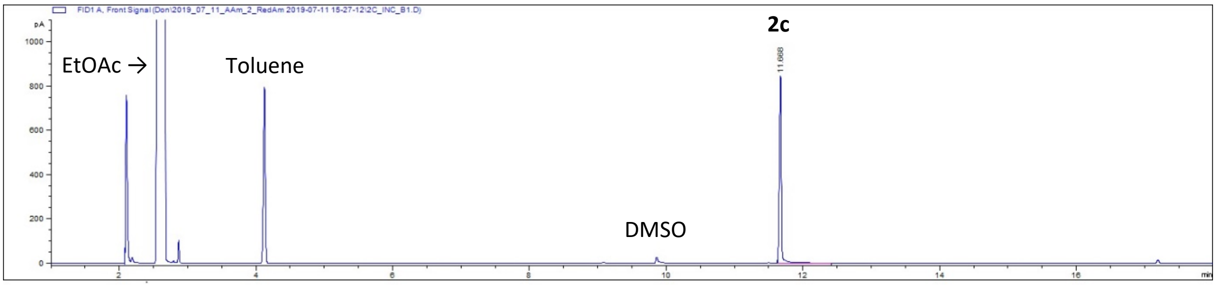

Biocatalytic reductive amination of **2b** catalyzed by LE-AmDH-v22 (acidic extract)

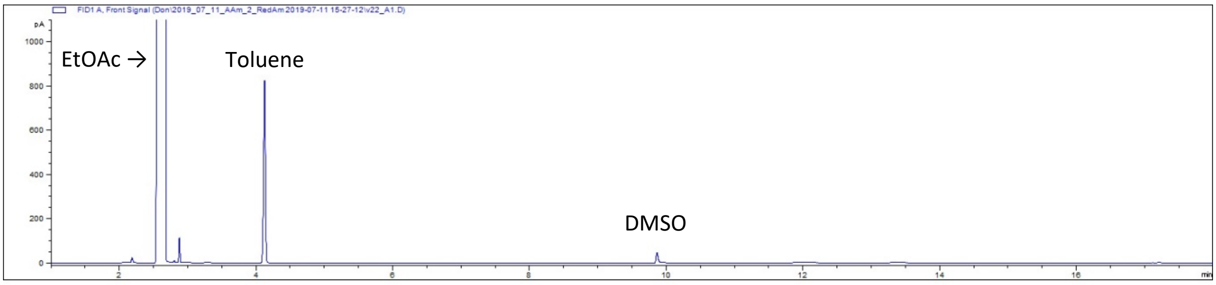

Biocatalytic reductive amination of **2b** catalyzed by LE-AmDH-v22 (basic extract)

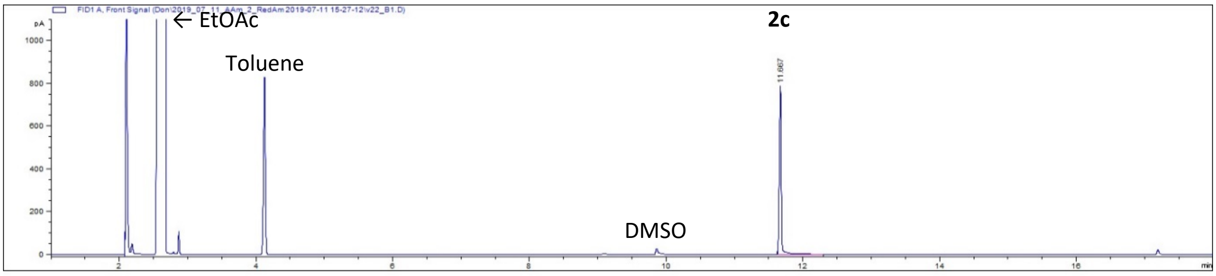

Reference compounds **3a**, **3b** and **3c**

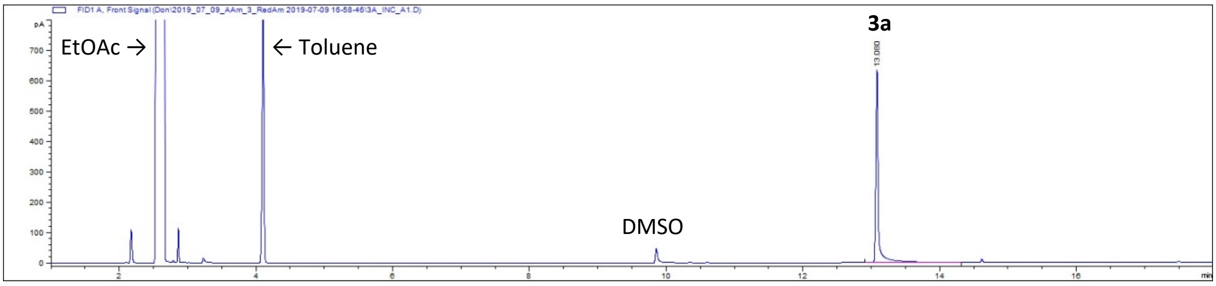

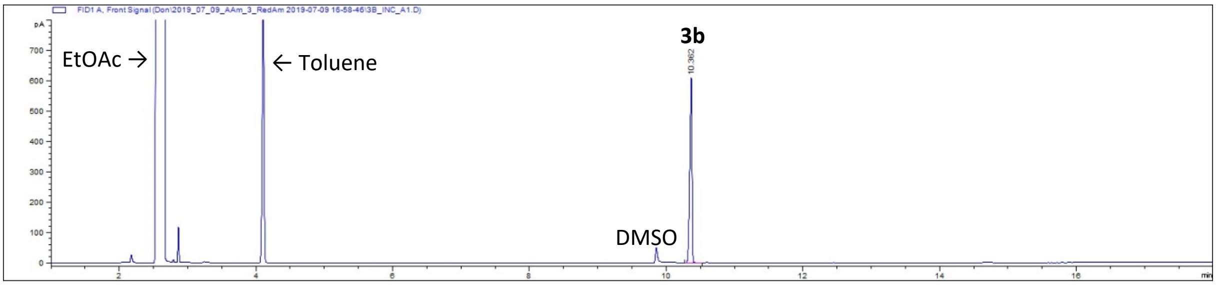

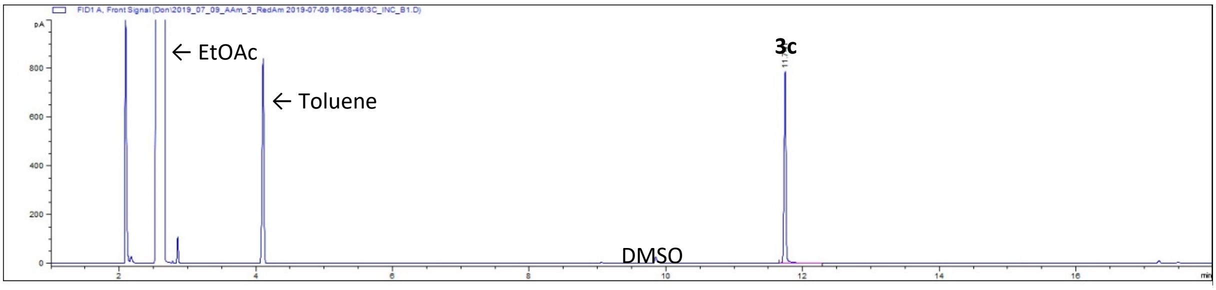

Biocatalytic reductive amination of **3b** catalyzed by LE-AmDH-v22 (acidic extract)

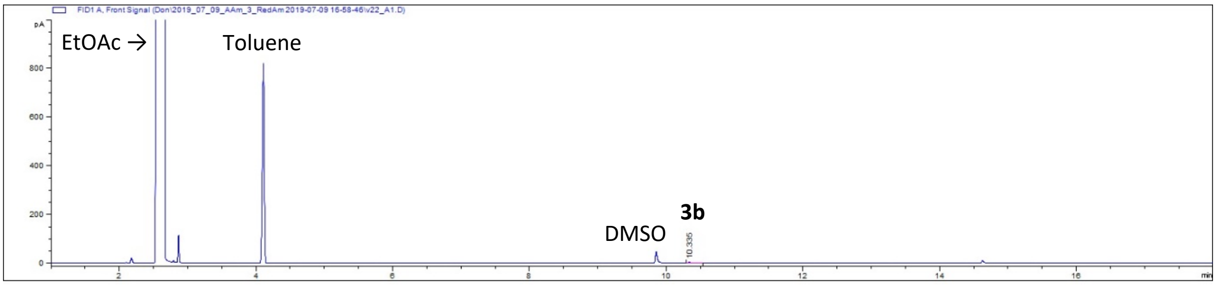

Biocatalytic reductive amination of **3b** catalyzed by LE-AmDH-v22 (basic extract)

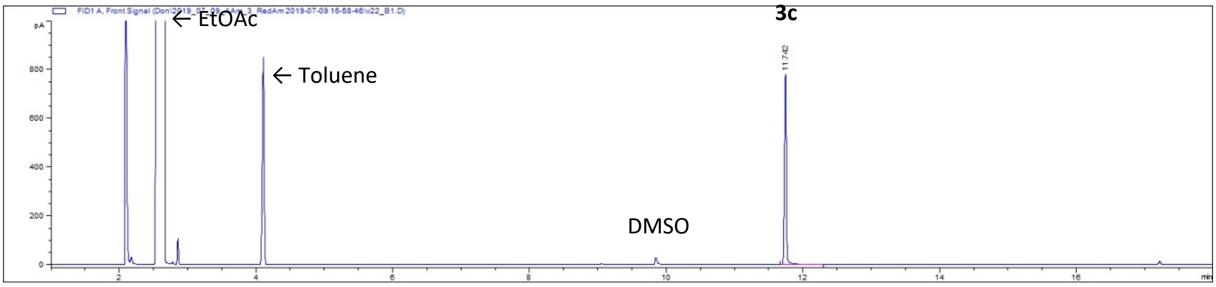

Reference compounds **4a**, **4b** and **4c**

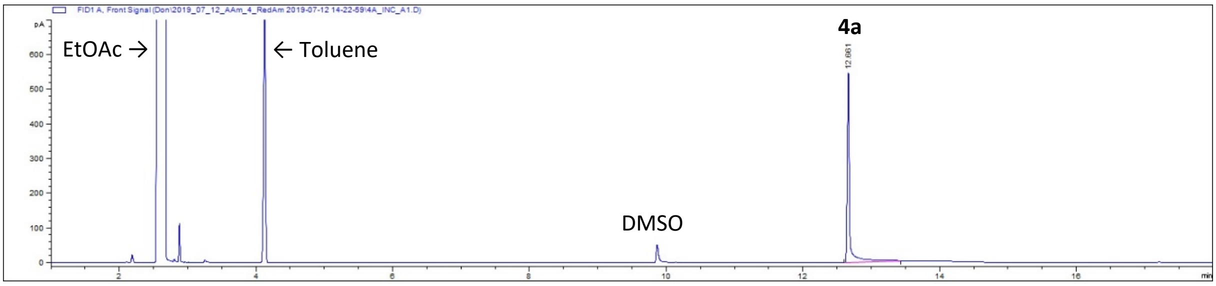

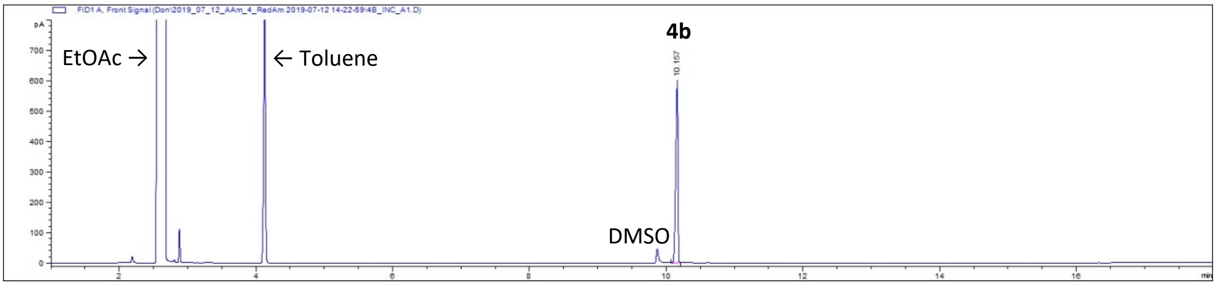

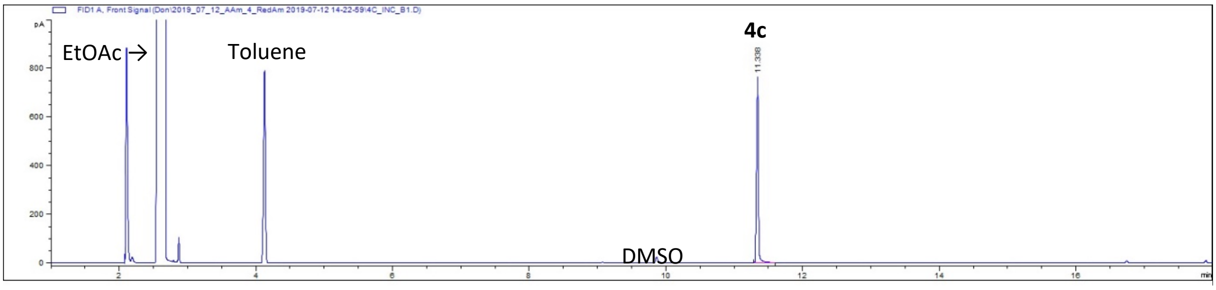

Biocatalytic reductive amination of **4b** catalyzed by LE-AmDH-v22 (acidic extract)

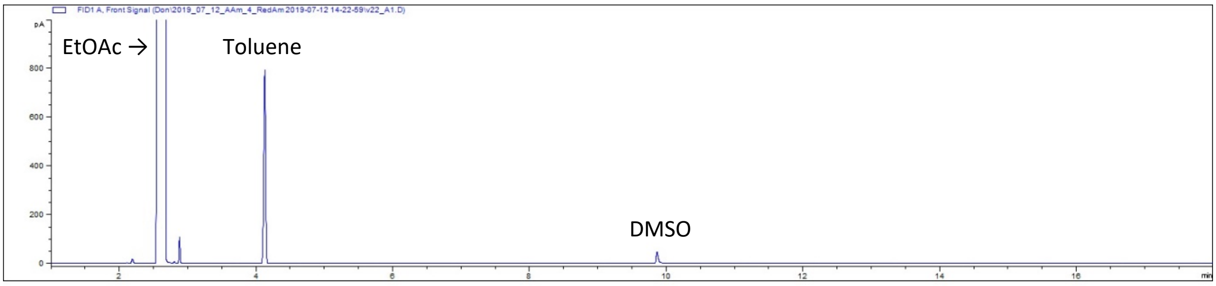

Biocatalytic reductive amination of **4b** catalyzed by LE-AmDH-v22 (basic extract)

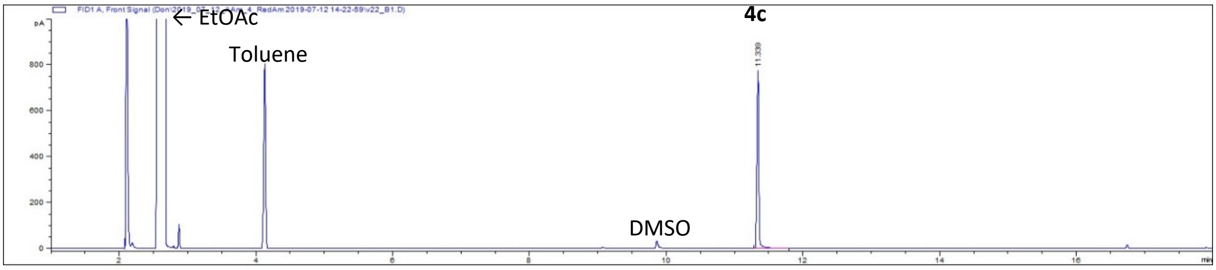

Reference compounds **5a**, **5b** and **5c**

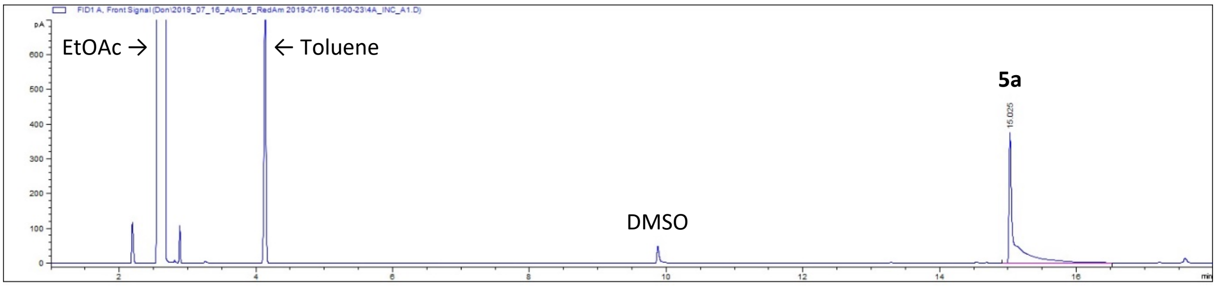

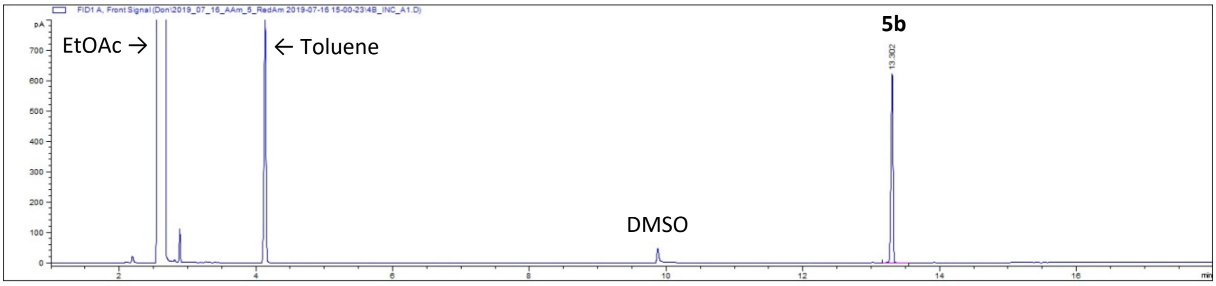

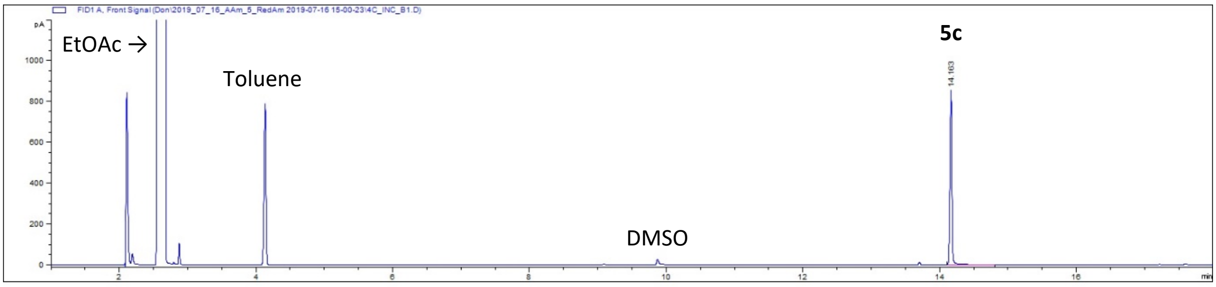

Biocatalytic reductive amination of **5b** catalyzed by LE-AmDH-v22 (acidic extract)

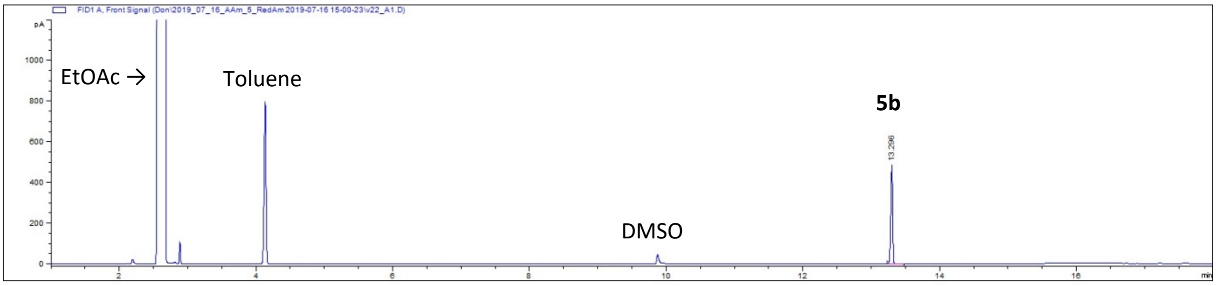

Biocatalytic reductive amination of **5b** catalyzed by LE-AmDH-v22 (basic extract)

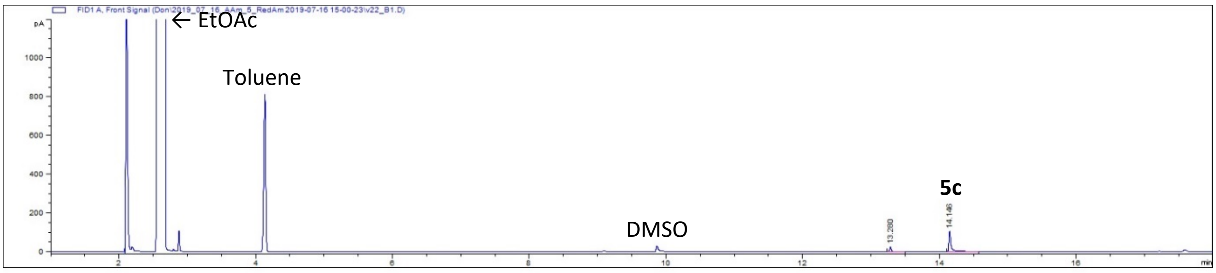

Reference compounds **6a**, **6b** and **6c**

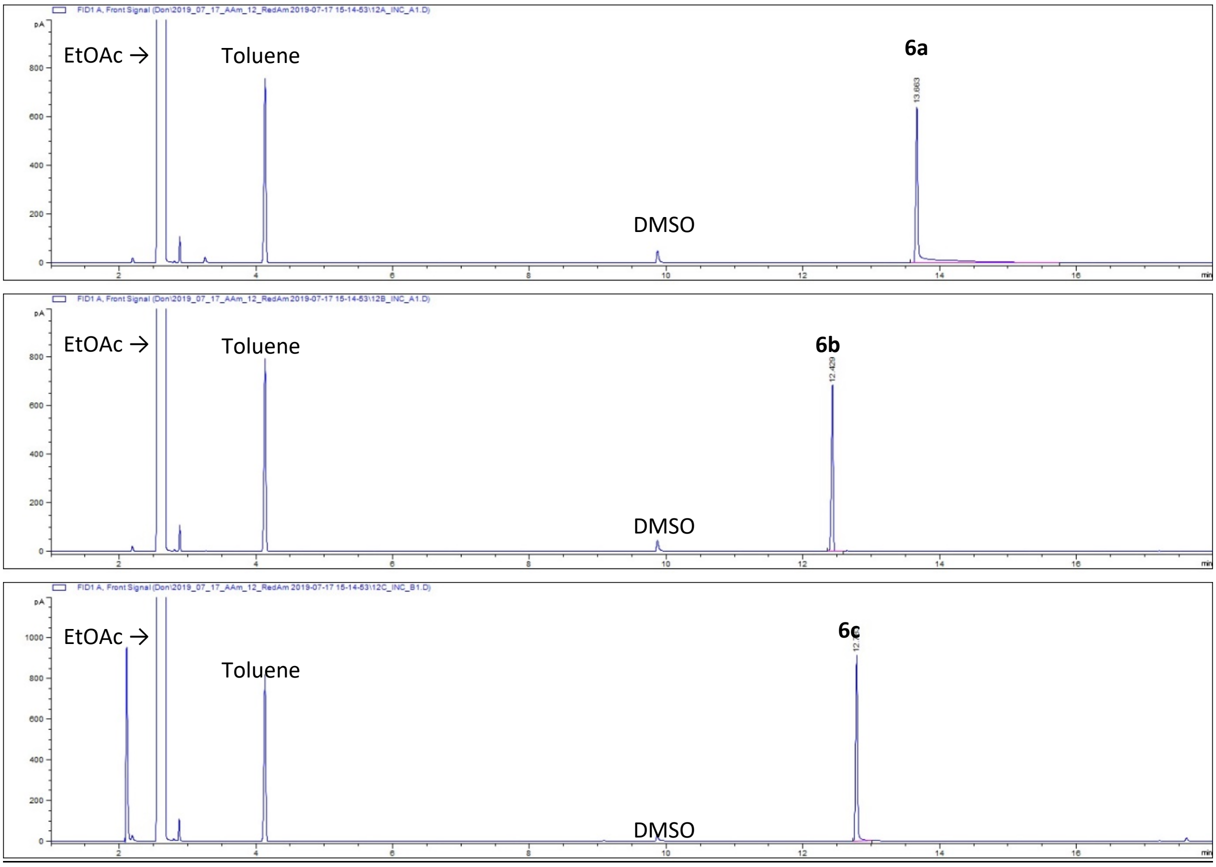

Biocatalytic reductive amination of **6b** catalyzed by LE-AmDH-v22 (acidic extract)

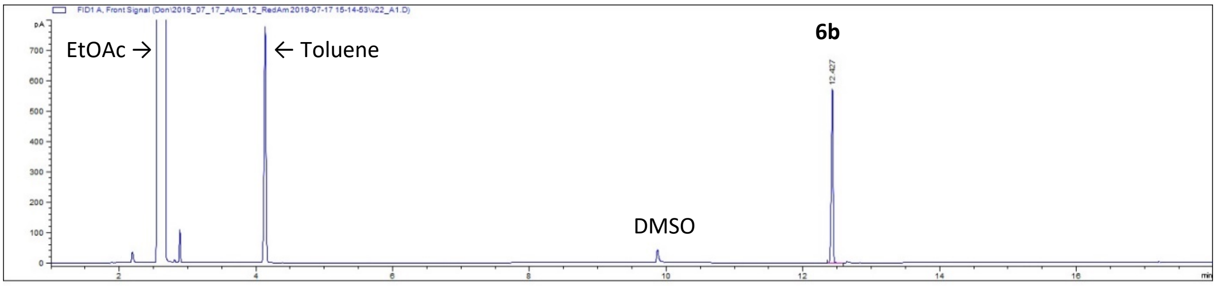

Biocatalytic reductive amination of **6b** catalyzed by LE-AmDH-v22 (basic extract)

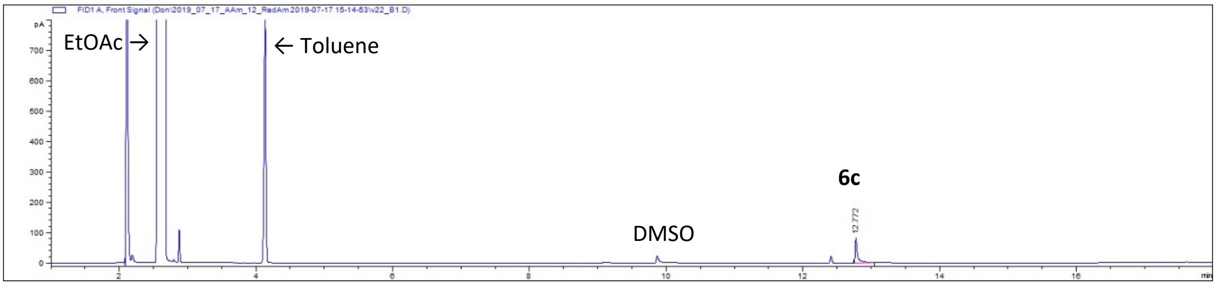

Reference compounds **7a**, **7b** and **7c**

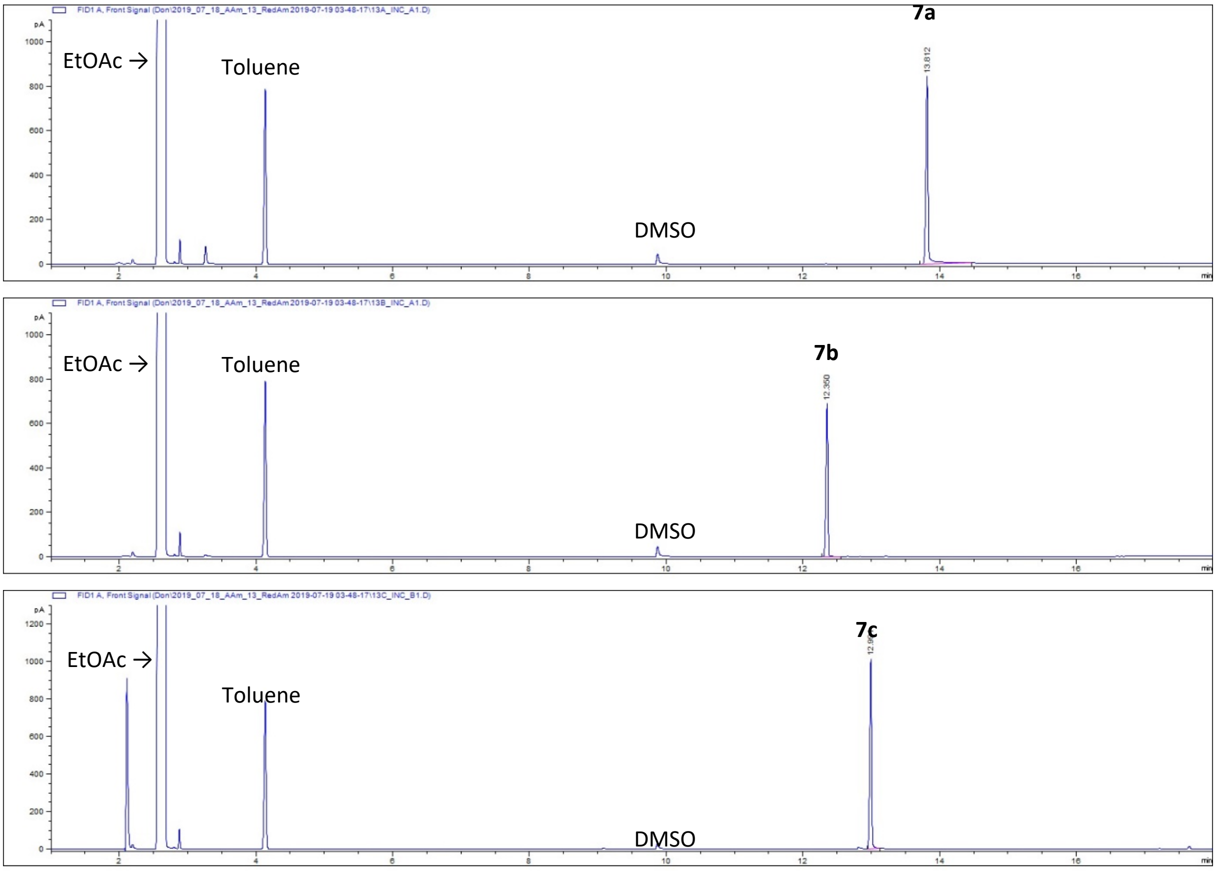

Biocatalytic reductive amination of **7b** catalyzed by LE-AmDH-v22 (acidic extract)

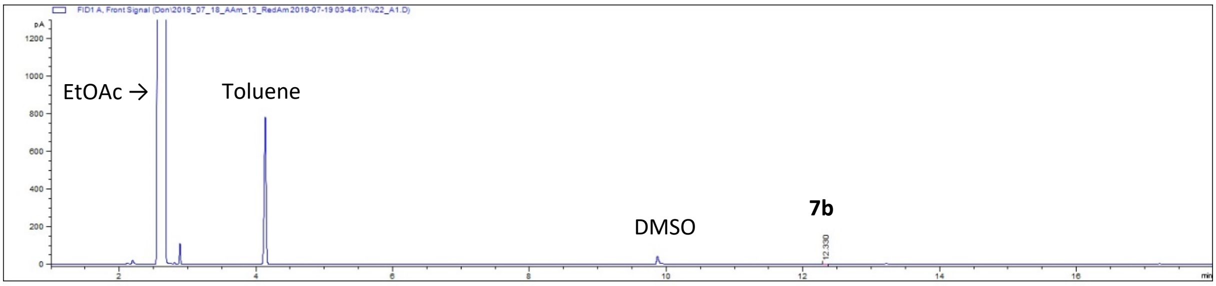

Biocatalytic reductive amination of **7b** catalyzed by LE-AmDH-v22 (basic extract)

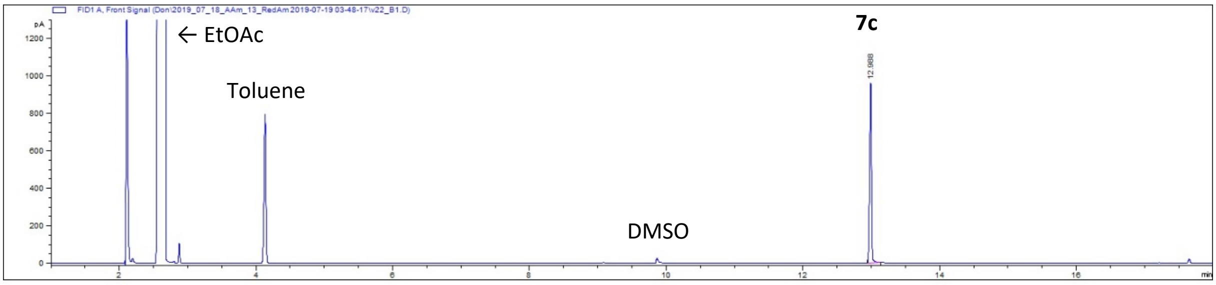

Reference compounds **8a**, **8b** and **8c**

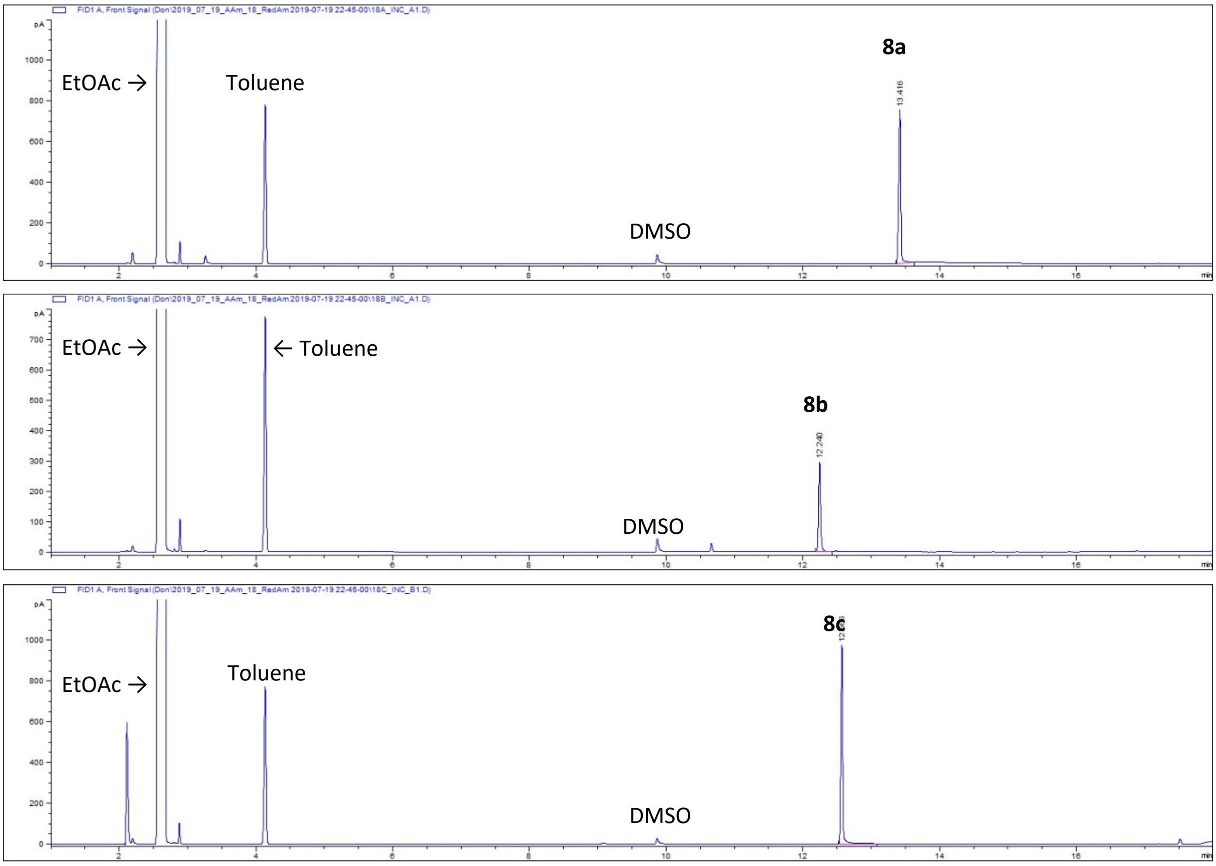

Biocatalytic reductive amination of **8b** catalyzed by LE-AmDH-v22 (acidic extract)

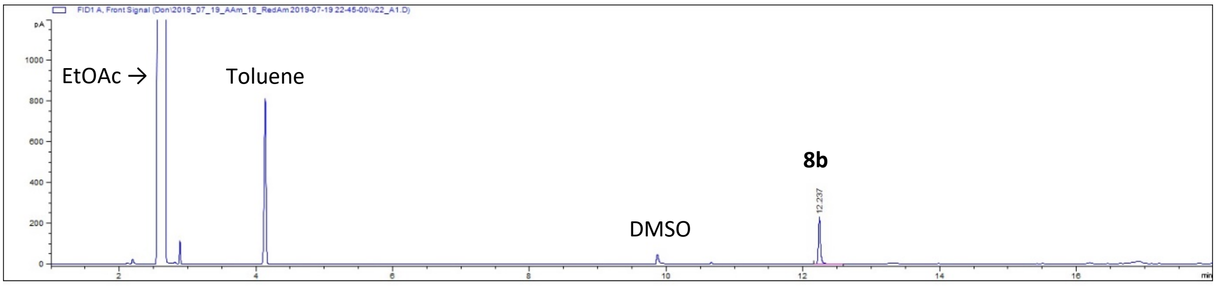

Biocatalytic reductive amination of **8b** catalyzed by LE-AmDH-v22 (basic extract)

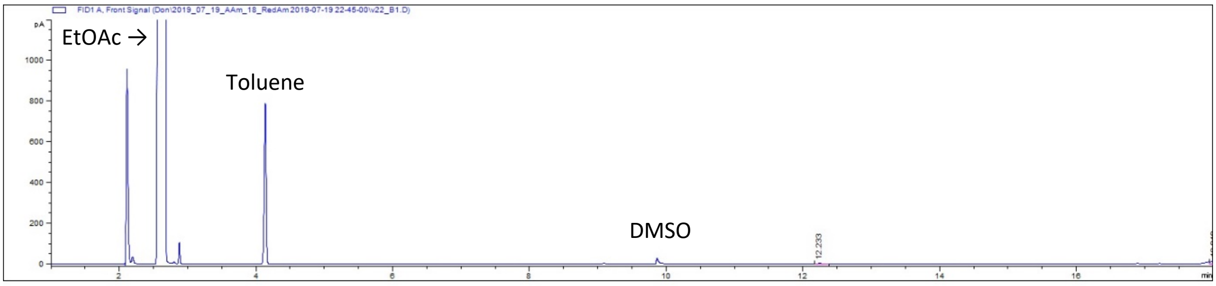

Reference compounds **9a**, **9b** and **9c**

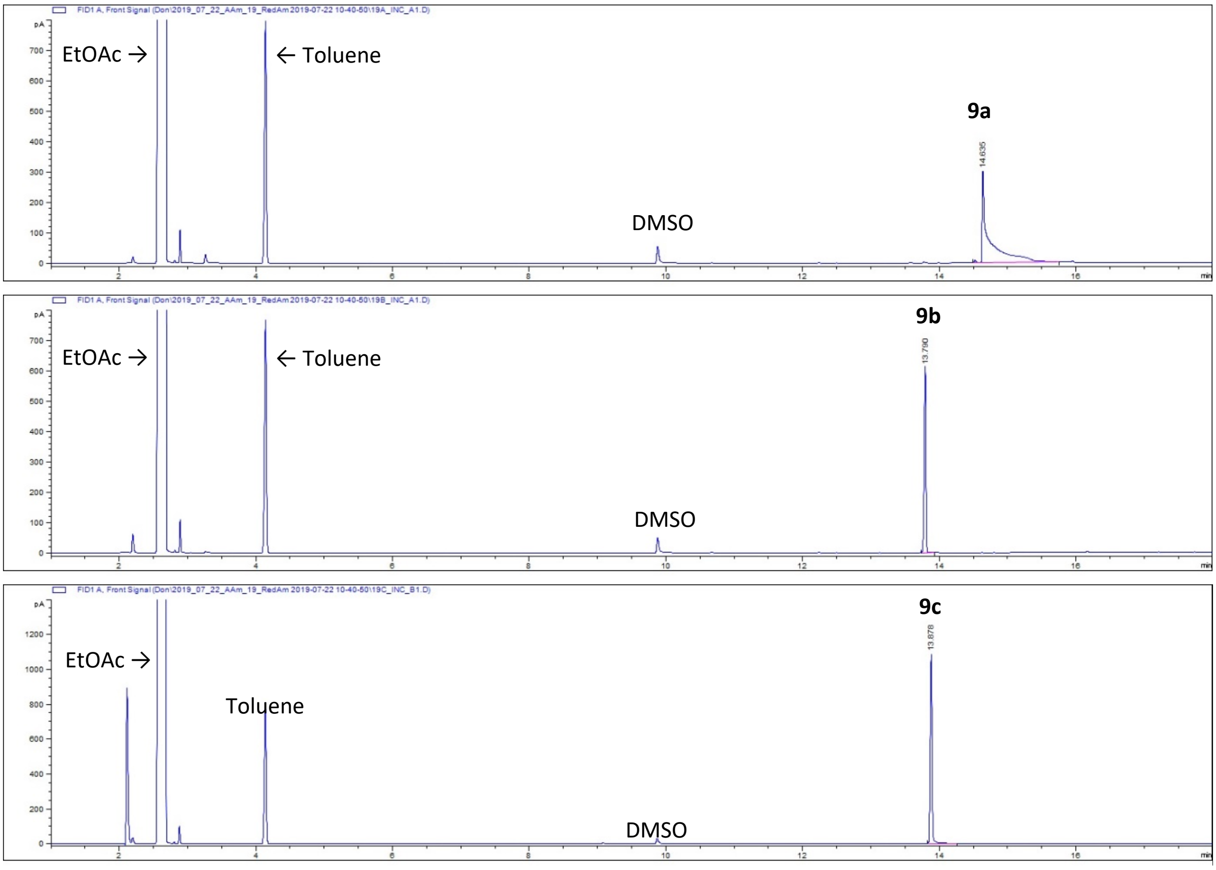

Biocatalytic reductive amination of **9b** catalyzed by LE-AmDH-v22 (acidic extract)

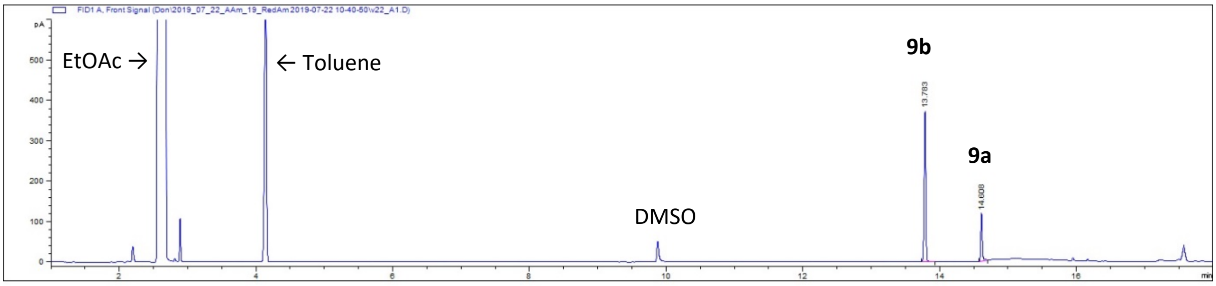

Biocatalytic reductive amination of **9b** catalyzed by LE-AmDH-v22 (basic extract)

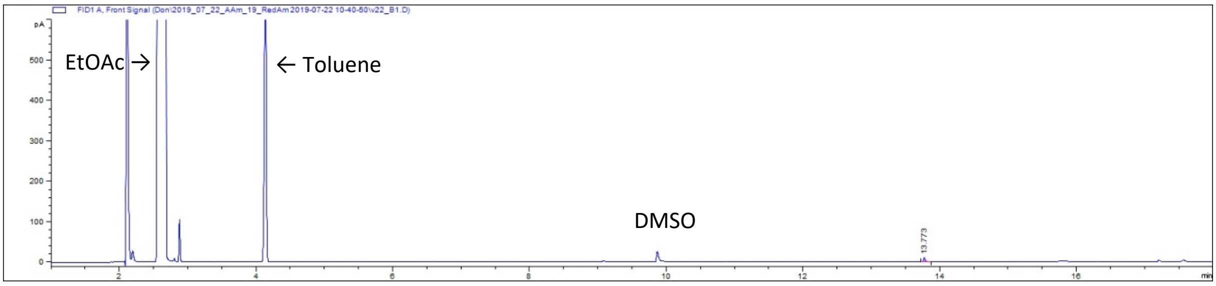

Reference compounds **10b** and **10c**

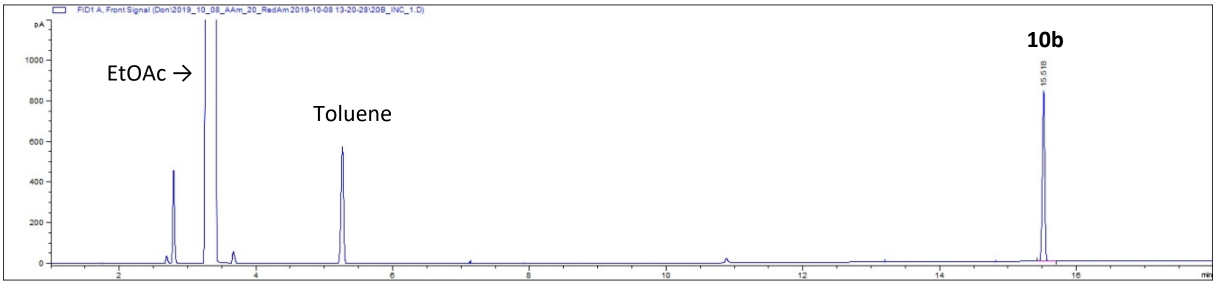

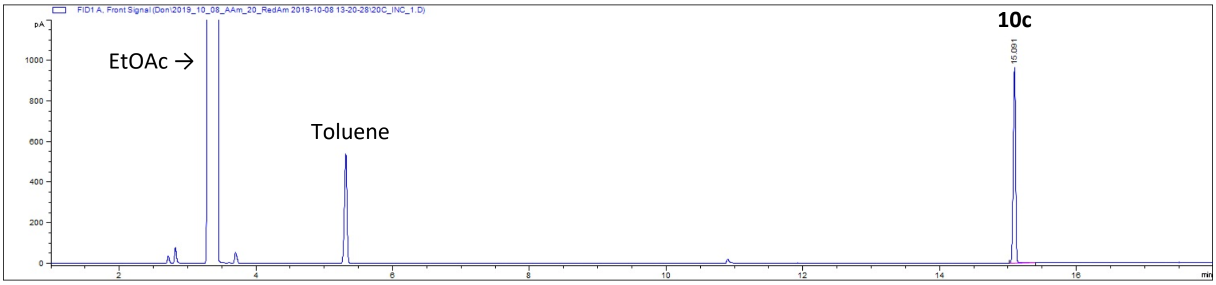

Biocatalytic reductive amination of **10b** catalyzed by LE-AmDH-v22

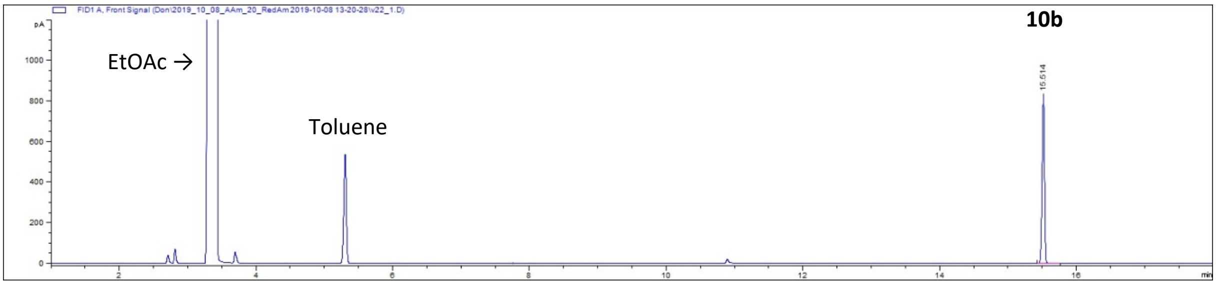

Reference compounds **11b** and **11c**

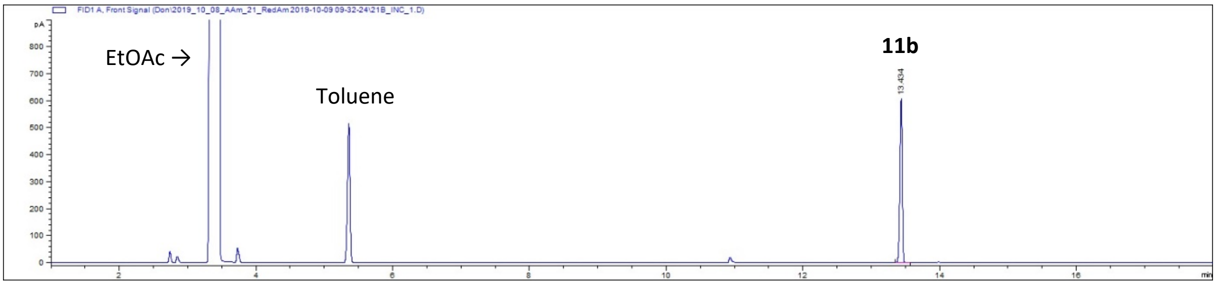

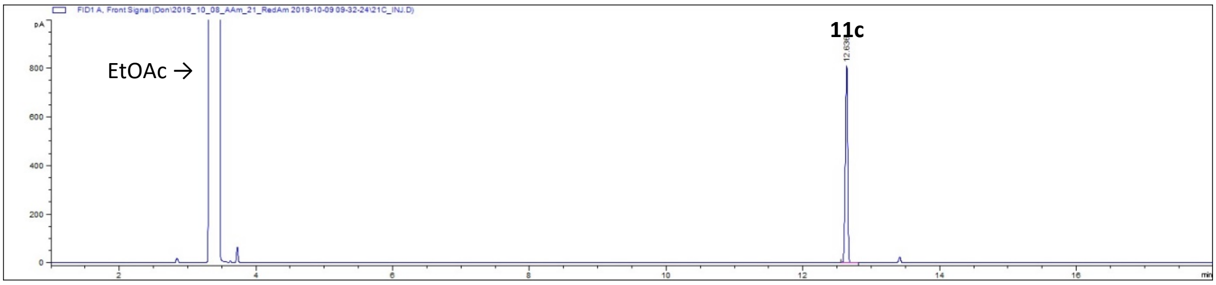

Biocatalytic reductive amination of **11b** catalyzed by LE-AmDH-v22

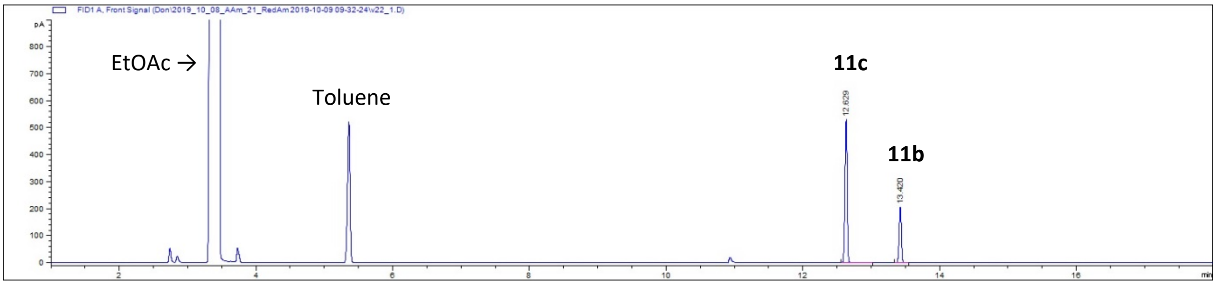

Reference compounds **12b** and **12c**

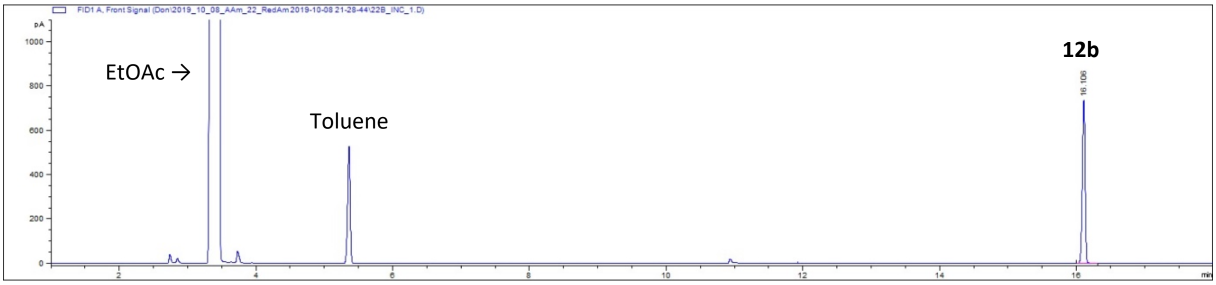

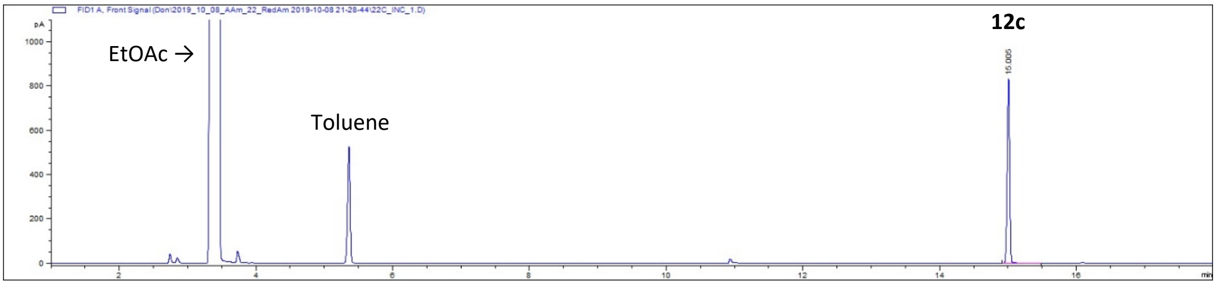

Biocatalytic reductive amination of **12b** catalyzed by LE-AmDH-v22

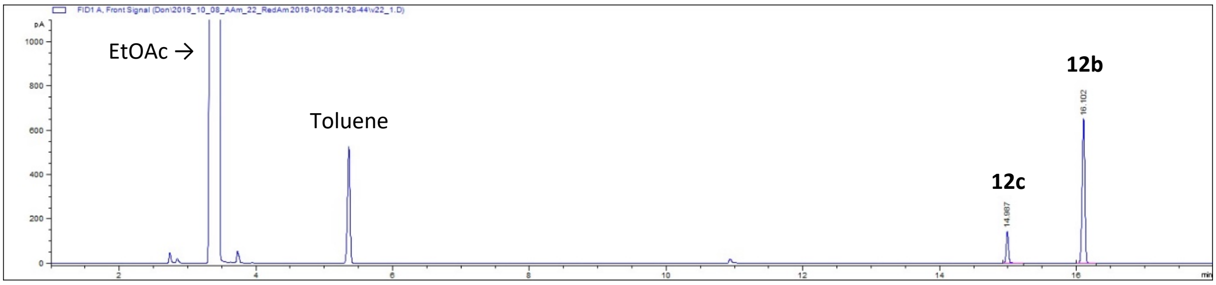

Reference compounds **13b** and **13c**

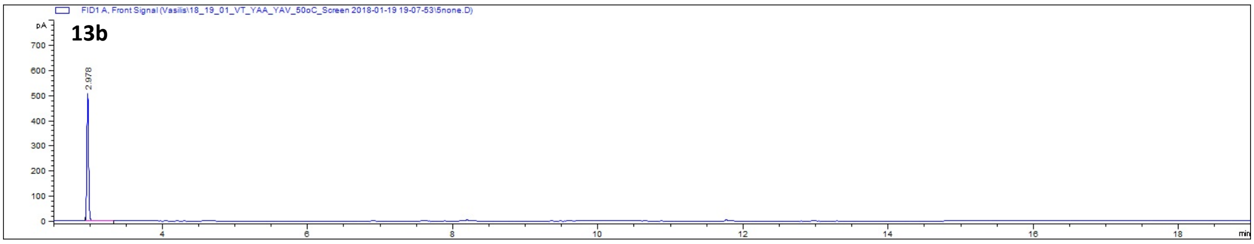

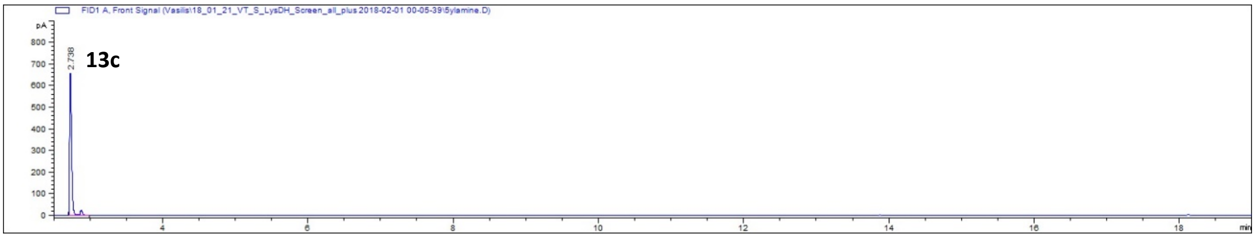

Biocatalytic reductive amination of **13b** catalyzed by LE-AmDH-v22

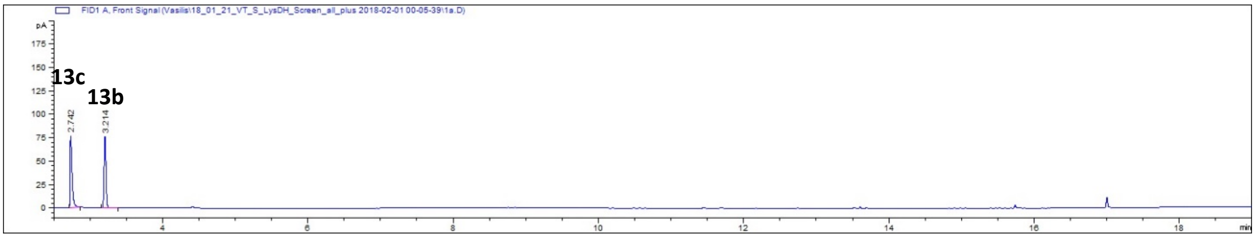

Reference compounds **14b** and **14c**

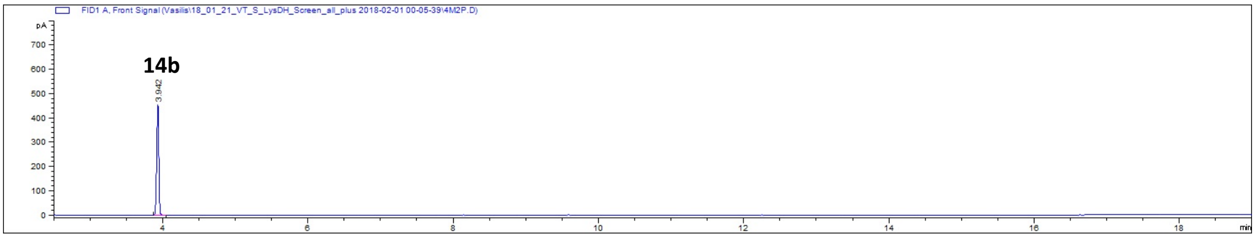

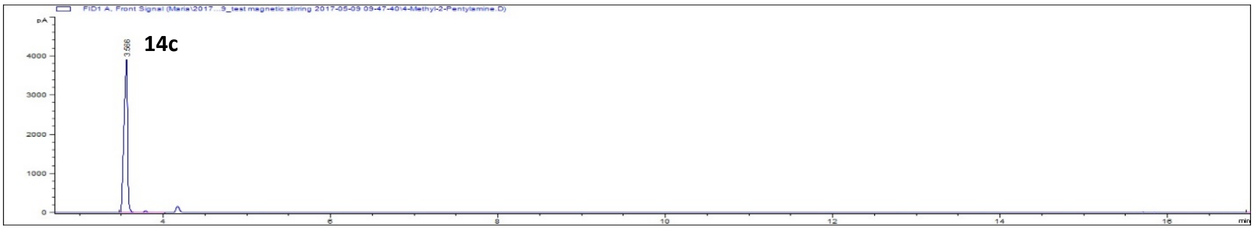

Biocatalytic reductive amination of **14b** catalyzed by LE-AmDH-v22

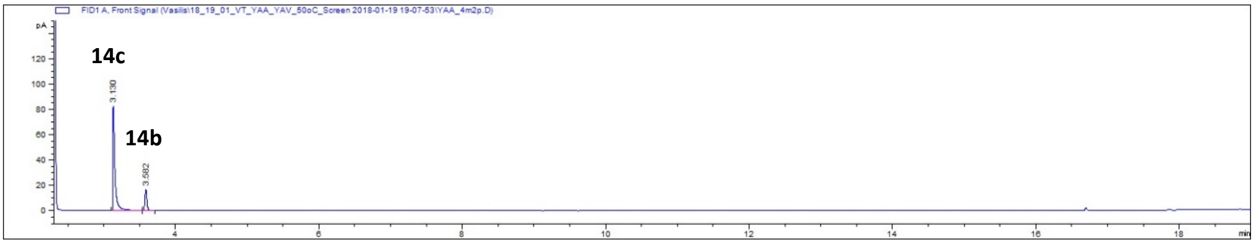

Reference compounds **15b** and **15c**

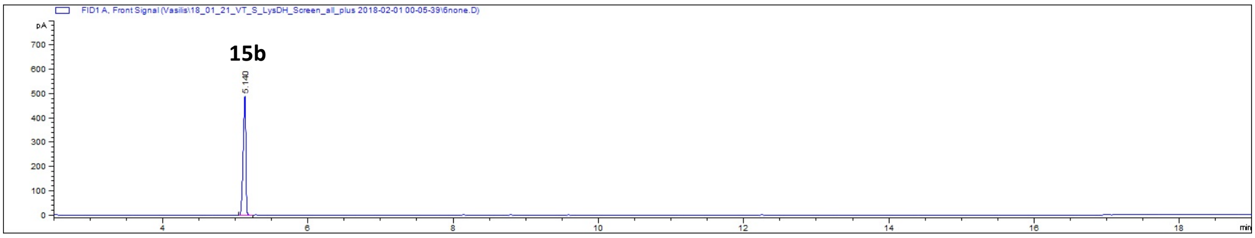

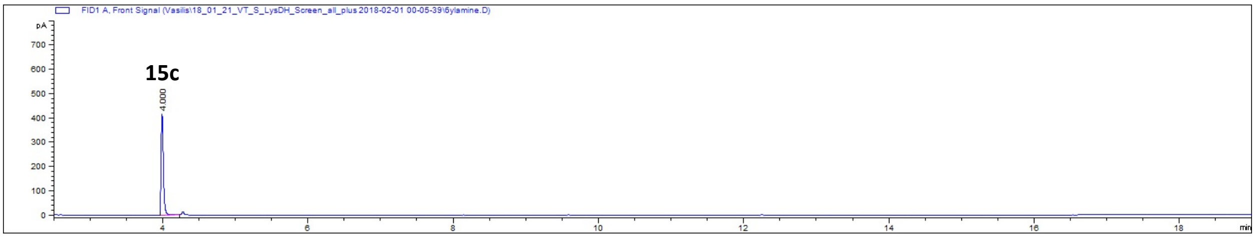

Biocatalytic reductive amination of **15b** catalyzed by LE-AmDH-v22

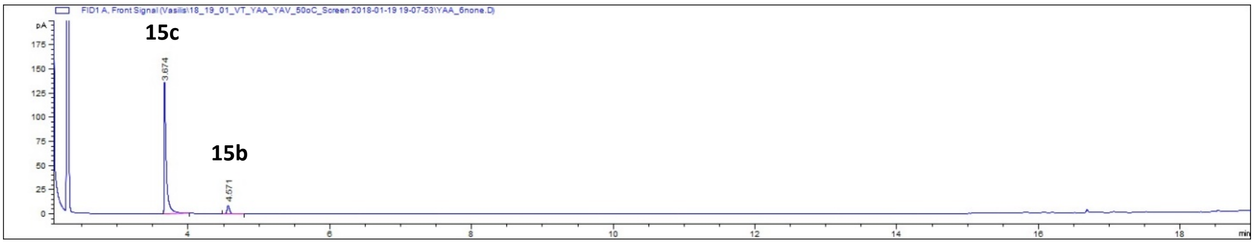

Reference compounds **16b** and **16c**

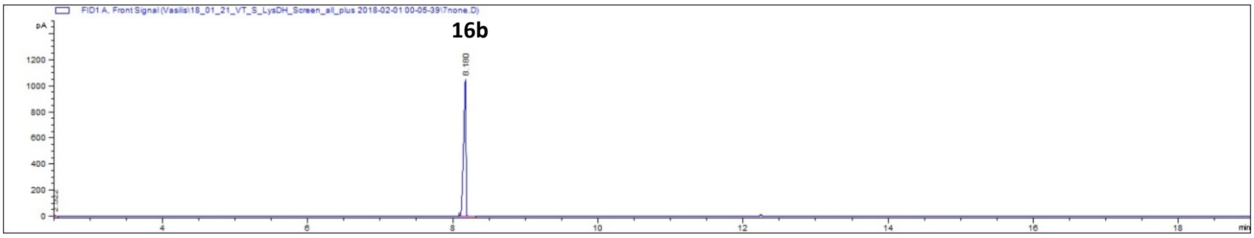

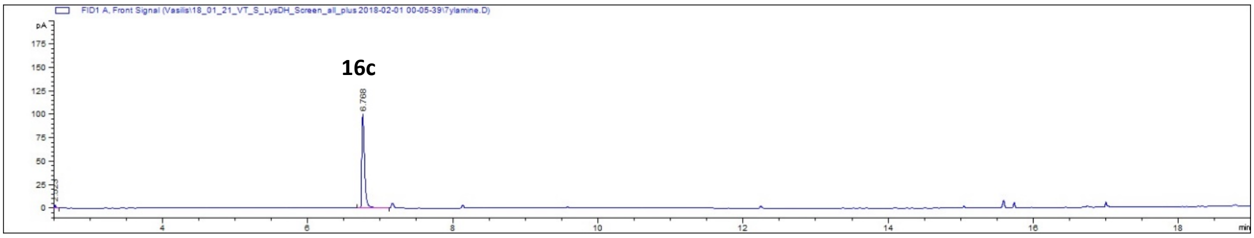

Biocatalytic reductive amination of **16b** catalyzed by LE-AmDH-v25

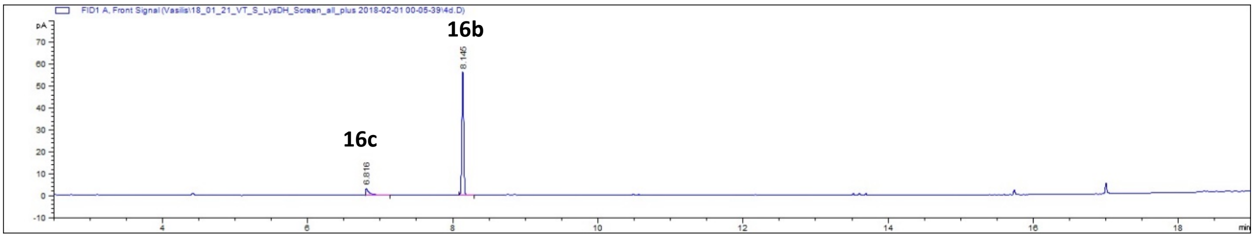

Reference compounds **17b** and **17c**

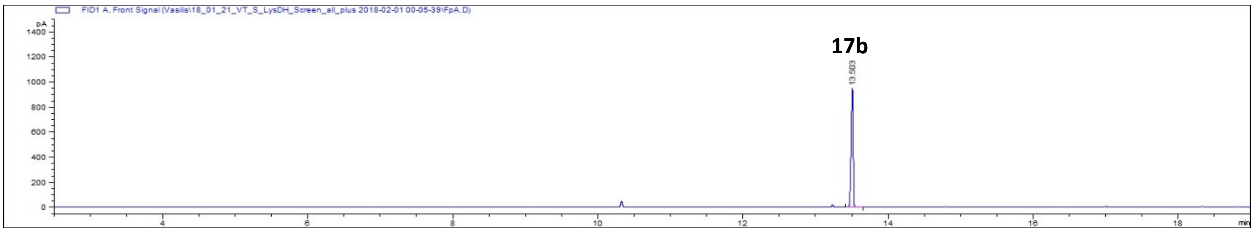

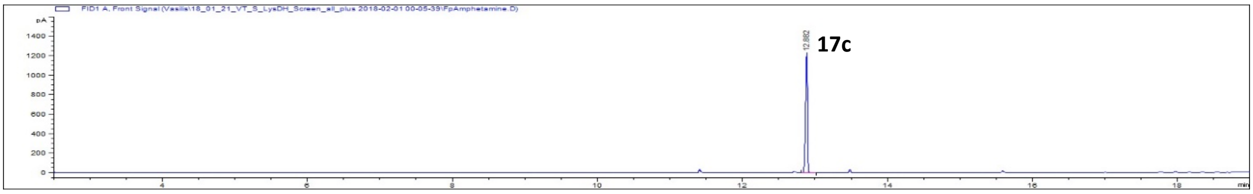

Biocatalytic reductive amination of **17b** catalyzed by LE-AmDH-v25

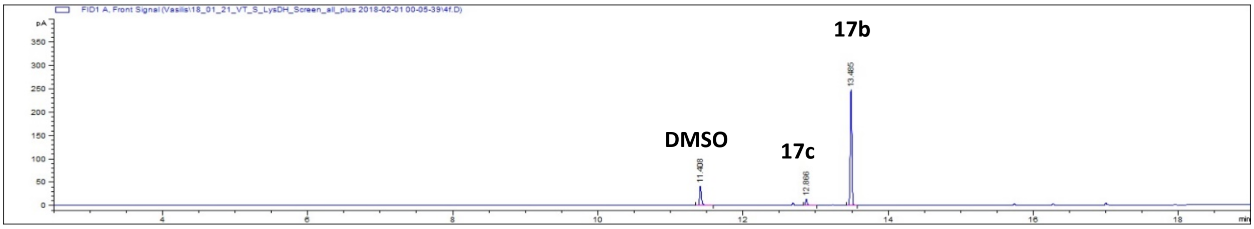

Reference compounds **18b** and **18c**

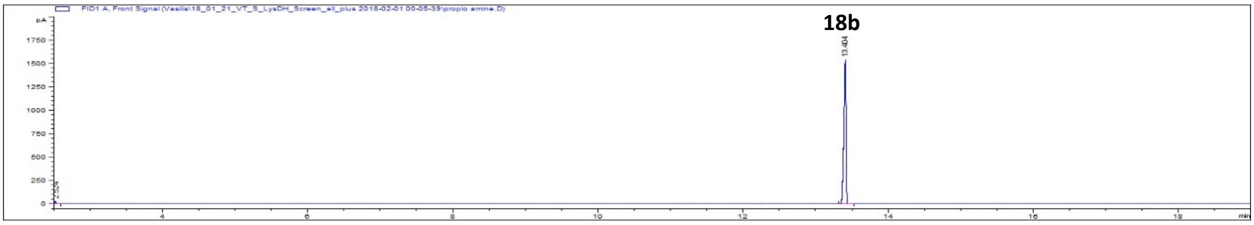

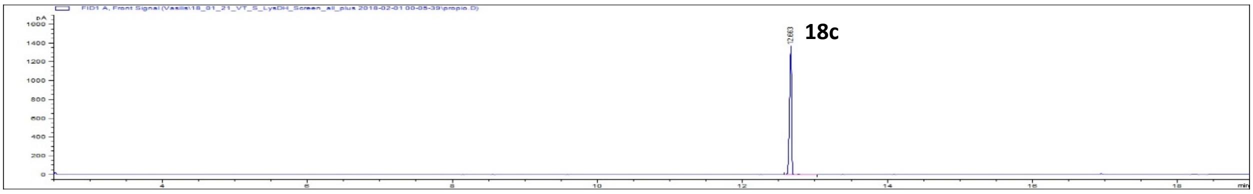

Biocatalytic reductive amination of **18b** catalyzed by LE-AmDH-v22

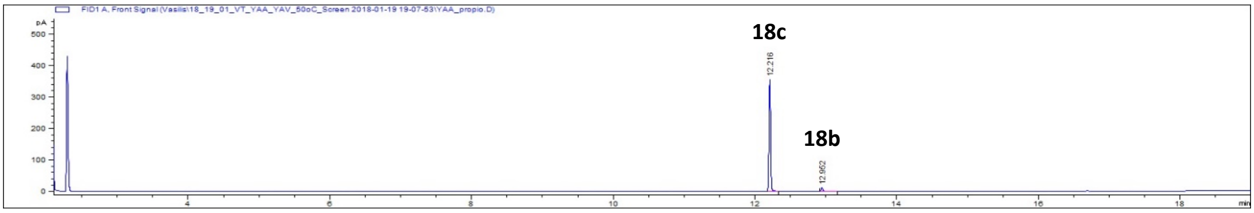

Reference compounds **19b** and **19c**

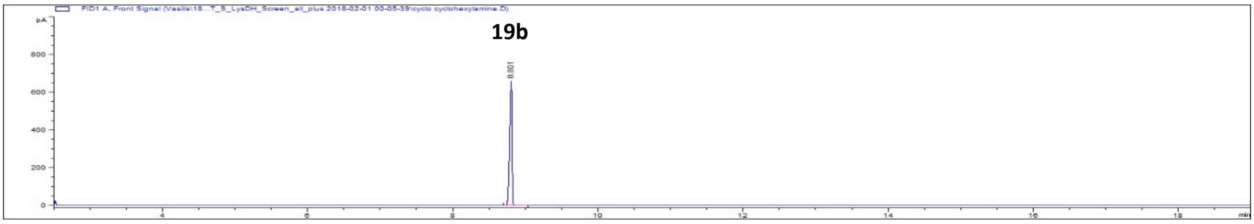

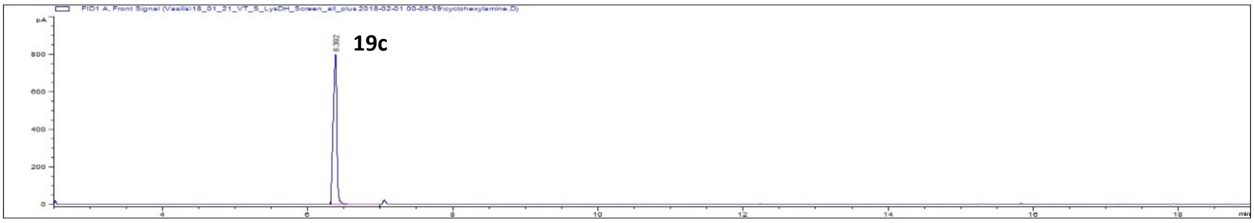

Biocatalytic reductive amination of **19b** catalyzed by LE-AmDH-v22

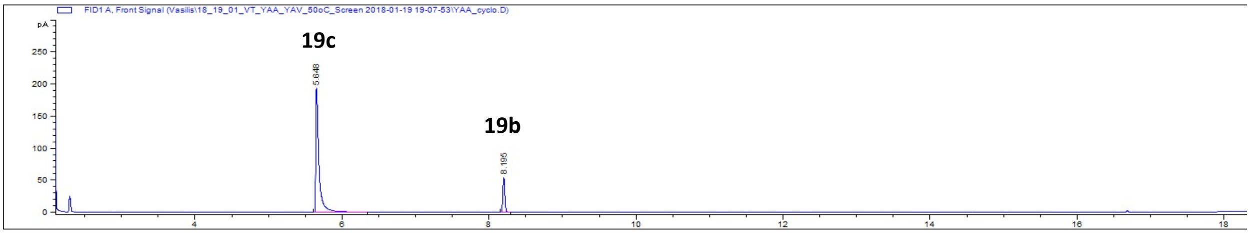

Reference compound **20b**

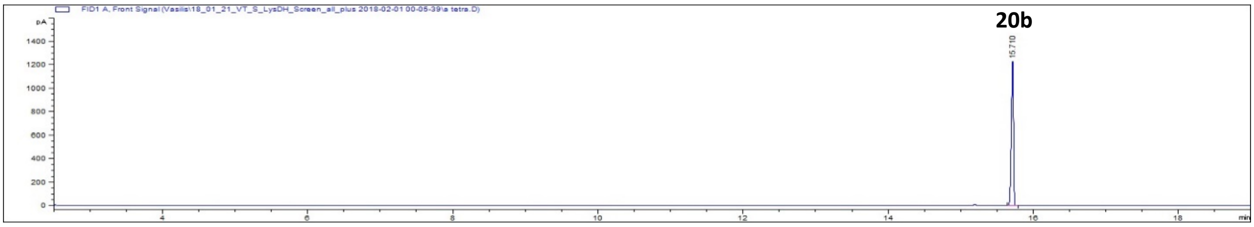

Reference compound **20c** synthetized by transaminase Armut11-ωTA

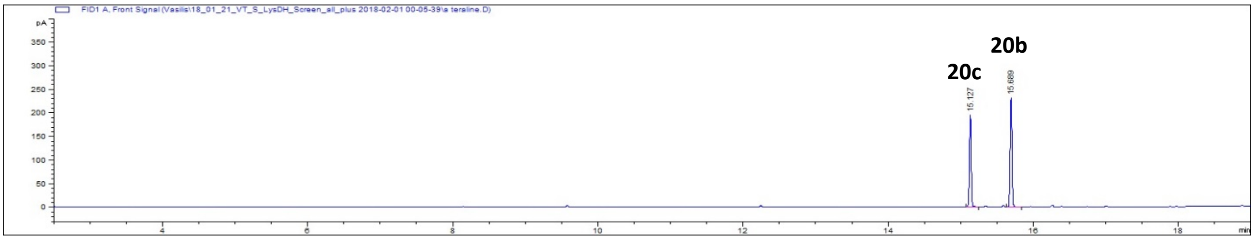

Biocatalytic reductive amination of **20b** catalyzed by LE-AmDH-v22

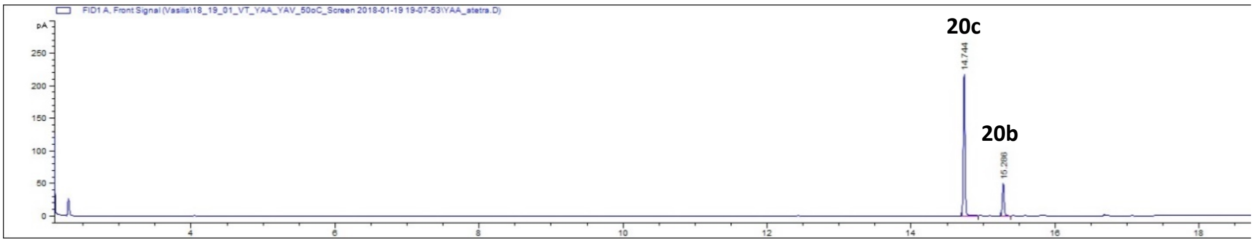

Reference compound **21b**

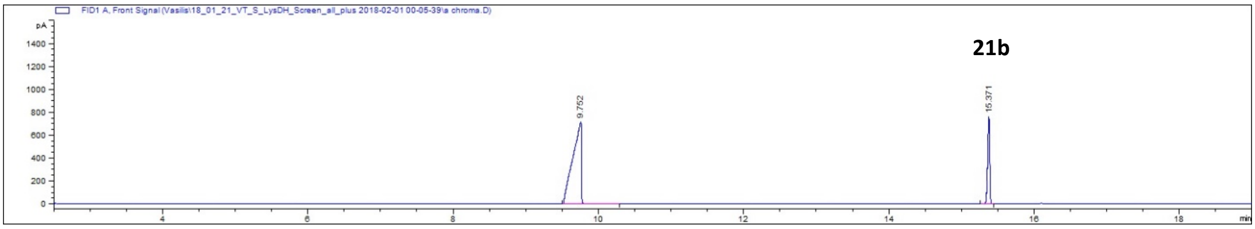

Reference compound **21c** synthetized by transaminase Ar(*R*)mut11-ωTA

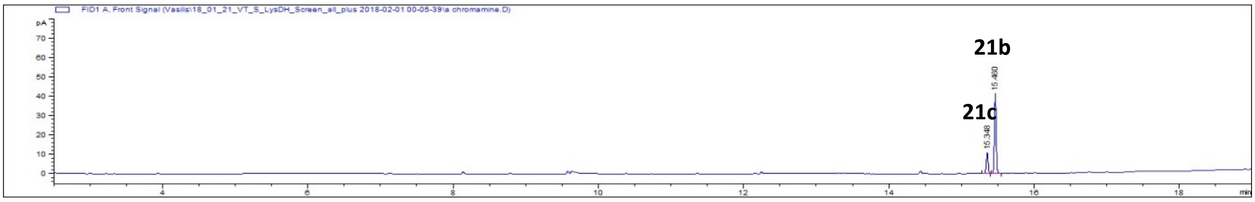

Biocatalytic reductive amination of **21b** catalyzed by LE-AmDH-v22

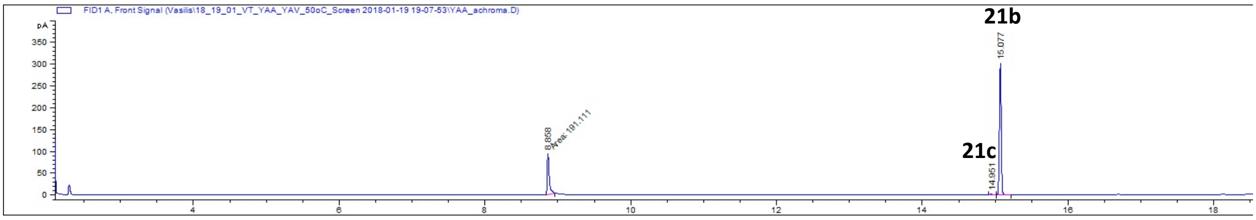

10.2 GC-FID chromatograms for determination of the absolute configuration of the amines

Reference compound (*R*)-configured **11c** (derivatized as acetamido)

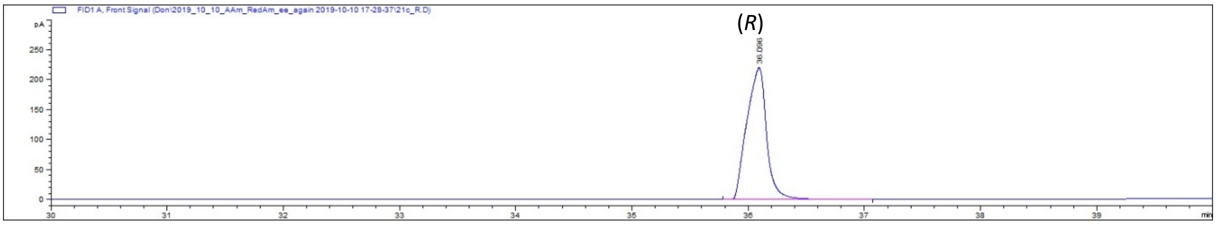

Reference compound (*S*)-configured **11c** (derivatized as acetamido)

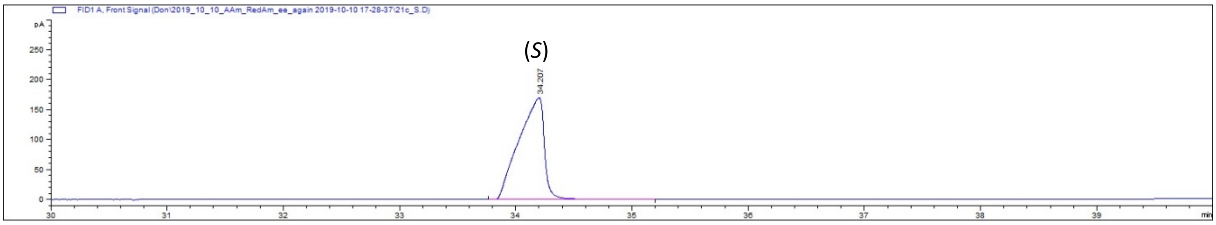

GC-FID chromatogram of **11c** obtained by reaction with LE-AmDH-v22 (derivatized as acetamido)

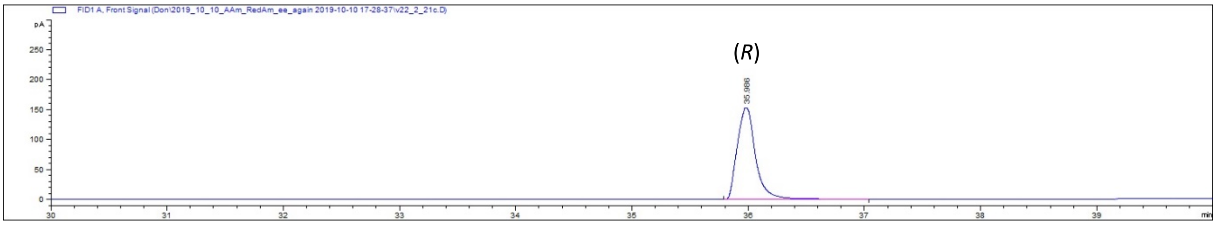

Reference compound *rac*-**12c** (derivatized as acetamido)

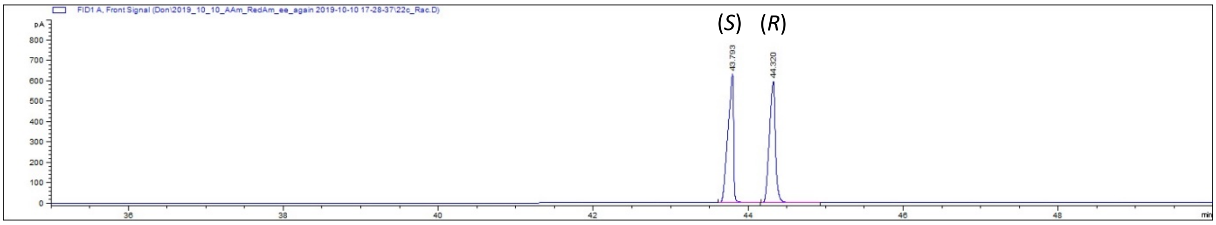

Reference compound (*R*)-configured **12c** (derivatized as acetamido)

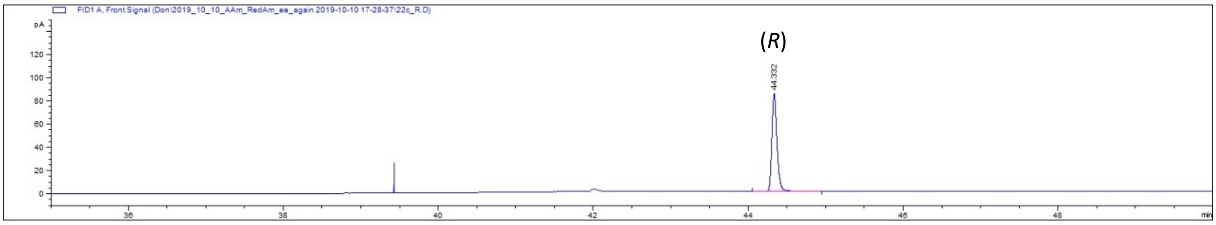

GC-FID chromatogram of **12b** obtained by reaction with LE-AmDH-v1 (derivatized as acetamido)

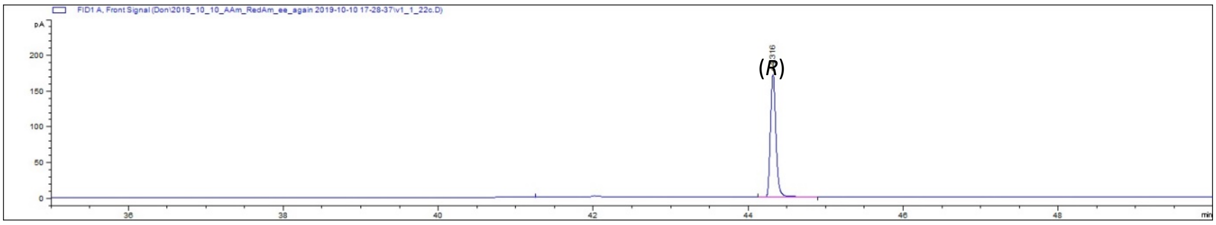

Reference compound *rac*-**13c** (derivatized as acetamido)

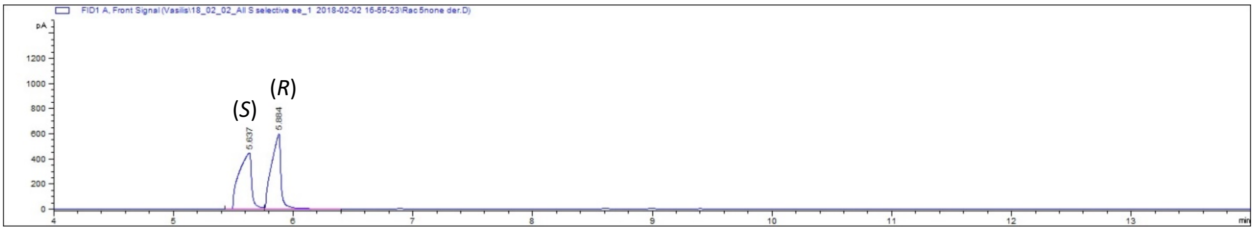

GC-FID chromatogram of **13c** obtained by reaction with LE-AmDH-v22 (derivatized as acetamido)

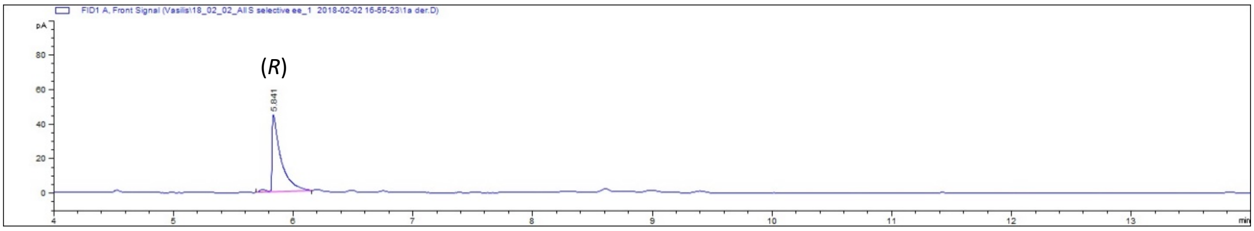

Reference compound (*R*)-configured **14c** (derivatized as acetamido)

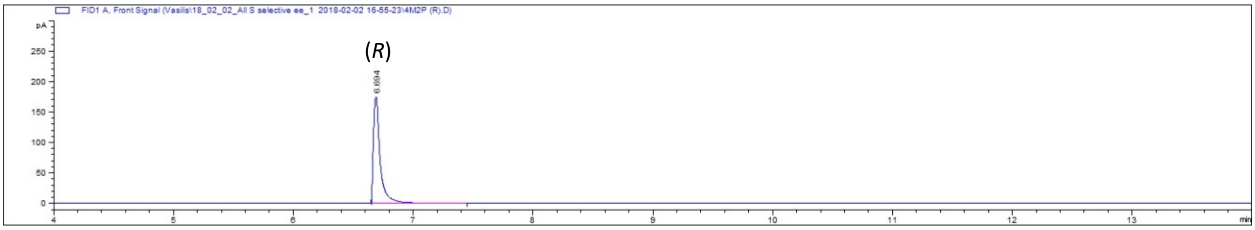

Reference compound (*S*)-configured **14c** (derivatized as acetamido)

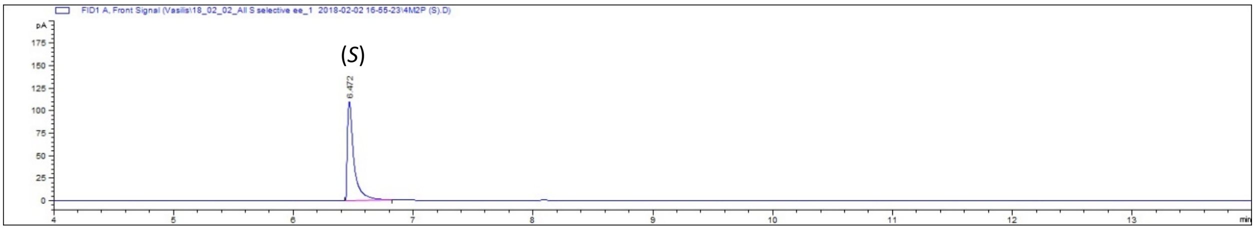

GC-FID chromatogram of **14c** obtained by reaction with LE-AmDH-v22 (derivatized as acetamido)

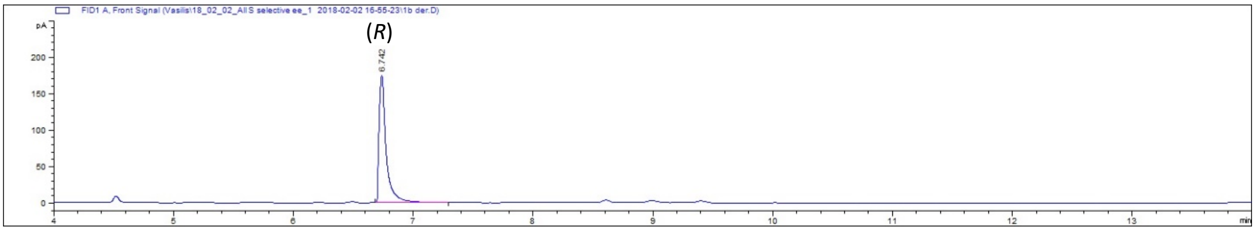

Reference compound (*S*)-configured **15c**

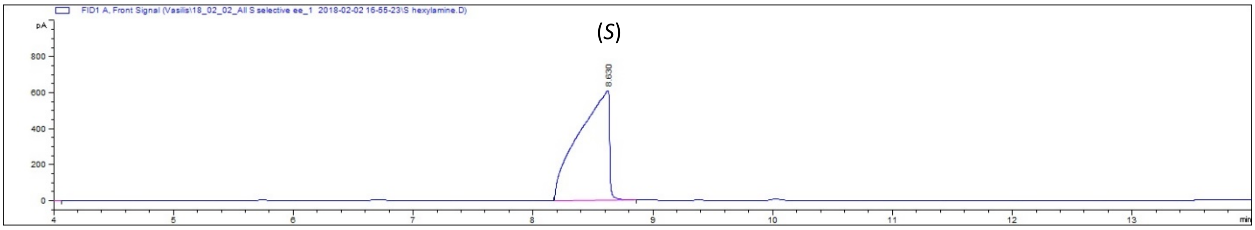

Reference compound (*R*)-configured **15c**

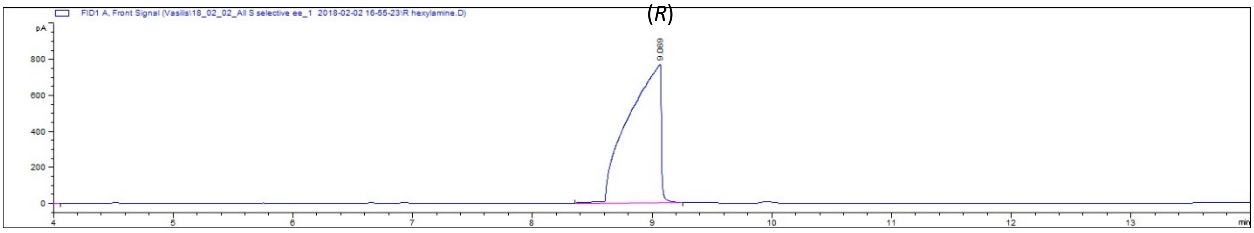

GC-FID chromatogram of **15c** obtained by reaction with LE-AmDH-v22 (derivatized as acetamido)

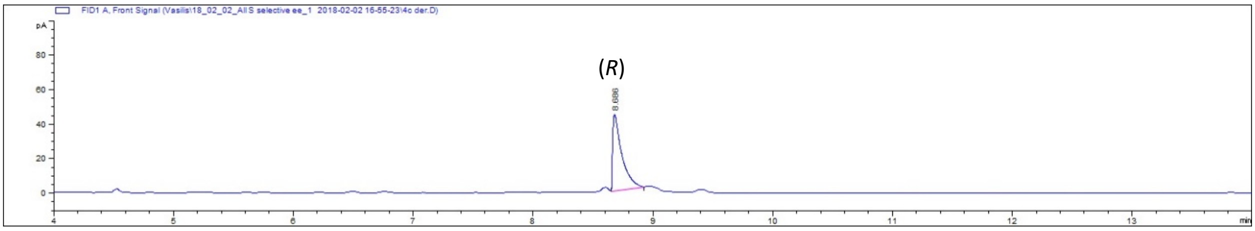

Reference compound (*S*)-configured **18c**

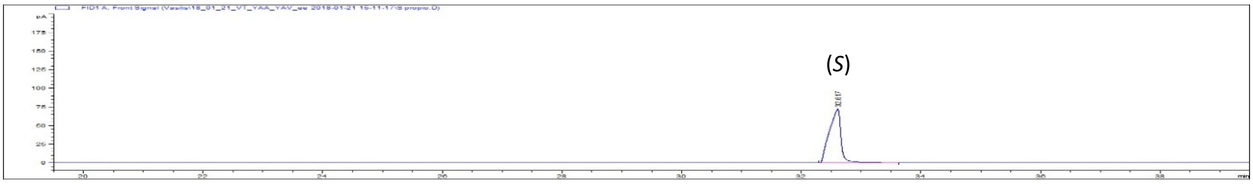

Reference compound (*R*)-configured **18c**

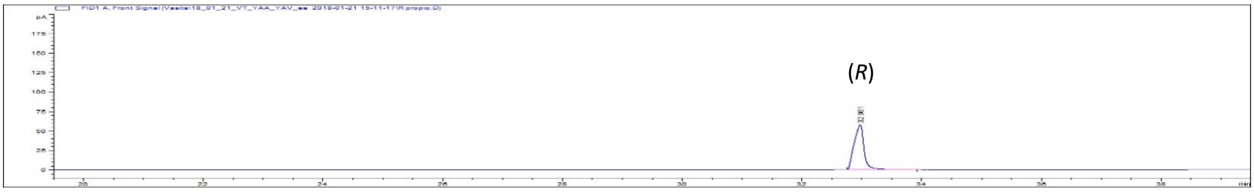

GC-FID chromatogram of **18c** obtained by reaction with LE-AmDH-v22 (derivatized as acetamido)

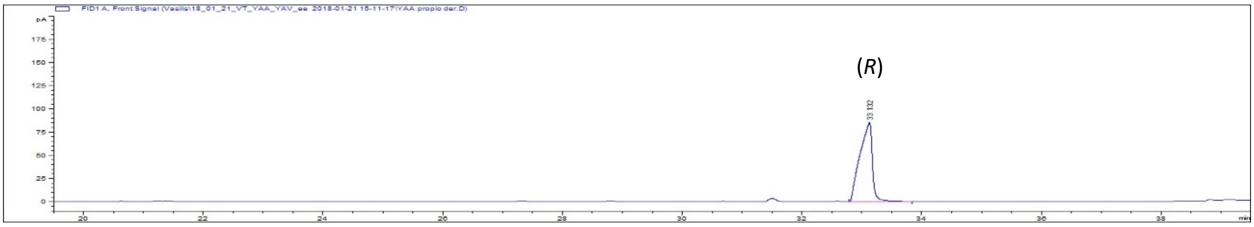

Reference compound (*S*)-configured **20c**

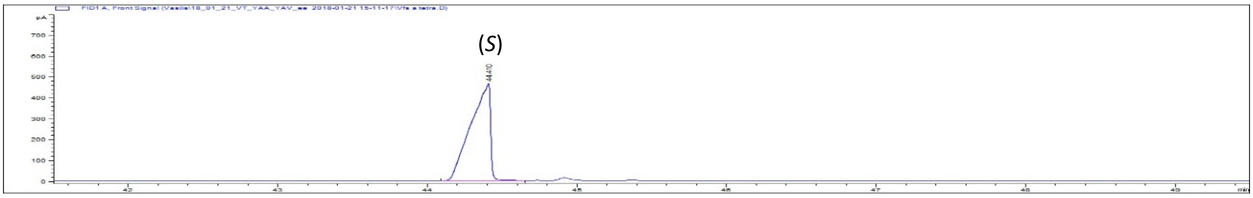

Reference compound (*R*)-configured **20c**

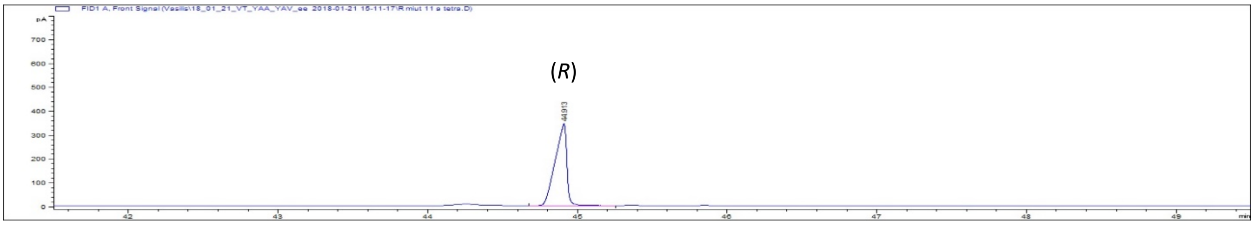

GC-FID chromatogram of **20c** obtained by reaction with LE-AmDH-v22 (derivatized as acetamido)

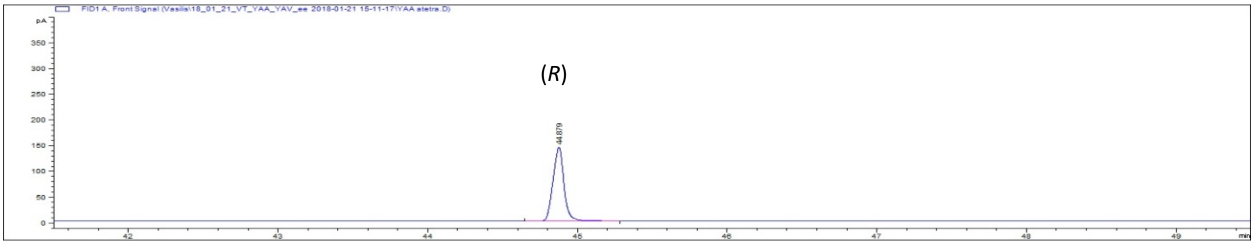

Reference compound (*S*)-configured **21c**

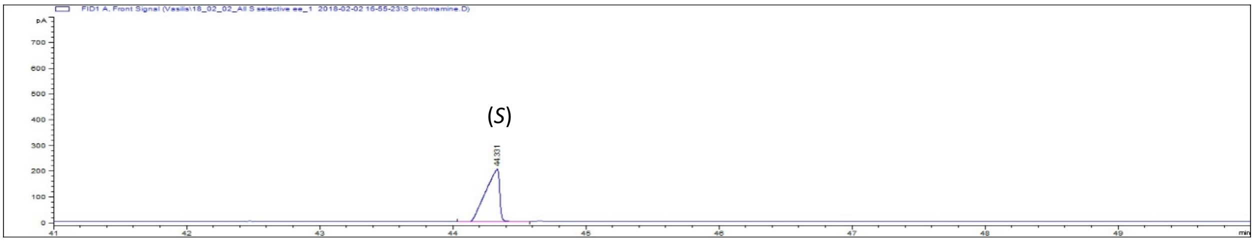

Reference compound (*R*)-configured **21c**

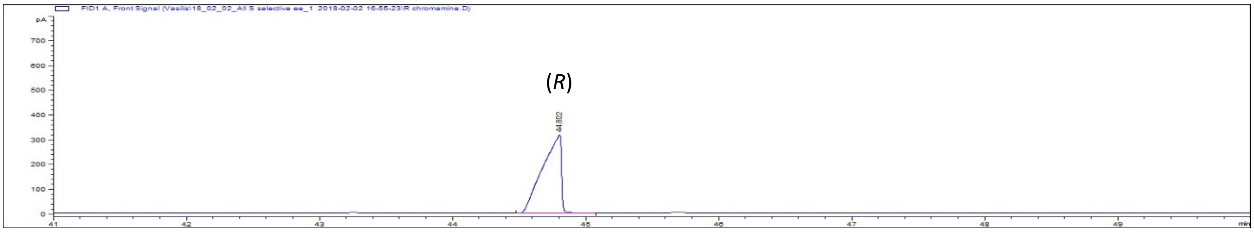

GC-FID chromatogram of **21c** obtained by reaction with LE-AmDH-v22 (derivatized as acetamido)

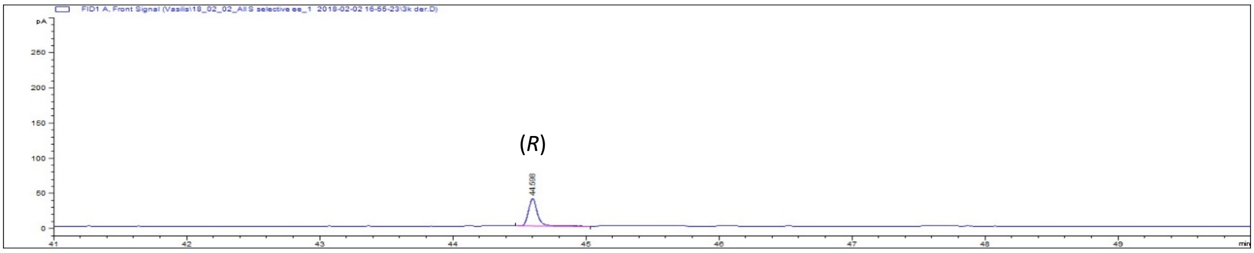

10.3 GC-FID chromatograms of reduction of aldehydes and ketones to alcohols

Reference compounds **1a** and **1b**

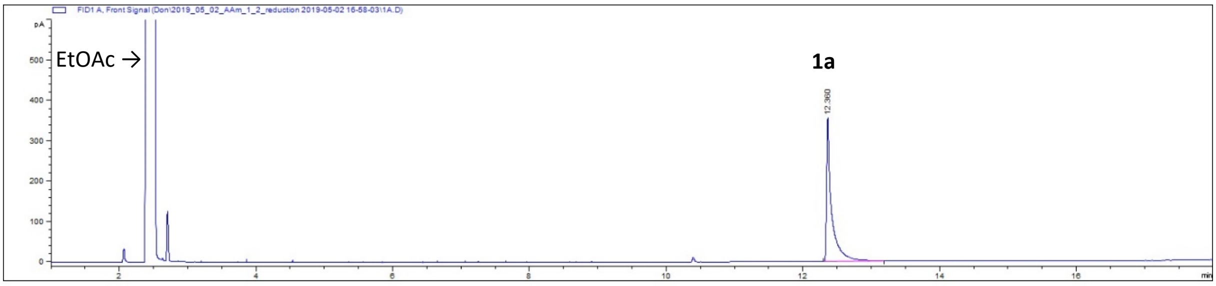

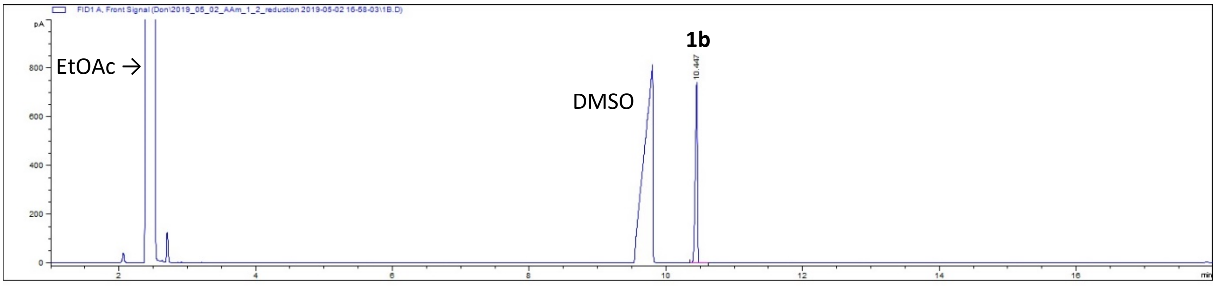

Biocatalytic reduction of **1b** catalyzed by LE-AmDH-v27

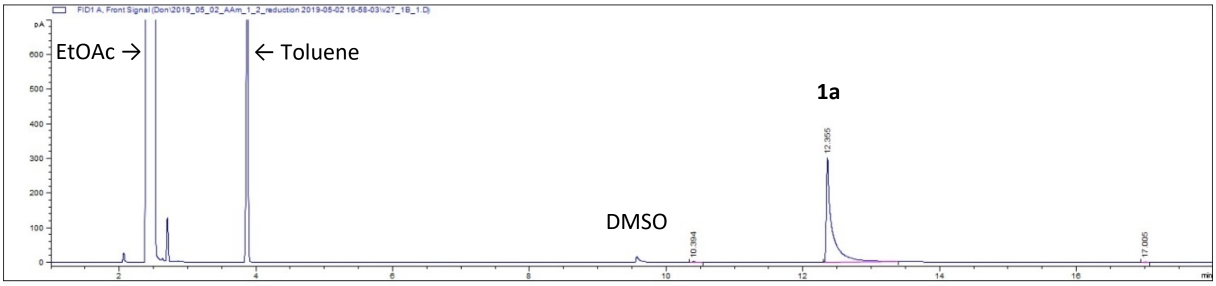

Reference compounds **2a** and **2b**

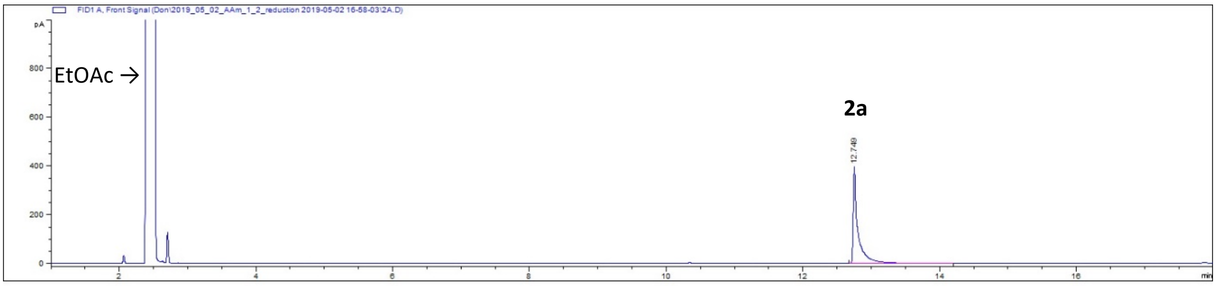

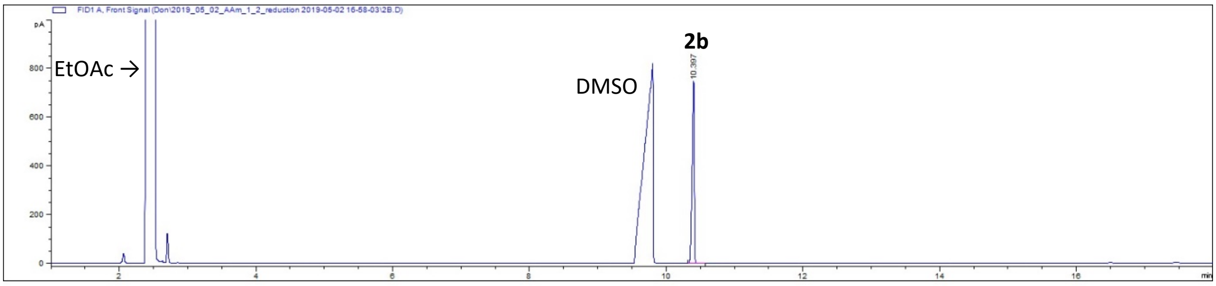

Biocatalytic reduction of **2b** catalyzed by LE-AmDH-v27

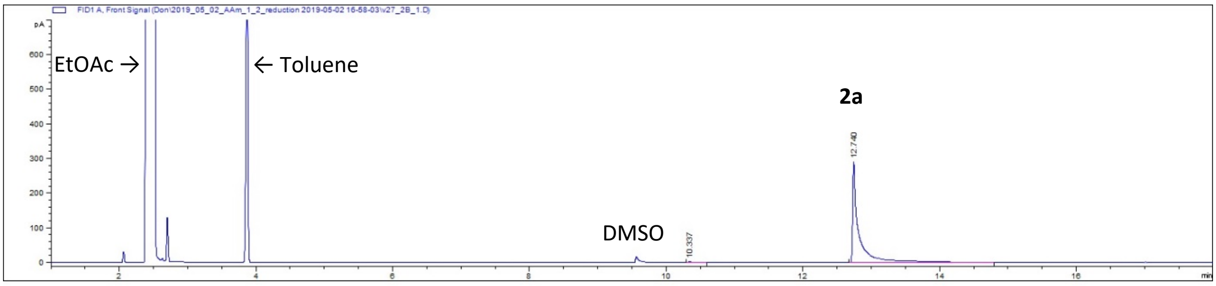

Reference compounds **3a** and **3b**

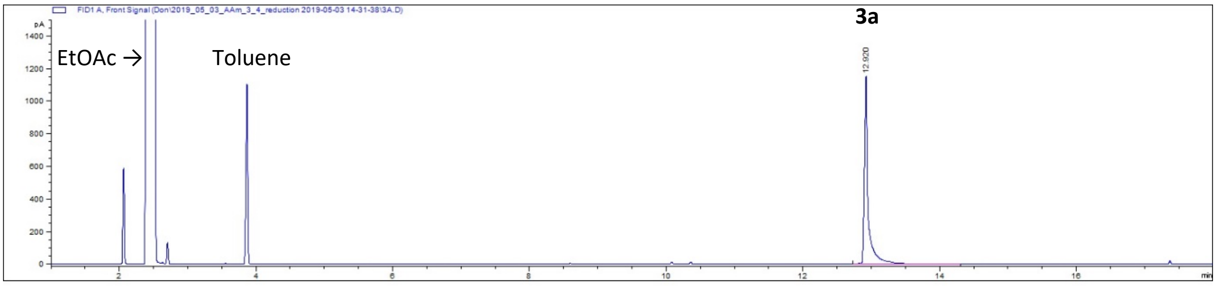

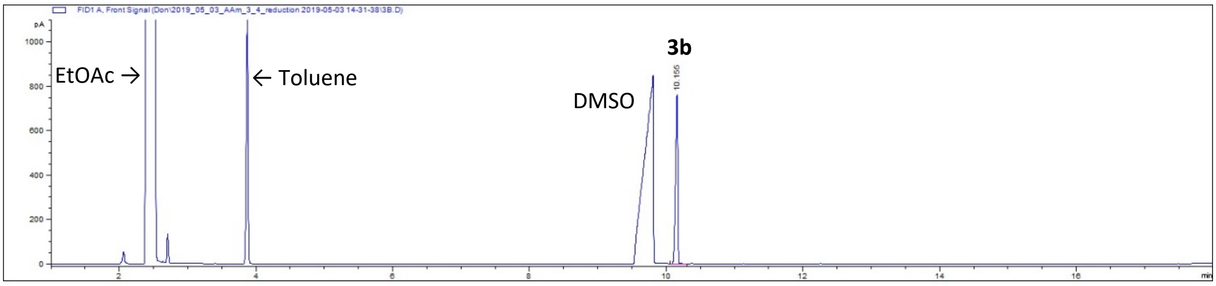

Biocatalytic reduction of **3b** catalyzed by LE-AmDH-v27

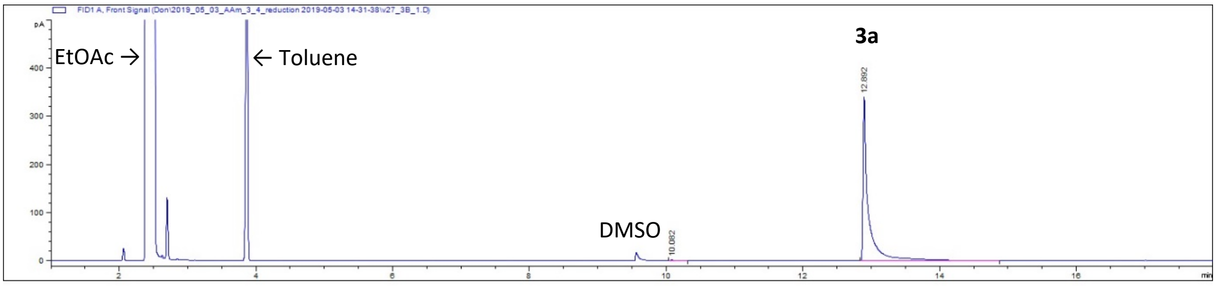

Reference compounds **4a** and **4b**

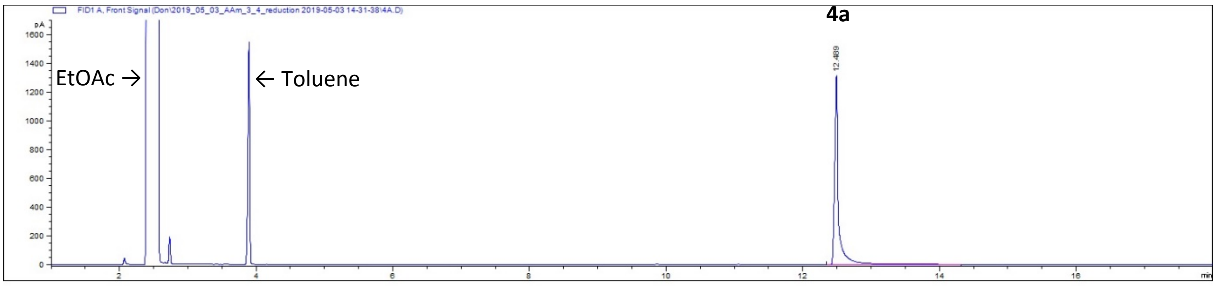

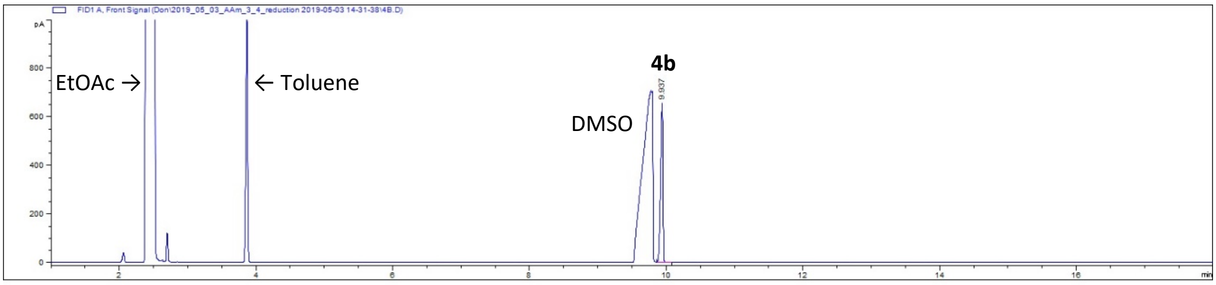

Biocatalytic reduction of **4b** catalyzed by LE-AmDH-v27

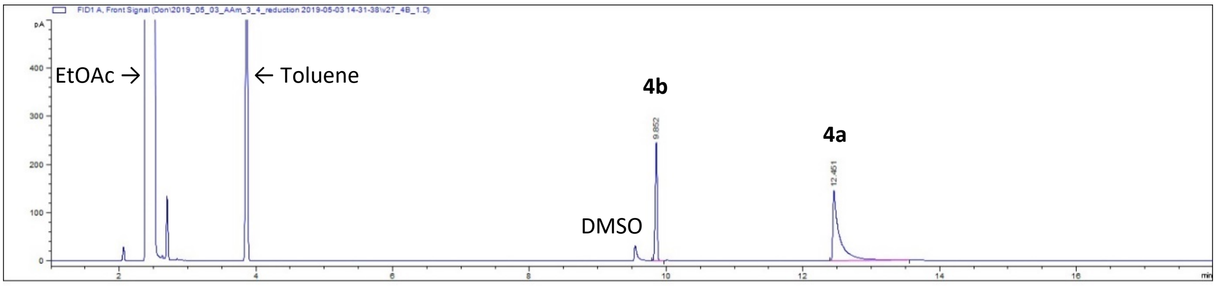

Reference compounds **5a** and **5b**

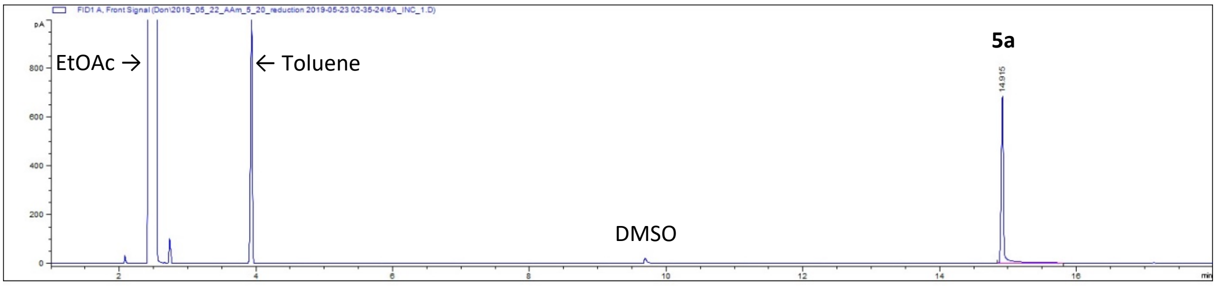

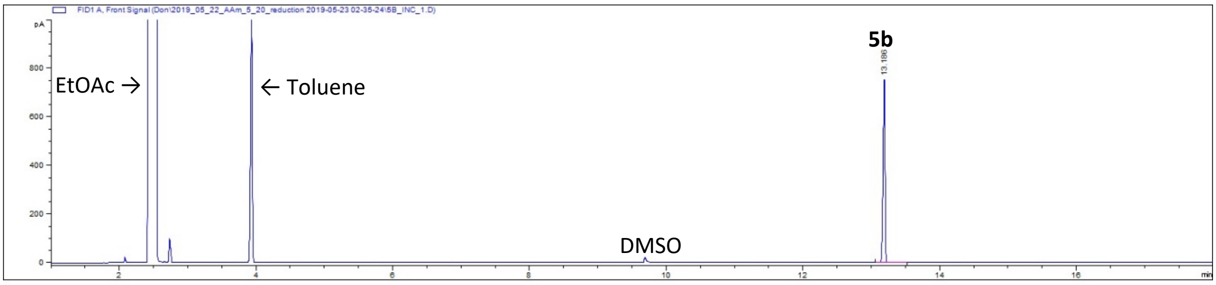

Biocatalytic reduction of **5b** catalyzed by LE-AmDH-v27

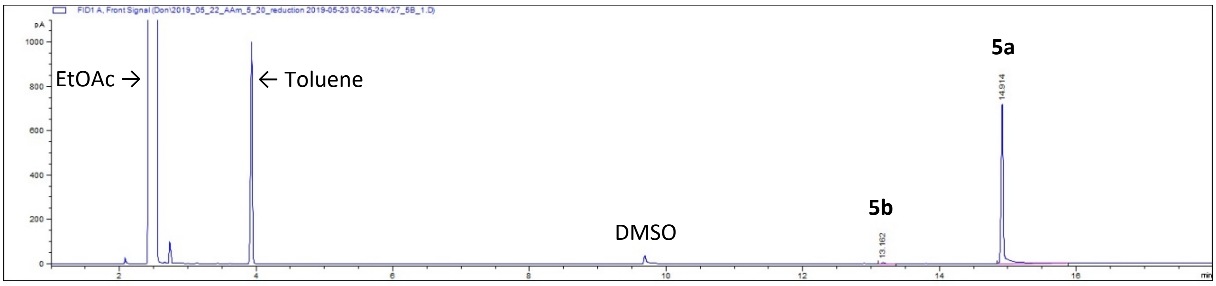

Reference compounds **6a** and **6b**

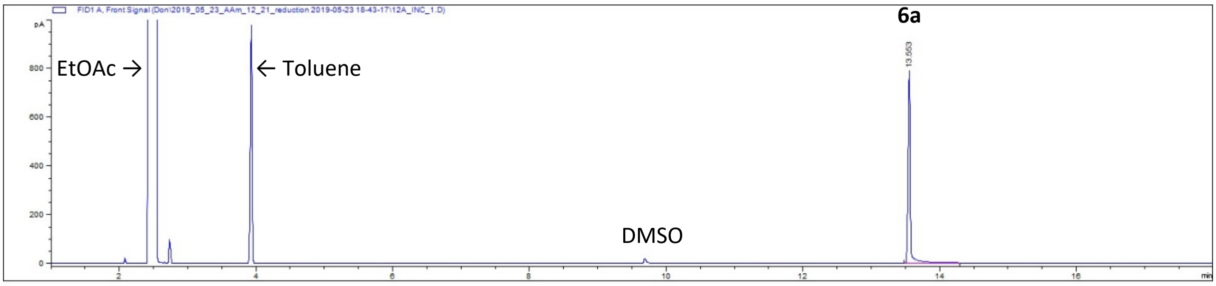

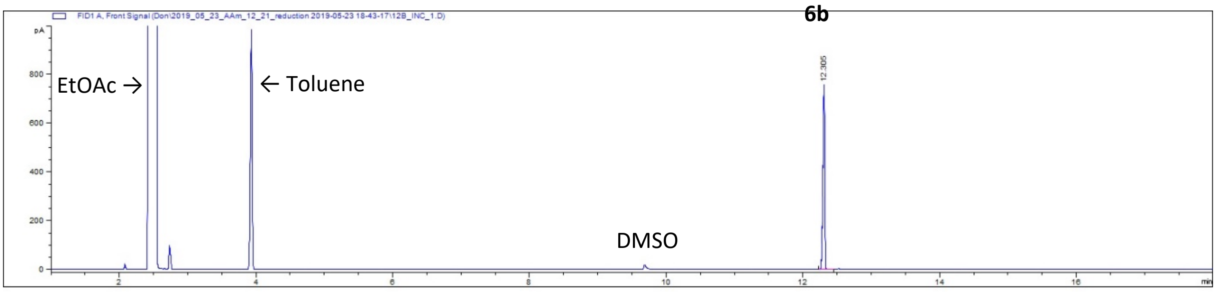

Biocatalytic reduction of **6b** catalyzed by LE-AmDH-v27

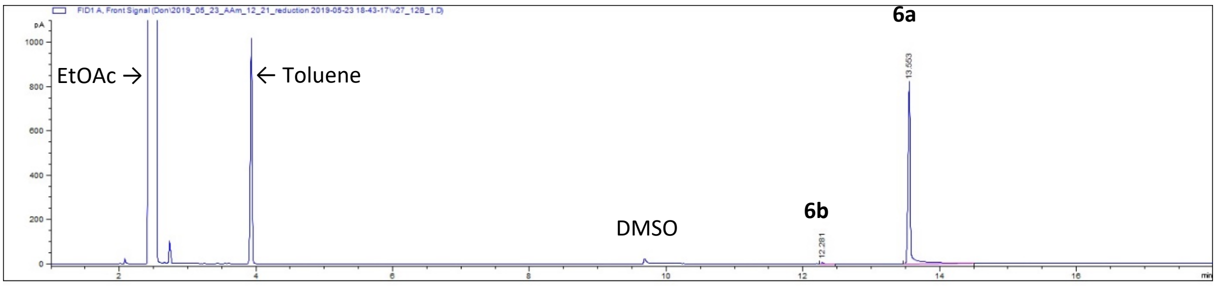

Reference compounds **7a** and **7b**

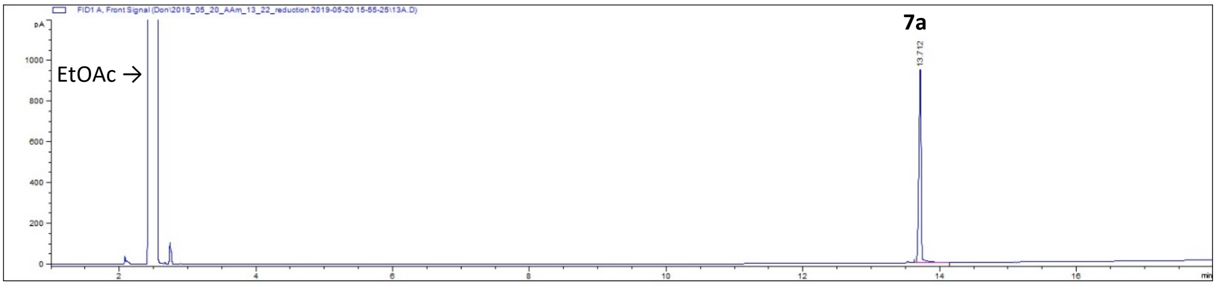

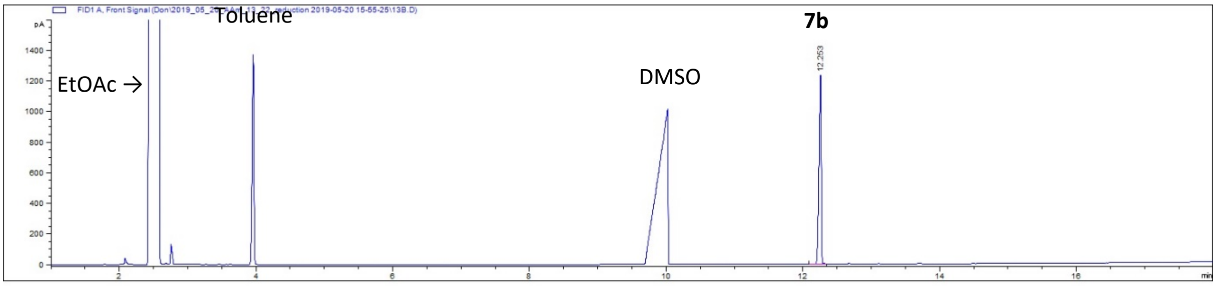

Biocatalytic reduction of **7b** using LE-AmDH-v27

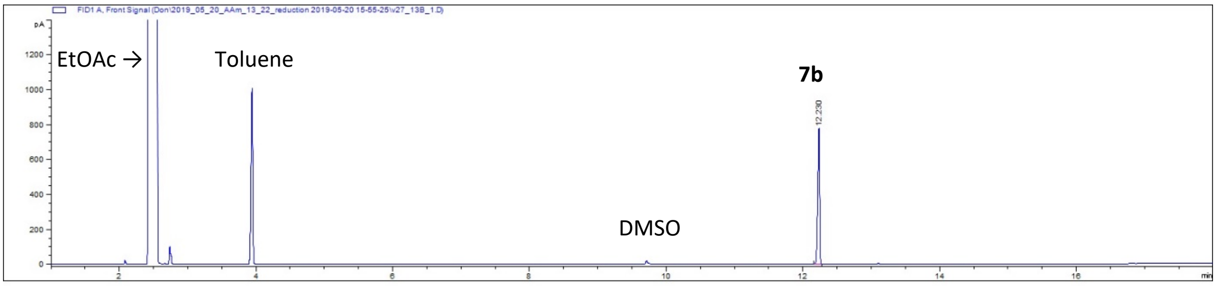

Reference compounds **8a** and **8b**

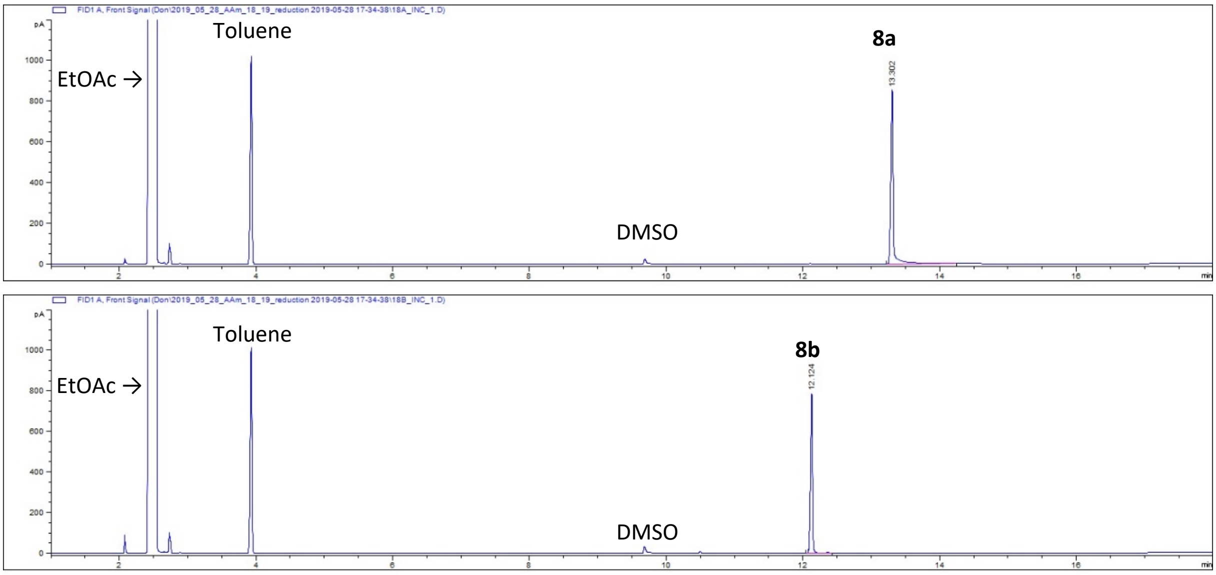

Biocatalytic reduction of **8b** catalyzed by LE-AmDH-v27

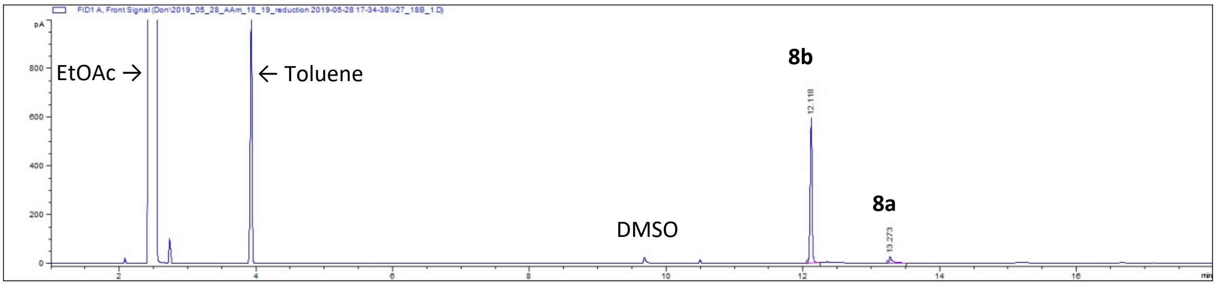

Reference compounds **9a** and **9b**

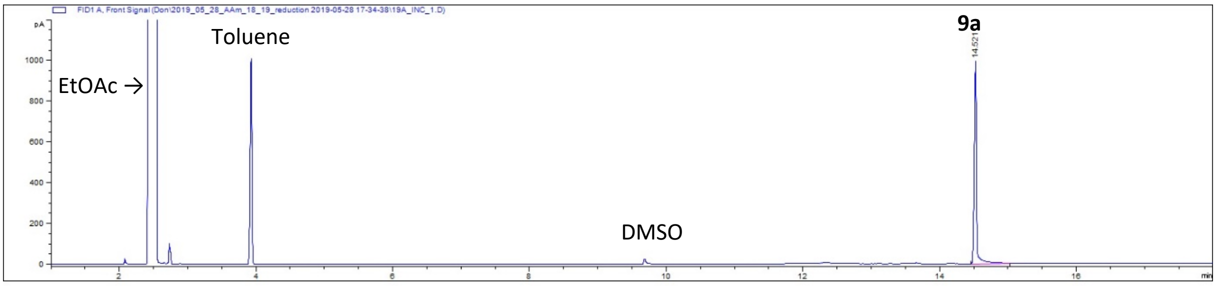

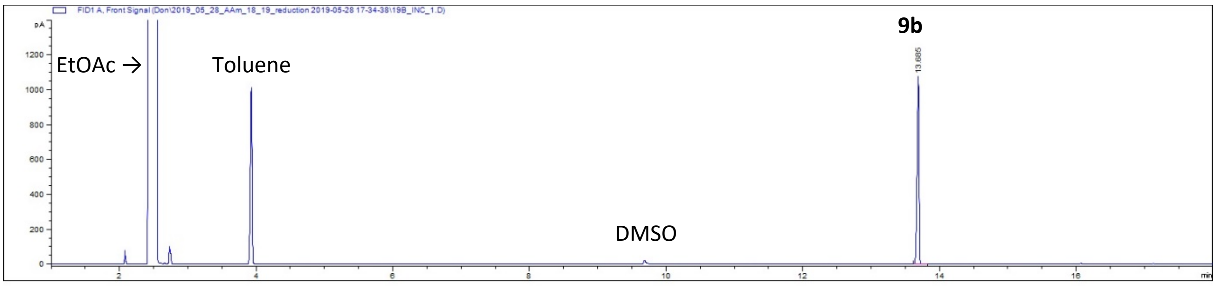

Biocatalytic reduction of **9b** catalyzed by LE-AmDH-v27

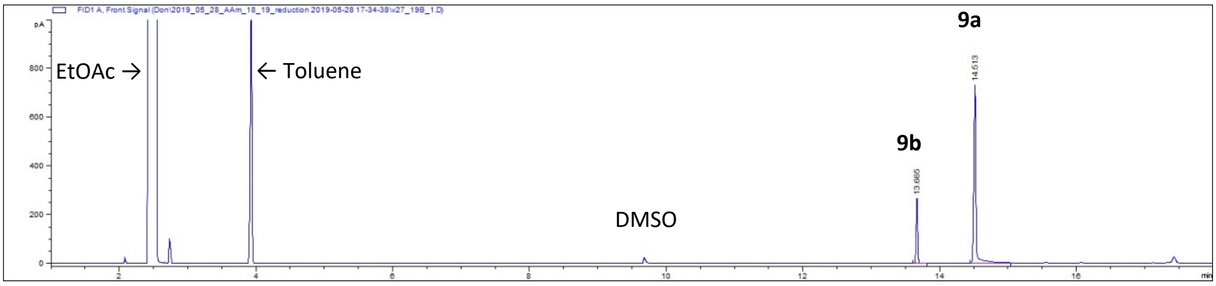

Reference compounds **10a** and **10b**

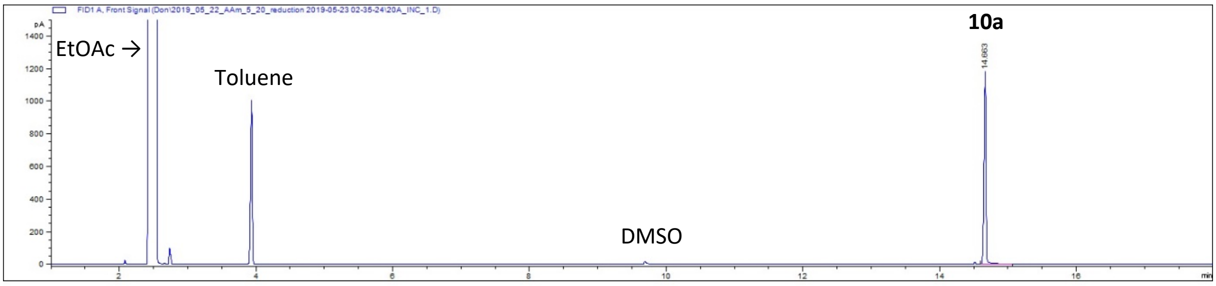

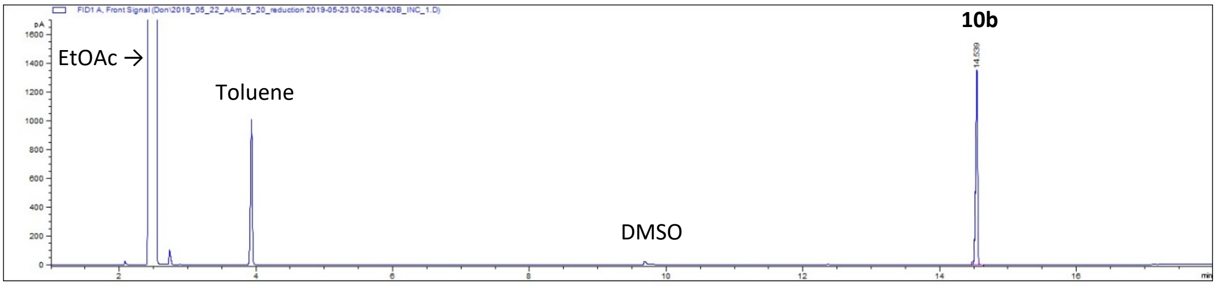

Biocatalytic reduction of **10b** catalyzed by LE-AmDH-v27

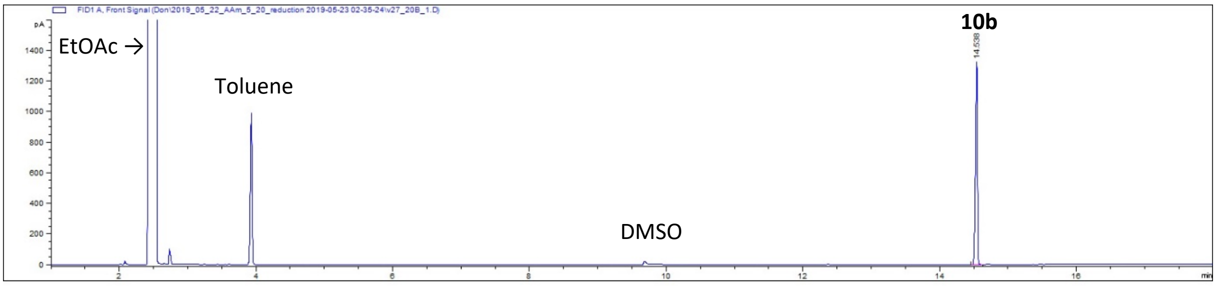

Reference compounds **11a** and **11b**

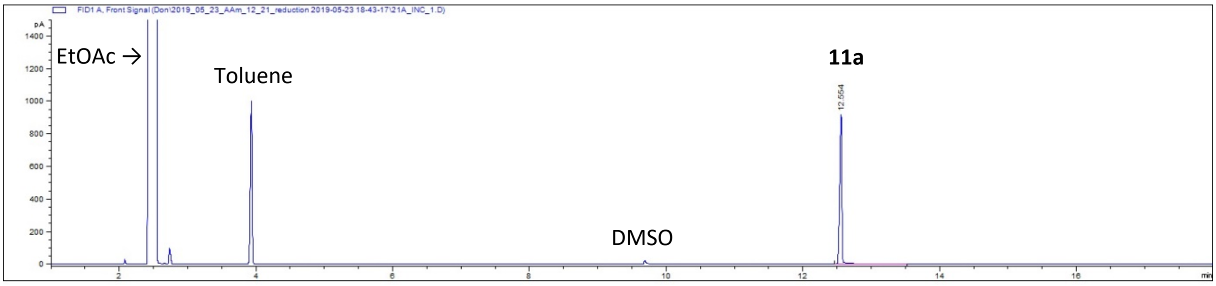

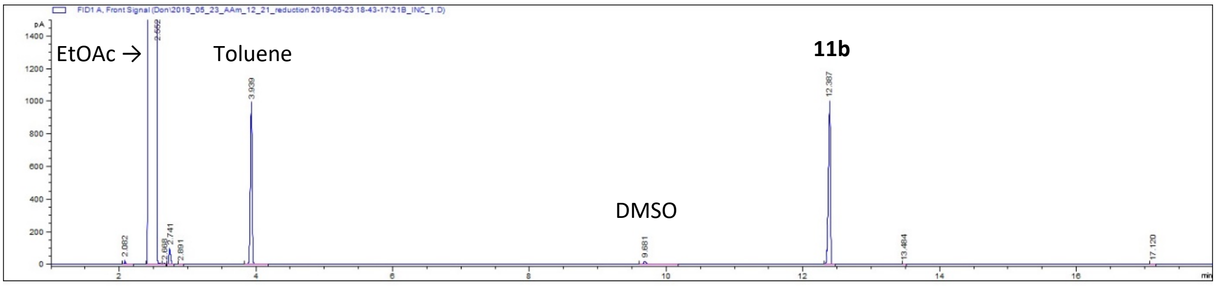

Biocatalytic reduction of **11b** catalyzed by LE-AmDH-v27

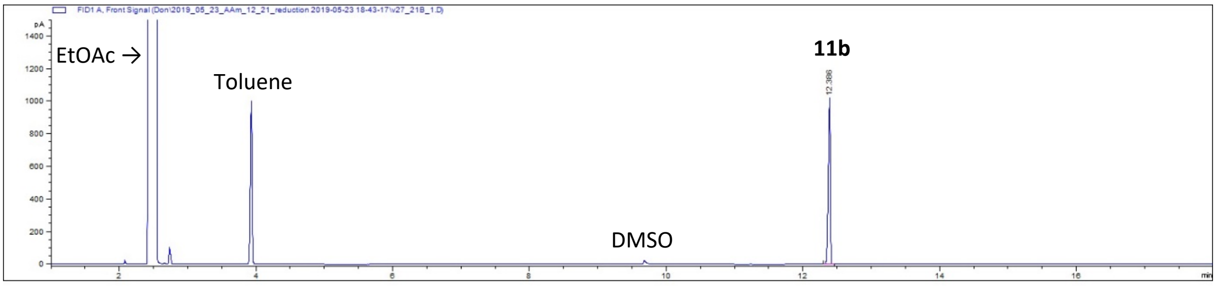

Reference compounds **12a** and **12b**

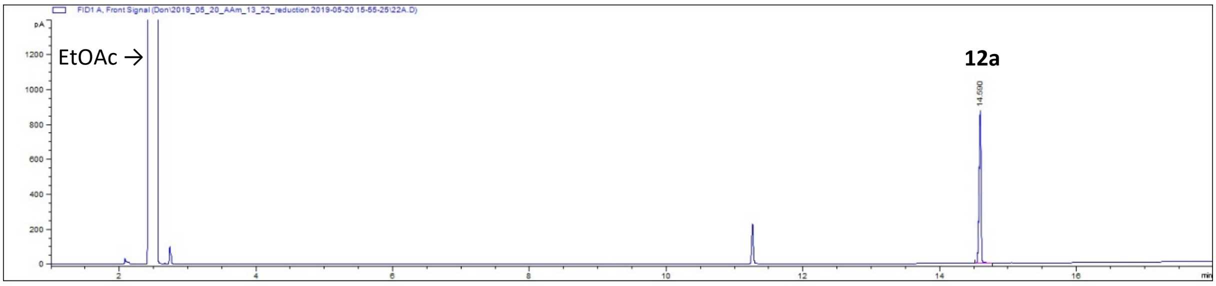

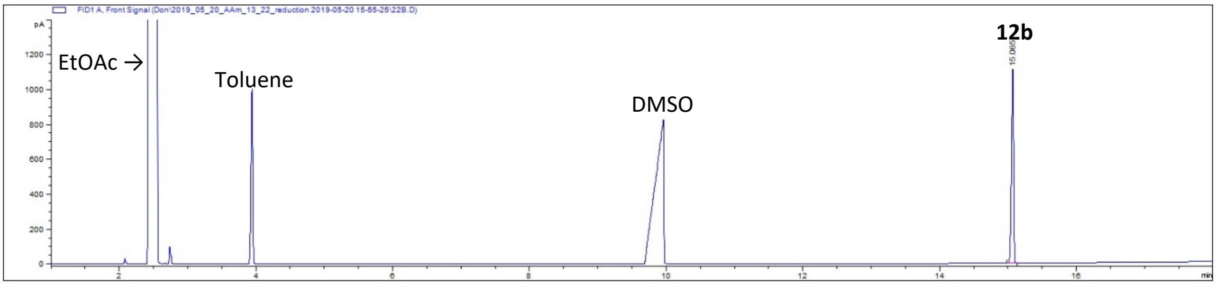

Biocatalytic reduction of **12b** catalyzed by LE-AmDH-v27

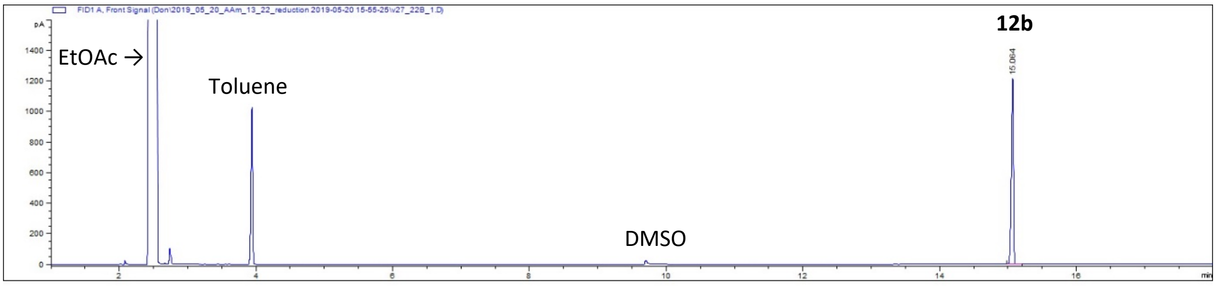

Reference compounds **13a** and **13b**

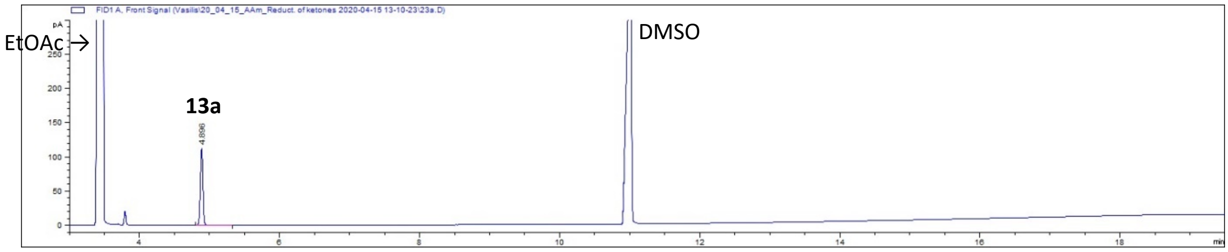

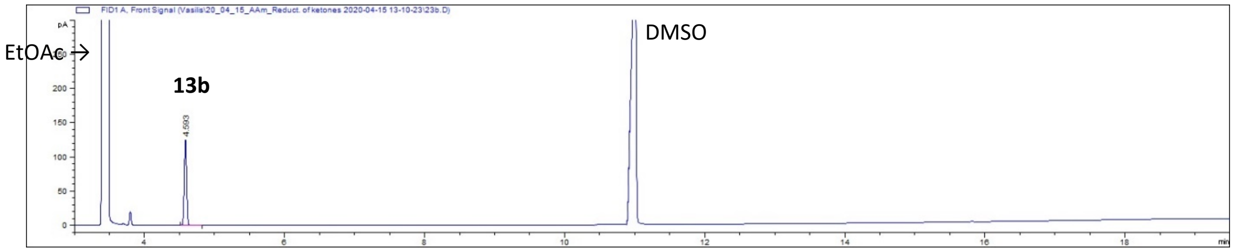

Biocatalytic reduction of **13b** catalyzed by LE-AmDH-v27

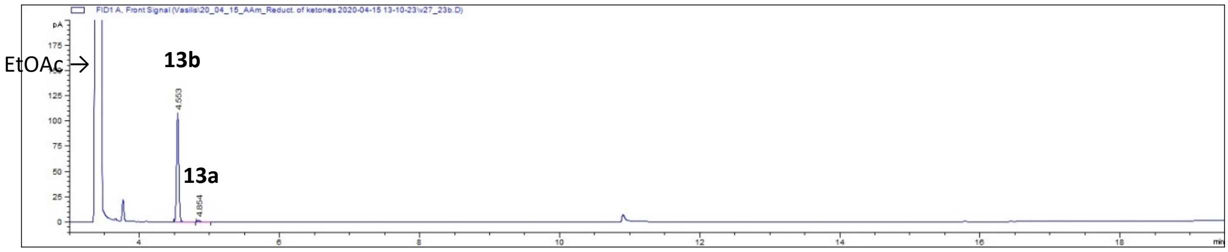

Reference compounds **14a** and **14b**

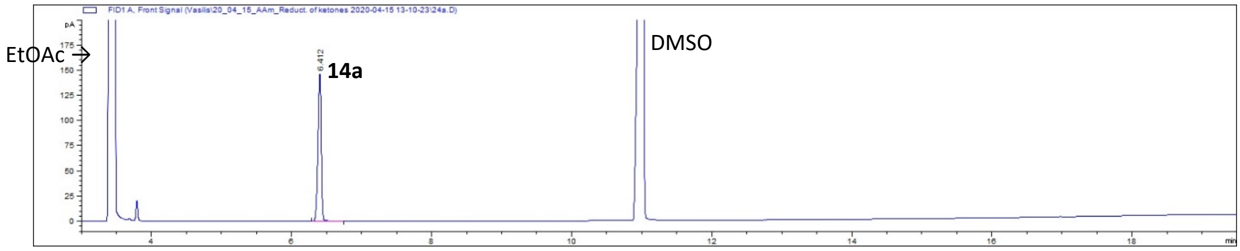

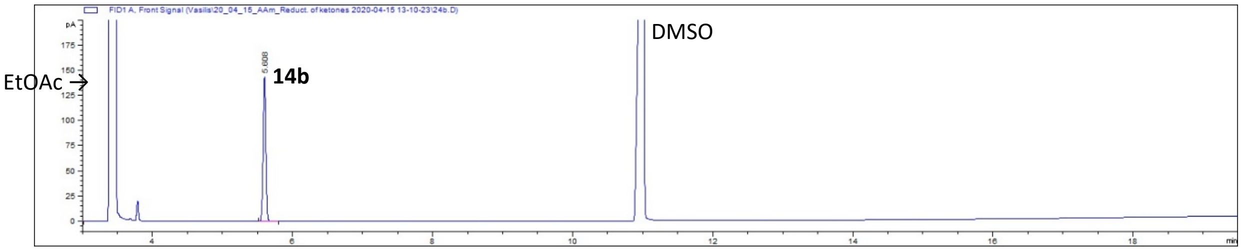

Biocatalytic reduction of **14b** catalyzed by LE-AmDH-v27

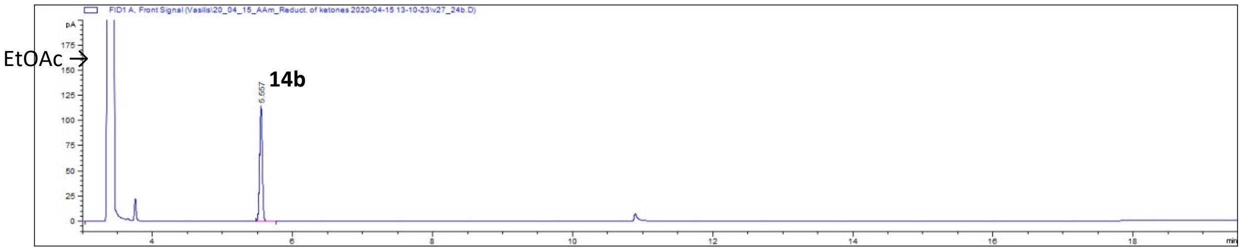

Reference compounds **15a** and **15b**

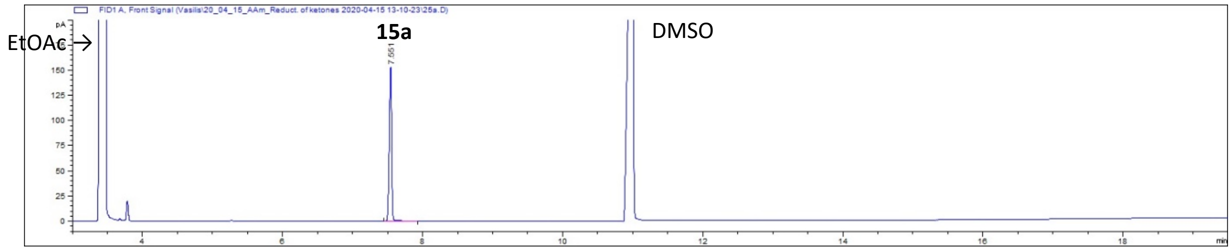

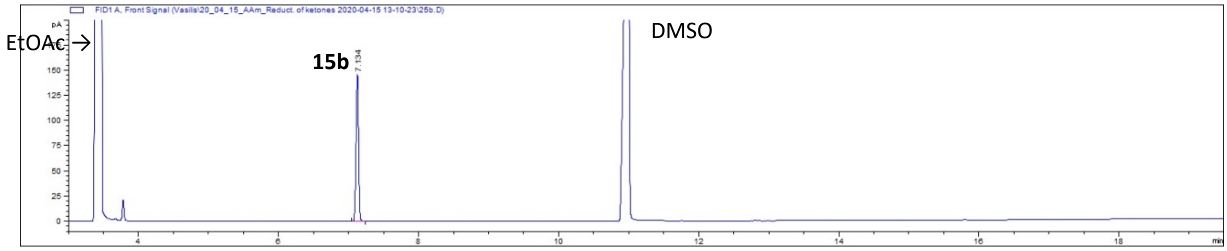

Biocatalytic reduction of **15b** catalyzed by LE-AmDH-v27

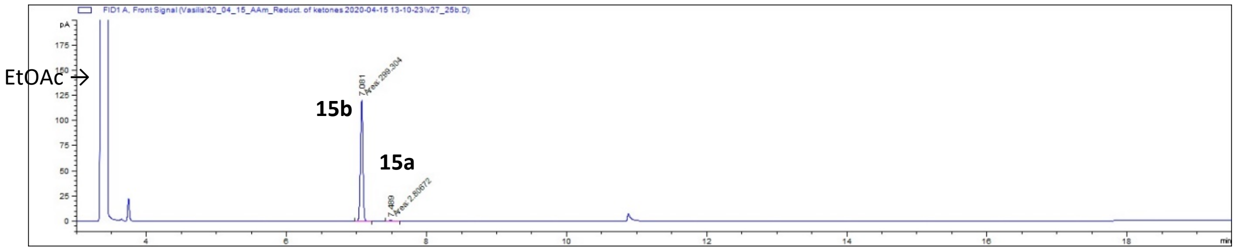

Reference compounds **16a** and **16b**

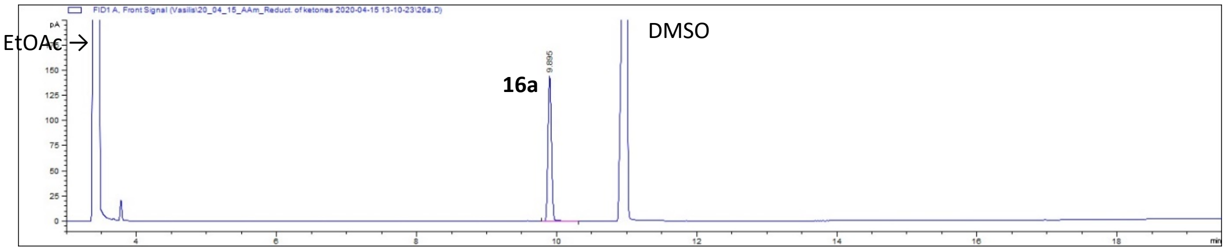

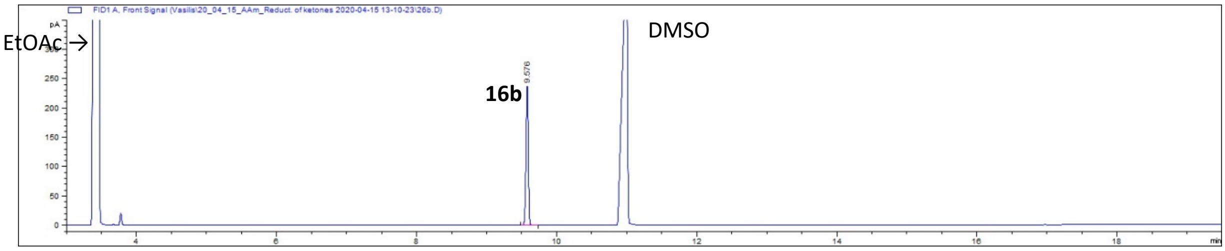

Biocatalytic reduction of **16b** catalyzed by LE-AmDH-v27

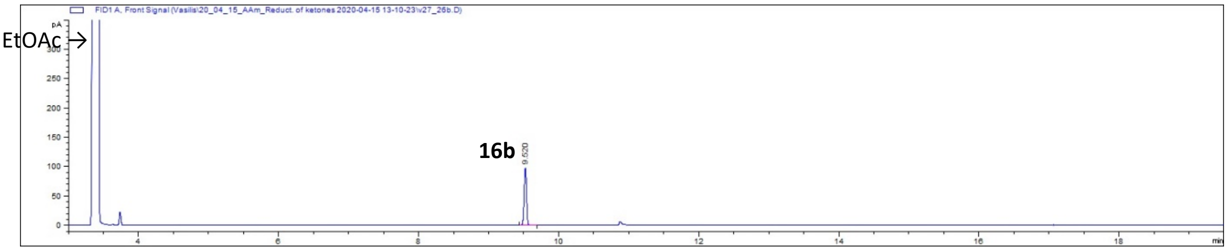

Reference compounds **17b**

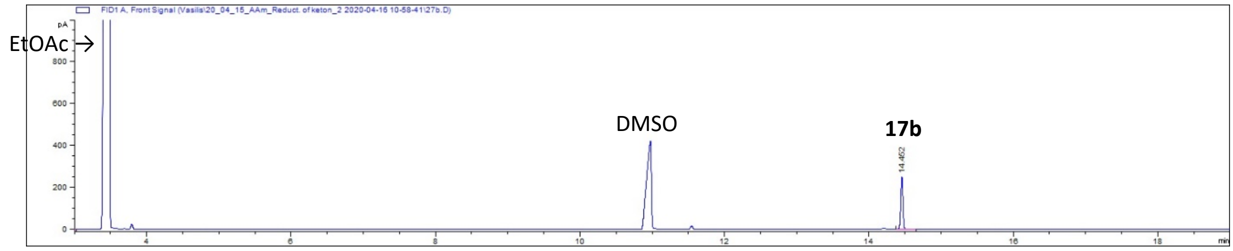

Blank reaction (NC1)

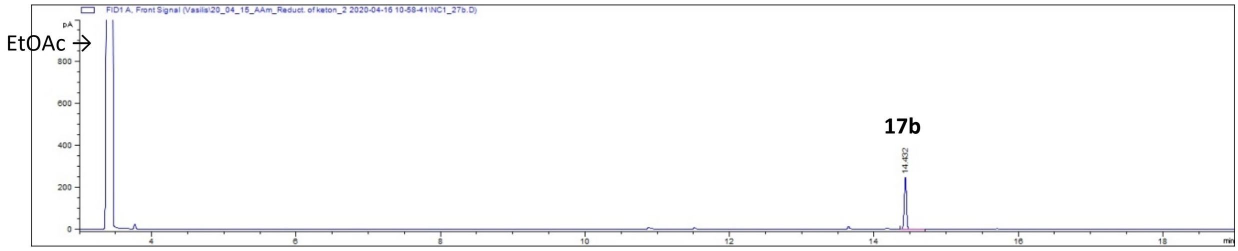

Biocatalytic reduction of **17b** using LE-AmDH-v27

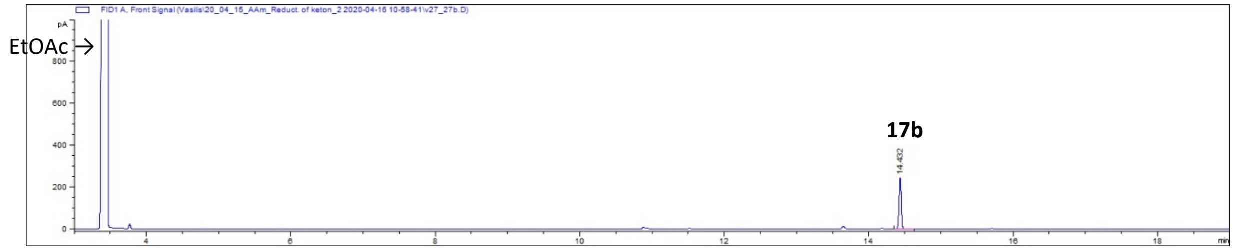

Reference compounds **18b**

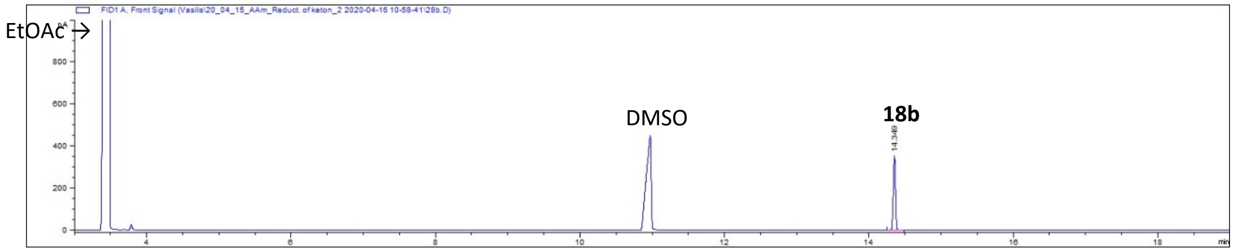

Blank reaction (NC1)

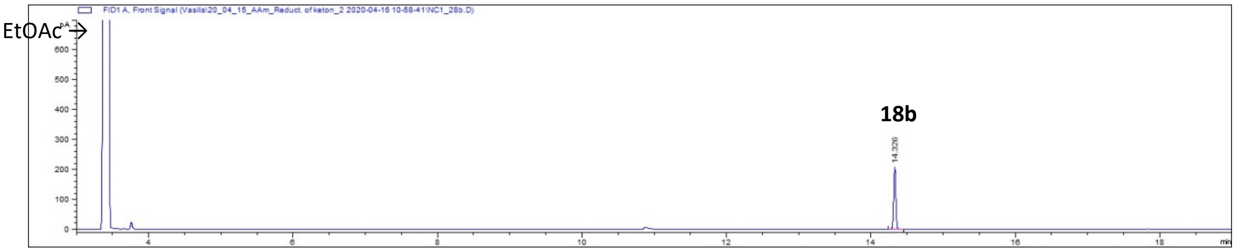

Biocatalytic reduction of **18b** using LE-AmDH-v27

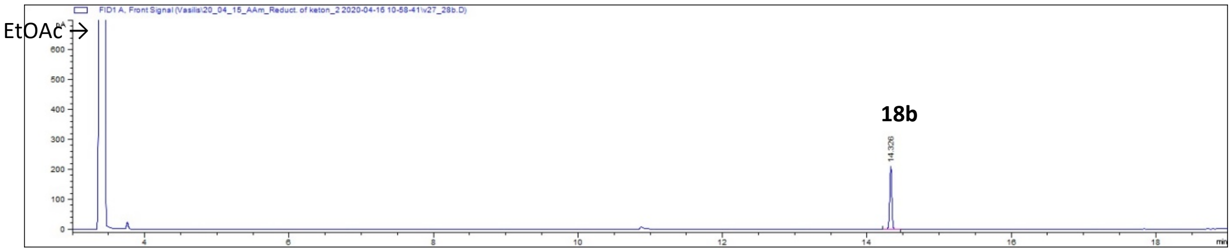

Reference compound **19b**

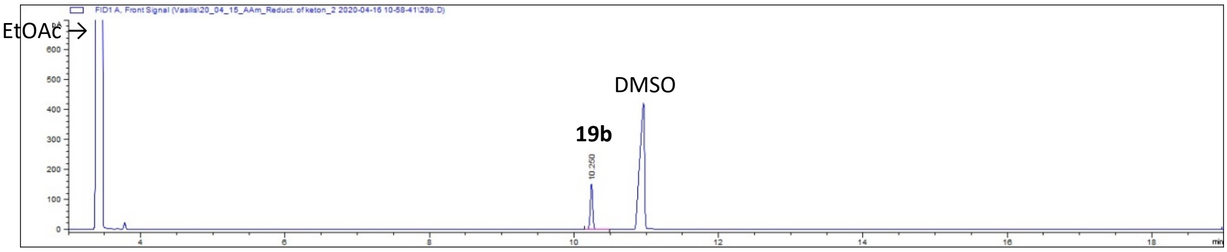

Blank reaction (NC1)

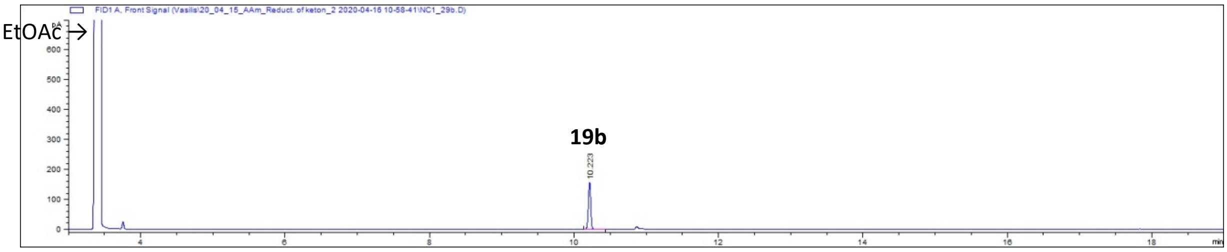

Biocatalytic reduction of **19b** using LE-AmDH-v27

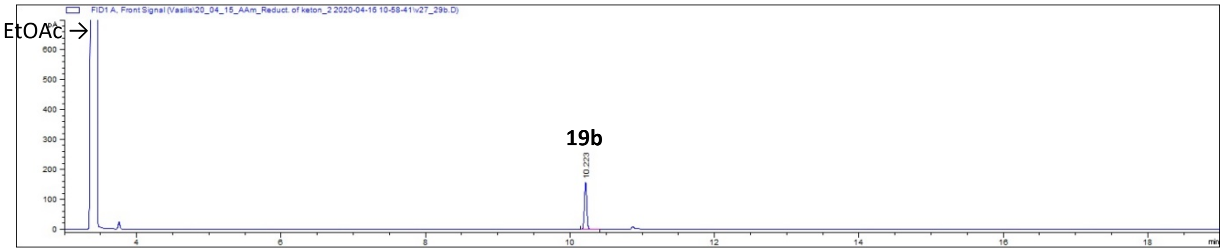

Reference compounds **20a** and **20b**

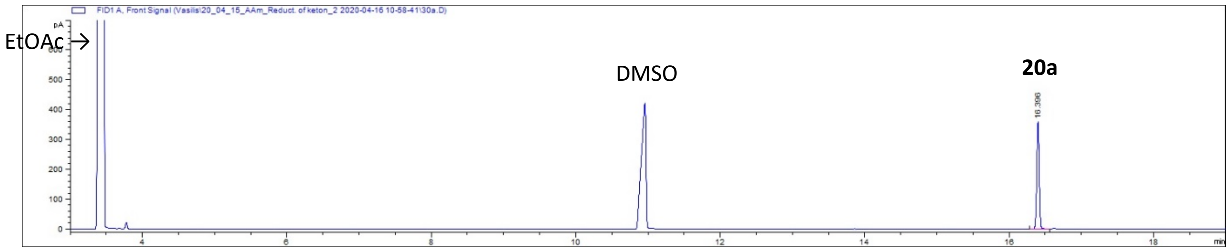

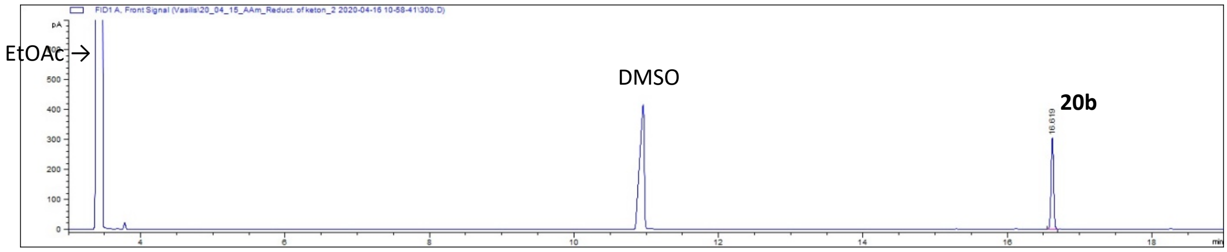

Biocatalytic reduction of **20b** using LE-AmDH-v27

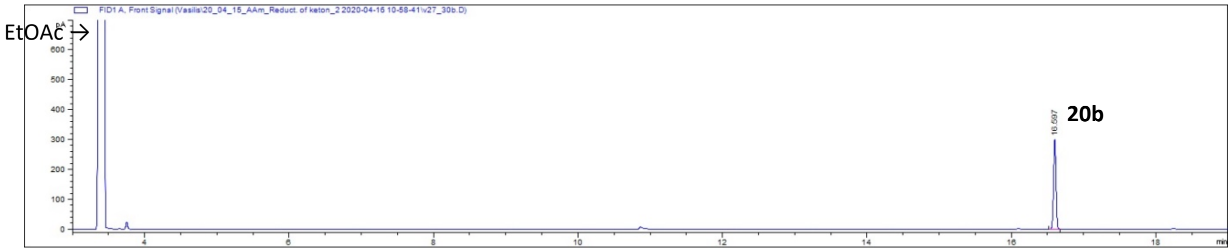

Reference compounds **21a** and **21b**

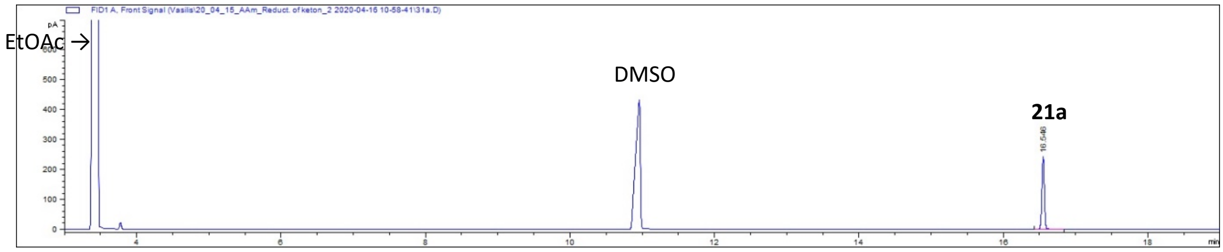

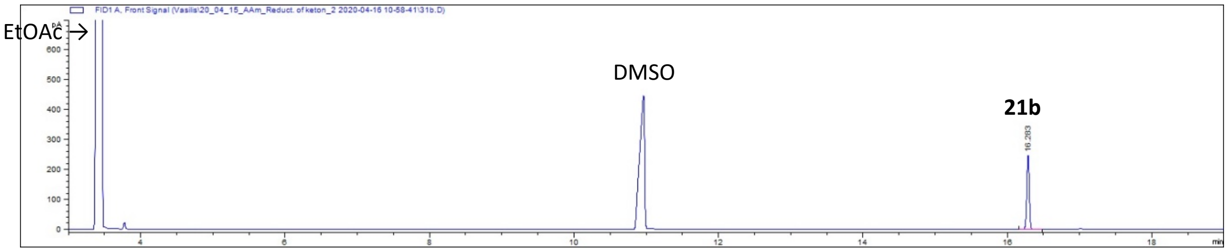

Biocatalytic reduction of **21b** using LE-AmDH-v27

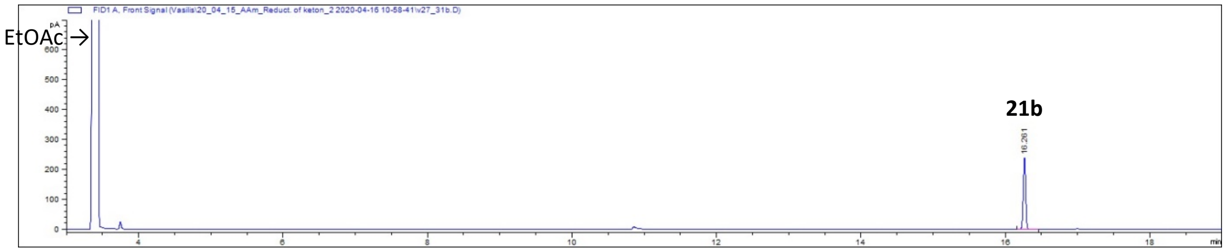

10.4 GC-FID chromatograms of oxidation of 1b to benzyl-alcohol

Reference compounds **1a** and **1b**

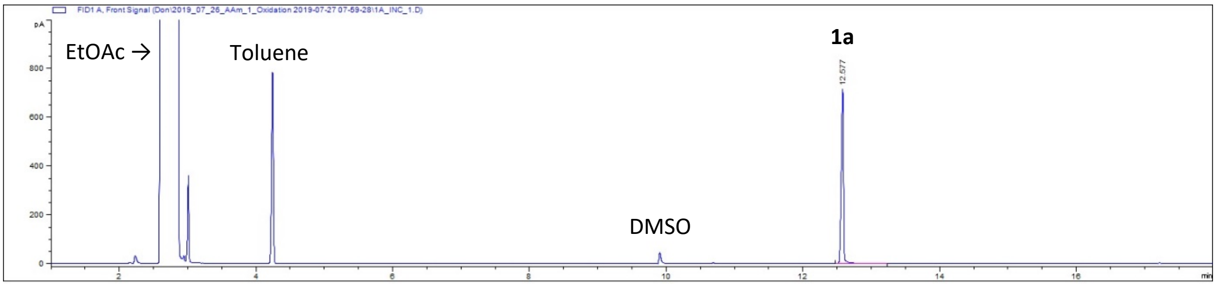

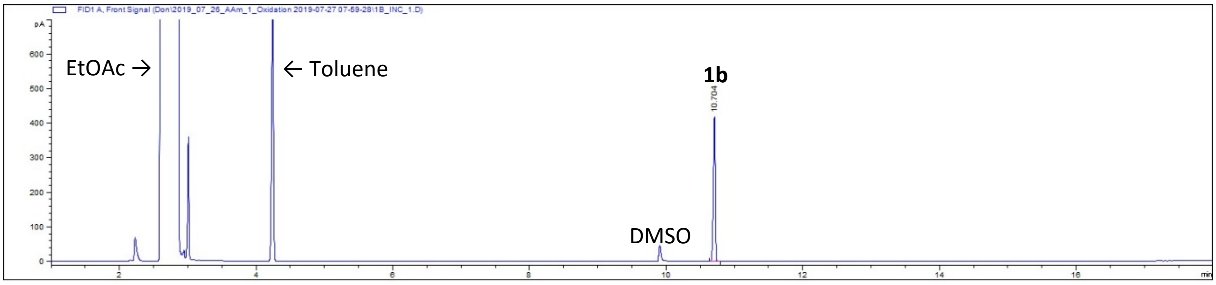

Biocatalytic oxidation of **1a** catalyzed by LE-AmDH-v27

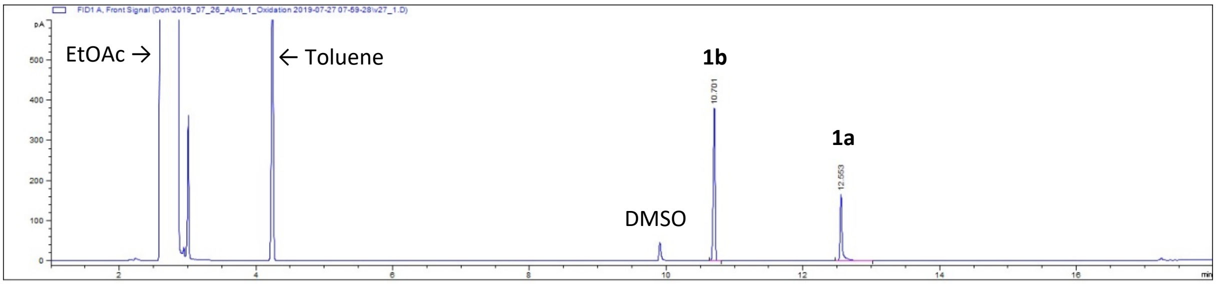

10.5 GC-FID chromatograms of cascade reactions

Reference compounds **1a**, **1b** and **1c**

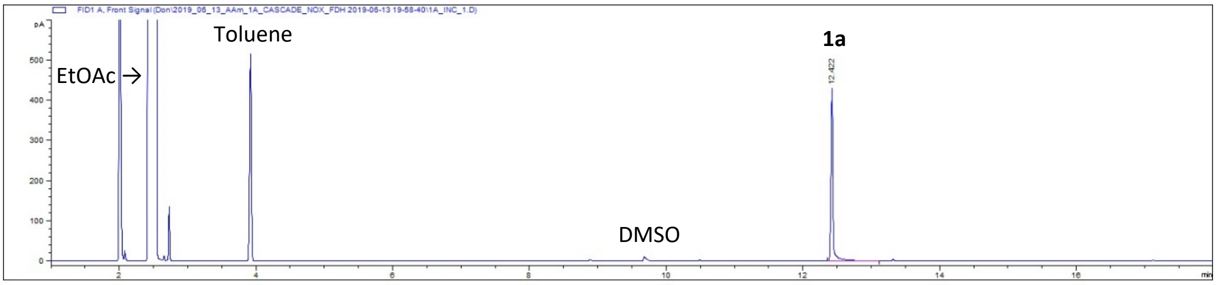

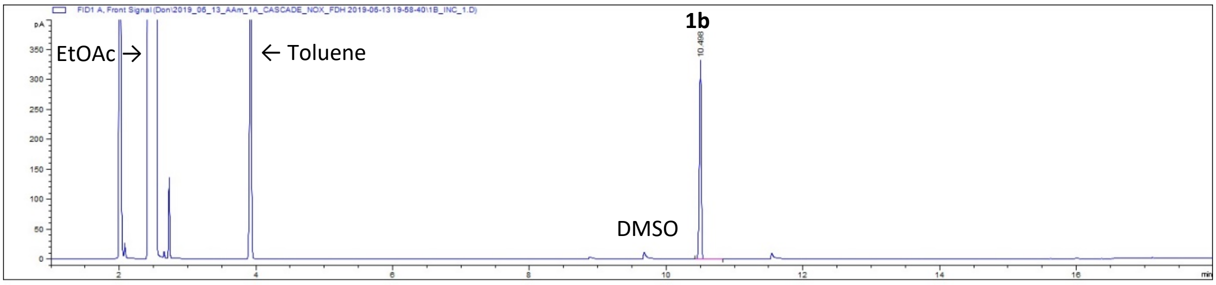

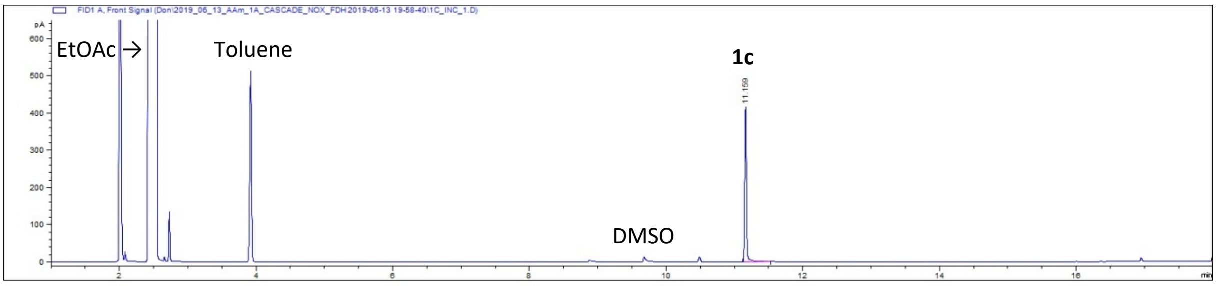

Single-enzyme hydrogen-borrowing amination of **1a** catalyzed by LE-AmDH-v1

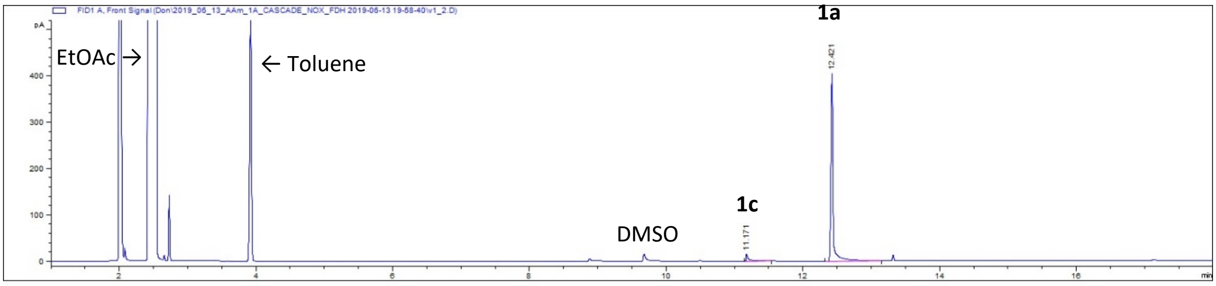

Biocatalytic network for the conversion **1a** to **1c** catalyzed by LE-AmDH-v27, NOx and Cb-FDH

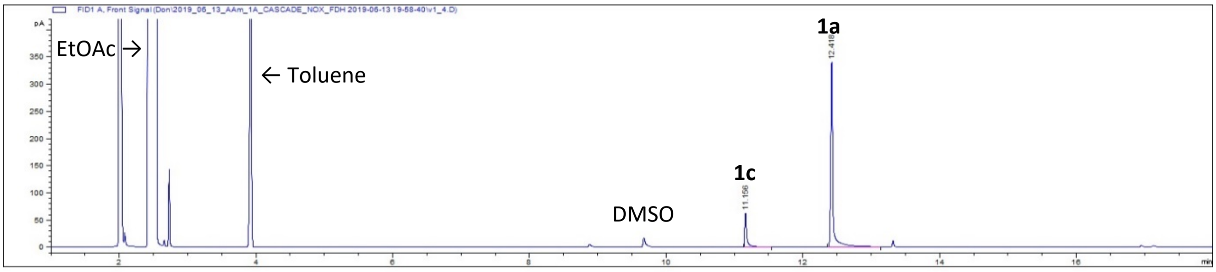

11. References

[1] V. Tseliou, T. Knaus, M. F. Masman, M. L. Corrado, F. G. Mutti, *Nat. Commun.* **2019**, *10*, 3717.

[2] J. Matsumoto, M. Higuchi, M. Shimada, Y. Yamamoto, Y. Kamio, *Biosci. Biotechnol. Biochem.* **1996**, *60*, 39-43.

[3] a) B. R. Bommarius, M. Schürmann, A. S. Bommarius, *Chem. Commun.* **2014** *50*, 14953-14955; b) F. G. Mutti, T. Knaus, N. S. Scrutton, M. Breuer, N. J. Turner, *Science* **2015**, *349*, 1525-1529; c) T. Knaus, W. Böhmer, F. G. Mutti, *Green Chem.* **2017**, *19*, 453-463.

[4] T. Knaus, V. Tseliou, L. D. Humphreys, N. S. Scrutton, F. G. Mutti, *Green Chem.* **2018**, *20*, 3931-3943.

[5] http://equilibrator.weizmann.ac.il/.

[6] R. A. Alberty, *Thermodynamics of biochemical reactions*, Wiley-Interscience, **2003**.
